# Reliance of Host-Encoded Regulators of Retromobility on Ty1 Promoter Activity or Architecture

**DOI:** 10.3389/fmolb.2022.896215

**Published:** 2022-07-01

**Authors:** Alicia C. Salinero, Simey Emerson, Tayla C. Cormier, John Yin, Randall H. Morse, M. Joan Curcio

**Affiliations:** ^1^ Laboratory of Molecular Genetics, Wadsworth Center, New York State Department of Health, Albany, NY, United States; ^2^ Department of Biomedical Sciences, School of Public Health, University at Albany, Albany, NY, United States

**Keywords:** Ty1, retrotransposon, long terminal repeat, promoter, retromobility, co-factor, restriction factor, *Saccharomyces cerevisiae*

## Abstract

The Ty1 retrotransposon family is maintained in a functional but dormant state by its host, *Saccharomyces cerevisiae*. Several hundred *RHF* and *RTT* genes encoding co-factors and restrictors of Ty1 retromobility, respectively, have been identified. Well-characterized examples include *MED3* and *MED15*, encoding subunits of the Mediator transcriptional co-activator complex; control of retromobility by Med3 and Med15 requires the Ty1 promoter in the U3 region of the long terminal repeat. To characterize the U3-dependence of other Ty1 regulators, we screened a library of 188 known *rhf* and *rtt* mutants for altered retromobility of Ty1*his3AI* expressed from the strong, TATA-less *TEF1* promoter or the weak, TATA-containing U3 promoter. Two classes of genes, each including both *RHF*s and *RTT*s, were identified. The first class comprising 82 genes that regulated Ty1*his3AI* retromobility independently of U3 is enriched for *RHF* genes that restrict the G1 phase of the cell cycle and those involved in transcriptional elongation and mRNA catabolism. The second class of 51 genes regulated retromobility of Ty1*his3AI* driven only from the U3 promoter. Nineteen U3-dependent regulators (U3DRs) also controlled retromobility of Ty1*his3AI* driven by the weak, TATA-less *PSP2* promoter, suggesting reliance on the low activity of U3. Thirty-one U3DRs failed to modulate P_*PSP2*_-Ty1*his3AI* retromobility, suggesting dependence on the architecture of U3. To further investigate the U3-dependency of Ty1 regulators, we developed a novel fluorescence-based assay to monitor expression of p22-Gag, a restriction factor expressed from the internal Ty1i promoter. Many U3DRs had minimal effects on levels of Ty1 RNA, Ty1i RNA or p22-Gag. These findings uncover a role for the Ty1 promoter in integrating signals from diverse host factors to modulate Ty1 RNA biogenesis or fate.

## Introduction

Retroviruses and retroviral-like transposable elements have compact genomes, yet their replication is a complex, multiphasic process. Replication involves reverse transcription of the RNA genome within ribonucleoprotein complexes, transport of the cDNA copy to the nucleus, integration of the cDNA into the host genome, transcription of the integrated provirus or element and transport of one or more RNA species to the cytoplasm for translation and packaging into viral or viral-like particles. A broad spectrum of host cellular factors are thought to be involved in carrying out or repressing retroelement replication; hundreds of host factors that are required for efficient replication or restriction of the HIV-1 retrovirus in human cells or the Ty1 long terminal repeat (LTR)-retrotransposon in the yeast *S. cerevisiae* have been identified in large-scale screens (Scholes et al., 2001; Griffith et al., 2003; Brass et al., 2008; Nyswaner et al., 2008; Zhou et al., 2008; Dakshinamurthy et al., 2010; Friedrich et al., 2011; Dziuba et al., 2012; Risler et al., 2012; Park et al., 2017). However, only a small fraction of these regulators has been validated in secondary screens or additional studies.

Ty1 elements are present in about 30 copies in common laboratory strains of *S. cerevisiae,* with most copies being autonomous elements. Ty1 encodes two sense-strand transcripts. Genomic RNA (gRNA), emanates from the promoter in the U3 region of the 5′ LTR; U3 contains a TATA box sequence (TATAAAAC) and is dependent on the SAGA (Spt-Ada-Gcn5-acetyltransferase) complex and the SWI/SNF chromatin remodeling complex for activation of transcription (Winston et al., 1984; Winston et al., 1987; Ciriacy et al., 1991; Happel et al., 1991; Winston and Carlson, 1992). Ty1 gRNA encompasses two open reading frames, *GAG*, which encodes the nucleocapsid precursor protein, p49-Gag, and *POL*, encoding enzymes required for retrotransposition- protease, integrase and reverse transcriptase. The second sense-strand transcript, Ty1i RNA, is initiated at an internal start site in the *GAG* ORF, approximately 800 bp downstream of the Ty1 gRNA transcription start site (Saha et al., 2015). The Ty1i promoter is architecturally distinct from that of U3; it does not have a canonical TATA box sequence, requires neither SAGA nor SWI/SNF for activity and is TFIID-dominated (Huisinga and Pugh, 2004; Salinero et al., 2018). Ty1i RNA is translated into p22-Gag, an N-terminally truncated form of p49-Gag that is incorporated into Ty1 VLPs and inhibits post-translational steps in Ty1 retrotransposition (Nishida et al., 2015; Saha et al., 2015; Tucker and Garfinkel, 2016). The ratio of Ty1 and Ty1i transcripts, one essential for retrotransposition and the other a potent inhibitor, is a critical determinant of Ty1 retromobility levels (Saha et al., 2015; Salinero et al., 2018).

Host genome-encoded factors that modulate replication of Ty1 are divided into two groups; Restrictors of Ty1 Transposition (RTTs), whose absence results in elevated retromobility, and Retrotransposition Host Factors (RHFs), whose depletion decreases retromobility (Curcio et al., 2015). Five genome-wide studies of non-essential genes and other studies focusing on a host factor gene or group of genes have led to the identification of these regulators (Lee et al., 1998; Rattray et al., 2000; Scholes et al., 2001; Griffith et al., 2003; Curcio et al., 2007; Nyswaner et al., 2008; Checkley et al., 2010; Dakshinamurthy et al., 2010; Dutko et al., 2010; Risler et al., 2012; Doh et al., 2014; Ho et al., 2015; Suresh et al., 2015; Ahn et al., 2017; Manhas et al., 2018; Salinero et al., 2018; Bonnet et al., 2021). There is limited overlap in genes identified among the genome-wide screens, possibly because of a high incidence of false positives or false negatives or because a large fraction of Ty1 regulators function only in specific conditions (Maxwell and Curcio, 2007a). For example, the function of some Ty1 regulators depends on the promoter from which the marked Ty1 element used to measure retromobility is expressed. Deletion of *NUP84*, *RAD52*, *XRS2*, *SPT21* or *RTF1* results in increased retromobility of a chromosomal Ty1 element driven from the U3 promoter but reduces retromobility of a plasmid-based Ty1 element driven from the strong *GAL1* promoter (Rattray et al., 2000; Griffith et al., 2003; Curcio et al., 2007; Nyswaner et al., 2008; Bonnet et al., 2021). In addition, several *RTT* and *RHF* genes regulate retromobility of Ty1*his3AI* when expressed from the U3 promoter but not when it is expressed from the *GAL1* or *TEF1* promoter, including *FUS3* (Conte et al., 1998), *RTT101* (Curcio et al., 2007), *LSM1* (Dutko et al., 2010)*, RPL1B* (Suresh et al., 2015) and *MED1, MED3, MED15* and *MED31* (Salinero et al., 2018). The latter four genes encode non-essential components of the Mediator transcriptional co-activator complex.

An earlier study from our laboratories demonstrated that the Mediator tail subunits Med2, Med3 and Med15 promote the preferential association of Mediator and RNA Polymerase II with U3 rather than the Ty1i promoter. In the absence of Med2, Med3 and/or Med15, Ty1 gRNA levels remain unchanged, but Ty1i RNA and p22-Gag levels increase and retromobility is >100-fold reduced. These and additional findings led us to propose a model wherein the U3 promoter is more reliant on a functional Mediator tail module than the Ty1i promoter. When the tail module was rendered nonfunctional, Mediator association with the U3 promoter was reduced relative to the Ty1i promoter, resulting in a relative increase in utilization of the Ty1i promoter. Replacing the weak, TATA-containing U3 promoter with the strong, TATA-less *TEF1* promoter allowed Ty1 transcription to be dominant over Ty1i transcription even when tail module subunits are deleted, resulting in equivalent levels of P_*TEF1*_-Ty1*his3AI* retrotransposition in the presence or absence of Med3 or Med15 (Salinero et al., 2018).

The work undertaken here has identified additional regulators of Ty1 retromobility whose functions require the U3 promoter, either because of its specific architecture or relative activity. Using a large collection of previously identified *rtt* and *rhf* mutants that was enriched for mutants with altered cDNA levels, we screened each mutant for retromobility of a *CEN*-plasmid-based P_*TEF1*_-Ty1*his3AI* element. Mutants that had higher or lower levels of P_*TEF1*_-Ty1*his3AI* retromobility relative to that in the wild-type strain were rescreened to determine whether their previously reported effects on Ty1 retromobility were recapitulated with a *CEN*-plasmid based Ty1*his3AI* element. These sequential screens identified one class of U3-independent regulators (U3IRs) of Ty1 retromobility and a second class of U3DRs. Further analysis of the latter class using the weak, TATA-less *PSP2* promoter to drive Ty1*his3AI* expression revealed two subsets of U3DRs: one that likely depends on the low efficiency of the U3 promoter, and the other that may require its architecture. Several U3DRs did not detectably influence the levels of Ty1 RNA, Ty1i RNA or p22 expression. Together, the findings suggest that the U3 promoter functions as a hub for integrating the influences of diverse co-factors and restriction factors on retromobility. Moreover, our study adds to a body of evidence supporting the idea that U3 recruits factors that associate with Ty1 RNA and affect its utilization at post-transcriptional steps in retromobility.

## Materials and Methods

### Yeast Strains and Media

*Saccharomyces cerevisiae* strains used in this study are BY4741 (*MATa*, *his3Δ1*, *leu2Δ0*, *met15Δ0*, *ura3Δ0*) and congenic strains with a single non-essential ORF replaced by a *kanMX* cassette (Brachmann et al., 1998; Giaever and Nislow, 2014). Six additional strains, each containing a *kanMX*-tagged DAmP allele of an essential gene (Breslow et al., 2008), were obtained from Thermo Scientific. Strain JC6464 is a derivative of BY4741 constructed by retrotransposition of Ty1*kanMXAI* into the host genome, as described previously. Strain JC6474 is the *med15∆::URA3* derivative of JC6464 (Salinero et al., 2018).

Yeast media used included Yeast Peptone Dextrose (YPD: 1% bactoyeast extract, 2% bactopeptone extract, 2% glucose) broth and agar. Mutant strains harboring deletion and hypomorphic alleles marked with *kanMX* were taken from frozen stocks in 15% glycerol and grown as single colonies on YPD agar containing 200 μg/ml Geneticin (G418) prior to transformation with plasmid DNA. A standard lithium acetate transformation protocol was used to introduce plasmid DNA into yeast strains (Gietz and Schiestl, 2007). Strains containing a plasmid were grown in Synthetic Complete (SC) broth [0.67% bactoyeast nitrogen base without amino acids (6.7 g/L), 0.08% SC dropout powder (0.8 g/L)] with 2% glucose as a carbon source unless otherwise indicated. SC dropout powder lacking leucine (SC-Leu), histidine (SC-His), leucine and histidine (SC-Leu-His), uracil (SC-Ura) or uracil and histidine (SC-Ura-His) were obtained from Sunrise Science Products.

### Plasmids

Plasmid-borne Ty1 elements used in this study carry the *his3AI-[∆1]* retrotransposition indicator gene (Scholes et al., 2001) cloned into the *Bgl*II site between the *pol* ORF and the 3′ LTR of Ty1-H3. DNA recombination between the *his3AI-[∆1]* allele and the *his3∆1* allele in strain BY4741 does not result in a functional *HIS3* allele.

Plasmid pBJC1250 consist of the *LEU2-CEN* vector pRS415 carrying the element P_*TEF1*_-Ty1*his3AI-[∆1]*, in which the U3 region of the 5′ LTR in Ty1-H3 is replaced with a *TEF1* promoter (P_*TEF1*_) (Salinero et al., 2018). P_*TEF1*_ was amplified with flanking *Apa*I and *Xho*I sites by PCR using plasmid pUG6 (Guldener et al., 1996) as template DNA and primers PTEFforward and PTEFreverse (Table 1). The PCR product was digested with *Apa*I and *Xho*I, and used to replace the *Apa*I-*Xho*I fragment containing the *GAL1* promoter on plasmid pBJC838 (Stamenova et al., 2009).

**TABLE 1:** Primers used in this study.

PTEF forward	AGT​CGC​GGG​CCC​TAG​GTC​TAG​AGA​TCT​GTT​TAG​CTT
PTEF reverse	GCT​AGT​CTC​GAG​TTG​TTT​ATG​TTC​GGA​TGT​GA
PJ749	GGT​TTT​CCG​TTT​ACT​GTC​GG
PJ762	GCA​ATG​GGC​CCT​GTT​GGA​ATA​GAA​ATC​AA
PJ763	CTA​GAA​GTT​CTC​CTC​GAG​GAT​TTA​GG
PJ795	CAT​CTA​AAT​TAG​TGG​AAG​CTG​AAA​CGC​AAG​G
PJ796	TAT​AGC​CTT​TAT​CAA​CAc​TGG​AAT​CCC​AAC​AAT​TAT​C
PJ797	GAT​AAT​TGT​TGG​GAT​TCC​AgT​GTT​GAT​AAA​GGC​TAT​A
PJ1480	ATA​GGG​CGA​ATT​GGG​TAC​CGG​GCC​CGA​CCC​AAC​ATC​AGA​AGA​CCC​AAG
PJ1481	GCT​ATA​ATA​TTA​GGT​ATA​CAG​AAT​ATA​CTA​GAA​GTT​CTC​CTC​AAT​TCT​TT
	GTG​GAG​ATA​AGC​TTT​AAA​GTC​TG

Plasmid pBDG633 consists of the *URA3-CEN* vector YCp50 carrying a functional, *his3AI-[∆1]*-tagged hybrid Ty1-912/Ty1-H3 element (Lee et al., 1998) in which the U3 region of the 5′ LTR of Ty1-912 is fused to the transcriptional start site of Ty1-H3, referred to as U3 (912)-Ty1*his3AI*. There are five nucleotide differences between the U3 domain of Ty1-912 and that of Ty1-H3, but none of these occur in a known regulatory motif (Clare and Farabaugh, 1985; Boeke et al., 1988). Plasmid pBDG633 was generously provided by Dr. David Garfinkel.

Plasmid pBJC998 consists of the *LEU2-CEN* vector pRS415 carrying a U3 promoter-driven Ty1*his3AI-[∆1]* element, herein referred to as U3(H3)-Ty1*his3AI*. The U3 region of the 3′ LTR of Ty1-H3 in plasmid pGTy1-Cla (Garfinkel et al., 1988) was amplified with flanking *Apa*I and *Xho*I sites using primers PJ762 and PJ763. The U3(H3) PCR fragment was digested with *Apa*I and *Xho*I and used to replace the *Apa*I-*Xho*I fragment on plasmid pBJC838 (Stamenova et al., 2009).

Plasmid pBJC1270 is a derivative of plasmid pLTR_p_:Gag_1-401_:GFP:*ADH1*_TER_ (Doh et al., 2014) in which the first AUG codon of the *GAG* ORF has been changed to CUG. Two PCR reactions were performed using pBJC998 DNA as a template and primers PJ795 and PJ797 or primers PJ796 and PJ749. The resulting PCR products were purified and then combined as a template for overlap extension PCR performed with primers PJ795 and PJ749. This final PCR product, containing the U5 region of the LTR and beginning of the *GAG* ORF, was digested with *Xho*I and *Hpa*I, and then used to replace the *Xho*I-*Hpa*I fragment in plasmid pLTR_p_:Gag_1-401_:GFP:*ADH1*_TER_. The *Xho*I-*Hpa*I fragment was sequenced to ensure that it contained only the intended nucleotide substitution.

Plasmid pBJC1279 consists of the *LEU2-CEN* vector pRS415 carrying a *PSP2* promoter-driven Ty1*his3AI-[∆1]* element, referred to as P_*PSP2*_-Ty1*his3AI*. The plasmid was constructed by amplifying the *PSP2* promoter (Chr XIII, nucleotide 235889–236513) from strain BY4741 genomic DNA using primers PJ1480 and PJ1481. The promoter was cloned into plasmid BJC838 digested with *Apa*I and *Xho*I using New England Biolabs NEBuilder^®^ HiFi DNA Assembly Master Mix.

### Semi-Quantitative Retromobility Patch Assay

As a screen for retromobility of P_*TEF1*_-Ty1*his3AI*, U3 (912)-Ty1*his3AI* and P_*PSP2*-_Ty1*his3AI* in different mutants, a semi-quantitative retromobility patch assay that measures the relative level of His^+^ papillation in cells in a ∼1 cm^2^ patch was performed. Patches of cells from four to seven independent transformants of plasmid pBJC1250, pBDG633 or pBJC1279 in strain BY4741 or a mutant derivative were spread on SC-Leu or SC-Ura agar, and plates were incubated at 30°C until patches of the mutant strains had grown to a similar density as patches of the wild-type strain BY4741 (2–4 days). The SC-Leu and SC-Ura plates were photographed, and then replicated to YPD agar and grown at 20°C for three to 6 days. After photographing the YPD plates, they were replicated to SC-His-Leu or SC-His-Ura agar, incubated for 4 days at 30°C., and photographed.

### Quantitative Assay of P_*GAL1*_-Ty1*his3AI* Retromobility

Single colonies of strain BY4741 and mutant derivatives harboring plasmid pBJC838 (Stamenova et al., 2009), which carries the *P*_*GAL1*_ *-Ty1his3AI* element, were grown in SC-Leu broth at 30°C overnight. Cells were diluted 1:100 in SC-Leu + 2% raffinose + 2% sucrose + 2% galactose and grown for 3 days at 20°C. A 1 or 2 µl aliquot was plated on SC-Leu agar and an aliquot of 10–1,000 µl was plated on SC-Leu-His agar. Plated cells grew at 30°C for three to 5 days. Colonies were counted, and the retromobility frequency of each culture was calculated as the number of His^+^ Leu^+^ colonies divided by the total number of Leu^+^ colonies in 1 ml of culture. The mean retromobility frequency (MRF) of seven independent transformants of the same genotype was determined. The relative retromobility frequency of each strain is the mean retromobility frequency (MRF) of seven independent transformants of the mutant compared to the mean retromobility frequency (MRF) of seven independent transformants of the wild-type strain measured in parallel at the same time.

### Quantitative Assay of Ty1*kanMXAI* and P_*PSP2*_-Ty1*his3AI* Retromobility

Independent transformants of strain JC6464 and JC6474 harboring plasmid pBJC1279 were grown overnight in SC-Leu broth at 30°C. Cultures were diluted 1:1,000 in YPD broth and grown at 20°C for 3 days. A 1:1,000 dilution of each culture was plated on YPD agar to determine the number of cells (colony forming units) in each culture. Aliquots of each culture were plated on YPD agar with 200 μg/ml Geneticin to determine the number of G418^R^ colony forming units per culture, and on SC-His agar to determine the number of His^+^ colony forming units per culture. Plates were grown for 3 days at 30°C before counting colonies. The Ty1*kanMXAI* retromobility frequency was calculated for each transformant as the number of G418^R^ colonies divided by the total number of cells in an equal volume of culture, and the Ty1*kanMXAI* mean retromobility frequency (MRF) was the average from six independent transformants. The P_*PSP2*_-Ty1*his3AI* retromobility frequency was calculated for each transformant as the number of His^+^ colonies divided by the total number of cells in an equal volume of culture, and the P_*PSP2*_-Ty1*his3AI* mean retromobility frequency (MRF) was the average from the same six independent transformants.

### Data Analysis

Gene ontology enrichment analysis was performed using the Gene Ontology Term Finder at the *Saccharomyces* genome database (SGD) (Cherry et al., 2012). Relative growth rates for 1,312 mutant strains was downloaded from (O'Duibhir et al., 2014). *p*-values using the Mann-Whitney *U*-test were calculated using the online calculator at https://astatsa.com/WilcoxonTest/. Previously published ChIP-seq data used in Supplementary Presentation 1 is archived at the NCBI BioProject database (https://www.ncbi.nlm.nih.gov/bioproject/) under accession number SRP047524 (Paul et al., 2015) and PRJNA657372 (Sarkar et al., 2022).

### RNA Purification and Northern Blotting

Yeast was grown in YPD broth at 30°C overnight. Cultures were diluted to an OD_600_ of 0.1 in 50 ml YPD broth and incubated at 20°C until an OD_600_ of 0.6–0.8 was reached. Cells were pelleted, and pellets were washed in water before being frozen on dry ice and stored at −80°C. Total cellular RNA was isolated using a hot phenol extraction method (Collart and Oliviero, 2001). Between 250 µg and 1 mg of total cellular RNA was used to purify polyA^+^ RNA with the Magnetic mRNA Isolation Kit (New England Biolabs) following the manufacturer’s protocol. Purified PolyA^+^ RNA (6 µg) was incubated in glyoxal loading dye (Ambion) for 30 min at 50°C. and fractionated on a 0.8% SeaKemGTG agarose gel. RNA was transferred to a HybondXL membrane (Amersham) utilizing a SSC gradient transfer system between 6x SSC and 10× SSC overnight at room temperature. Following transfer, membranes were UV crosslinked using the Optimal Crosslinking setting on the Spectrolinker XL-1500 (Spectronics Corporation). Membranes with bound RNA were stored at 4°C in 5× SSC.

Riboprobes labeled with α-^32^P-CTP were synthesized using an SP6 polymerase *in vitro* transcription reaction (Promega). The template for the antisense riboprobe that hybridizes to Ty1 gRNA and Ty1i RNA was plasmid pGEM-TyA1 digested with *XhoI* (Curcio et al., 1990); for the antisense riboprobe that hybridizes to *PYK1* RNA, plasmid pGEM-PYK1 digested with *ClaI* (Curcio and Garfinkel, 1992). Riboprobes purified on a NucAway spin column (Thermo Fisher) were added to membranes in 25 ml NorthernMax Prehybridization buffer (Ambion) and allowed to hybridize to membranes overnight at 65°C. Membranes were washed in 2× SSC, 0.1% SDS for 45 min at 65°C, 0.1× SSC, 0.1% SDS for 45 min at 65°C and twice in 0.1% SDS for 10 min at 65°C. Bands were imaged on a Typhoon 9400 phosphoimager and relative quantifications were obtained using ImageQuant software (Molecular Dynamics). Membranes probed with a Ty1 riboprobe were stripped by washing twice in boiling 0.1% SDS incubated at 70°C for 10 min. Stripped membranes were then re-probed to detect *PYK1* mRNA, and the signal quantitated as above.

### Flow Cytometry Assay of p22-Gag:GFP Activity

Strain BY4741 and selected mutant derivatives were transformed with the vector, pRS415 (Sikorski and Hieter, 1989) or plasmid pBJC1270. Three independent transformants of pRS415 and another three of pBJC1270 in each strain were grown for 18–24 h in SC-Leu broth at 30°C. The OD_600_ of each culture was determined, and cells were diluted in SC-Leu broth to an OD_600_ of 0.2. Diluted cultures were grown at 20°C for 3 hours before measuring fluorescence with a FACSCalibur (Becton Dickinson) flow cytometer. The geometric mean of fluorescence activity in 10,000 cells was measured for each pBJC1270 transformant and each pRS415 transformant. The geometric means of three pRS415 transformants of a specific strain were averaged to obtain a value for autofluorescence of that strain. This value was subtracted from the geometric mean of each of three pBJC1270 transformants in that strain, and the three resulting values were averaged to obtain the p22-Gag:GFP activity for each strain.

## Results

### Screen of *RHF* and *RTT* Genes for U3-Independence of Retromobility Phenotype

**FIGURE 1:**
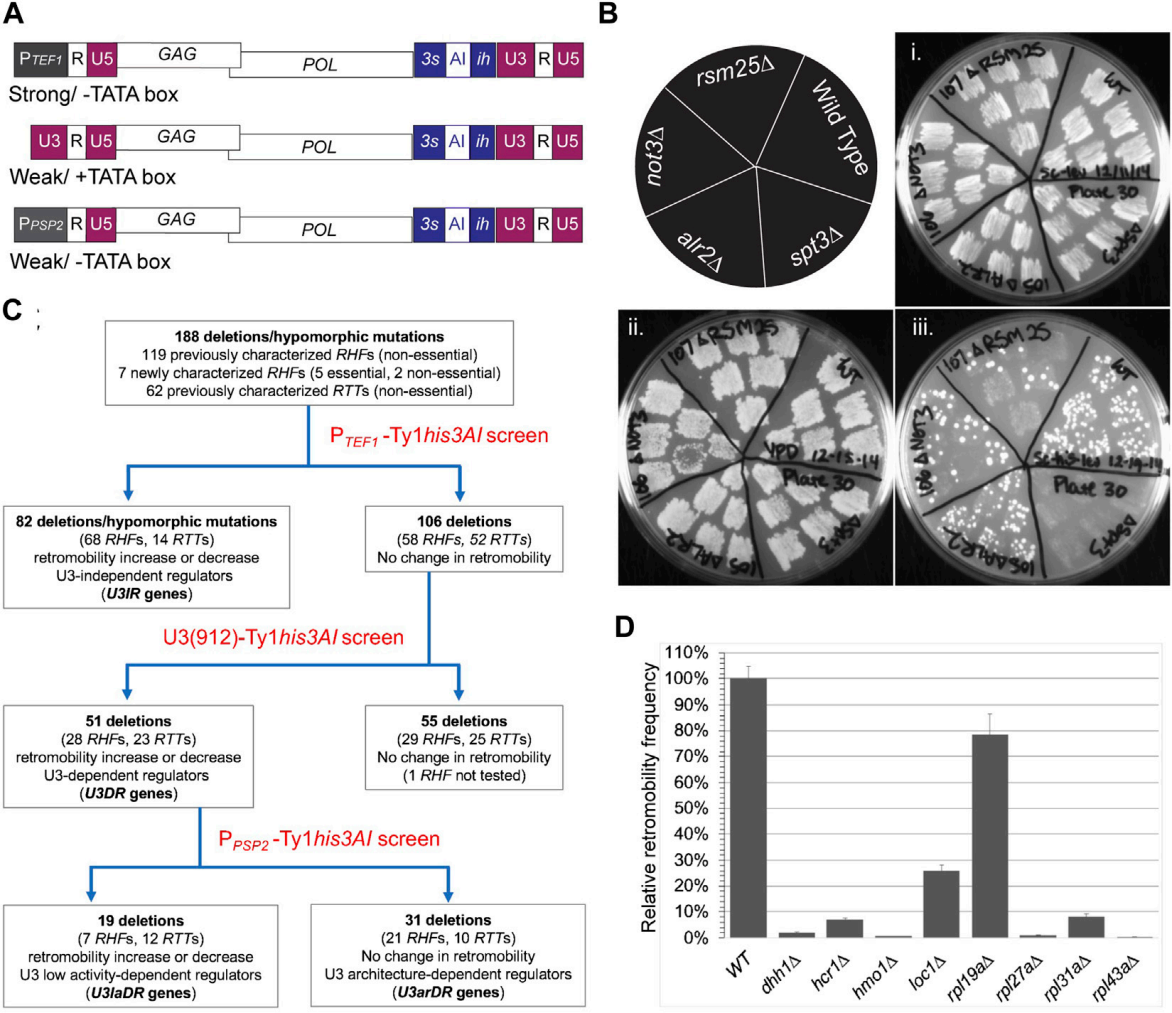
Serial retromobility assay screen identifies U3 promoter independent and dependent regulators of Ty1 retromobility. **(A)** Schematic of P_*TEF1*_-Ty1*his3AI*, U3 (912)-driven Ty1*his3AI* and P_*PSP2*_-Ty1*his3AI* elements introduced into host mutants on *CEN*-plasmids and used in semi-quantitative retromobility assays. Indicated below each schematic is the relative strength of the promoter (“strong” or “weak”) and the presence (+TATA box) or absence (−TATA box) of a consensus TATA box sequence. Promoters were fused to the 5′ end of the R domain of the 5′ LTR, which defines the transcription start site. **(B)** One set of plates from P_*TEF1*_-Ty1*his3AI* screen. Schematic in upper left corner shows host strain genotypes analyzed; panel **(i)** shows six independent transformants of each strain carrying the *CEN*- P_*TEF1*_-Ty1*his3AI* plasmid patched on SC-Leu agar plate and incubated at 30°C; panel **(ii)** shows the same patches following replica plating to YPD agar and growth at 20°C to induce retromobility; panel **(iii)** shows the same patches following replica plating to SC-His-Leu agar and growth at 30°C to visualize colonies from cells that retained the *LEU2*-marked plasmid and sustained a *HIS3*-marked retromobility event. The wild-type strain BY4741 and *spt3∆* mutant are controls. Two examples of mutants with reduced P_*TEF1*_-Ty1*his3AI* retromobility (*rsm25∆* and *not3∆*) and one with a wild-type retromobility (*alr2∆*) are also included. **(C)** Flow chart of serial retromobility screens performed and host mutants in each category. The 188 *RTT* and *RHF* mutants analyzed were originally identified using a Ty1*his3AI* element with the U3 promoter driving transcription. **(D)** Results of quantitative assay for retromobility of *CEN* plasmid-based P_*GAL1*_-Ty1*his3AI* element in seven independent transformants of eight *u3ir-rhf* mutants. Relative retromobility frequency is the mean retromobility frequency (MRF) in each mutant as a percentage of the MRF in the wild-type strain. Error bars, standard error.

To identify *RTT* and *RHF* genes that regulate Ty1 retromobility via the U3 promoter, mutant alleles were tested for their effects on retromobility of a Ty1*his3AI* element in which the U3 promoter was replaced with the *TEF1* promoter (P_*TEF1*_; Figure 1A). The TATA-less, TFIID-dominated *TEF1* promoter was chosen because it has a different architecture than the TATA-containing, SAGA-dependent U3 promoter and because *P*_*TEF1*_ is a strong promoter (Schirmaier and Philippsen, 1984; Lee et al., 2015), whereas U3 is relatively weak (Morillon et al., 2002) (Supplementary Presentation 1). The P_*TEF1*_ promoter was fused to the Ty1-H3 element at the transcription start site such that only U3 and no transcribed sequences are deleted in P_*TEF1*_-Ty1*his3AI*. The retrotransposition indicator gene, *his3AI* allows retromobility of a Ty1 element to be measured in a simple, quantitative genetic assay; it consists of an artificial intron (AI) within the coding region of the *HIS3* gene in an antisense orientation. The *his3AI* gene is inserted in the 3′ untranslated region of the Ty1-H3 element in the opposite transcriptional orientation (Figure 1A); therefore, the AI cannot be spliced from the *his3AI* transcript but is in the correct orientation to be spliced from Ty1*his3AI* RNA. The use of a spliced Ty1*his3* transcript as a template for reverse transcription generates cDNA in which the functional *HIS3* allele is restored. Integration of this Ty1*HIS3* cDNA into the host genome, either by integration or by recombination of the cDNA with preexisting Ty1 sequences in the genome, allows for expression of *HIS3* and renders the cell phenotypically His^+^. The frequency of His^+^ prototroph formation is a direct measure of Ty1*his3AI* retrotransposition or cDNA recombination, together known as retromobility (Curcio and Garfinkel, 1991).

A *CEN*-based, low copy plasmid harboring the P_*TEF1*_-Ty1*his3AI* element was introduced into strains bearing mutant alleles of 188 *RTT* and *RHF* genes (Supplementary Table 1). Most of the *rtt* and *rhf* mutations analyzed were deletions of non-essential genes identified in one or more of four large-scale screens that used a chromosomal or integrating plasmid-based Ty1*his3AI* element as a retromobility reporter (Scholes et al., 2001; Nyswaner et al., 2008; Dakshinamurthy et al., 2010; Risler et al., 2012). Retromobility host factors that were identified in a screen employing a *GAL1* promoter-driven Ty1 element were excluded from our study, since by definition, these host factors do not require the Ty1 promoter to control retromobility (Griffith et al., 2003). Like Med3 and Med15, many of the RHFs and RTTs identified in the four Ty1*his3AI* screens impact Ty1 cDNA levels without substantially altering levels of Ty1 RNA or its primary translation product, Gag (Lee et al., 1998; Rattray et al., 2000; Scholes et al., 2003; Sundararajan et al., 2003; Curcio et al., 2007; Dutko et al., 2010; Risler et al., 2012; Salinero et al., 2018). To increase the likelihood of identifying U3-dependent regulators, we excluded *rtt* and *rhf* mutants that were reported to have no change in cDNA levels, since these mutations are more likely to affect Ty1 cDNA utilization than a potentially promoter-dependent step such as transcription or RNA fate. Two additional non-essential genes analyzed were newly identified *RHF* genes, *CAF130* and *NOT3*, which encode subunits of the CCR4-NOT complex, a master regulator of gene expression from transcription to mRNA degradation (Collart, 2016; Buschauer et al., 2020). Hypomorphic DAmP alleles of six essential *RHF* genes were also analyzed. DAmP alleles harbor the *kanMX* gene inserted into the 3′ untranslated region of the essential gene, thereby destabilizing and reducing the steady-state level of mRNA produced (Breslow et al., 2008). The six essential *RHF* genes include *SRP68* (Doh et al., 2014) and five others that we identified in a small screen of essential genes related to known regulators of retromobility (*CDC36/NOT2*, *CDC39/NOT1*, *MSL5*, *NDC1*, *RPB7*). As controls, mutants harboring a deletion of *CHK1*, which has no effect on Ty1 retrotransposition (Curcio et al., 2007), or *SPT3*, which encodes a SAGA complex component that activates transcription of Ty1 and represses transcription of Ty1i (Winston et al., 1984; Grant et al., 1997; Saha et al., 2015), were included in our analysis.

A semi-quantitative patch assay was used to measure retromobility in four to six transformants of the *CEN*- P_*TEF1*_-Ty1*his3AI* plasmid in wild-type and mutant strains (Figure 1B). Transformants, grown initially as small patches of cells at 30°C on SC-Leu agar to maintain the plasmid, were replicated to YEPD agar and grown at 20°C, as these are optimal conditions for retromobility. Growth at 20°C continued until patches of mutant cells and wild-type cells reached equivalent densities. Patches were then replicated to SC-Leu-His agar and incubated at 30°C to allow growth of cells that had retained the plasmid and sustained a retromobility event. The level of His^+^ Leu^+^ papillation in transformants of each mutant was compared to that in the wild-type strain grown on the same plate (Figure 1B). Transformants of the *spt3∆* mutant were included on each plate as an additional control. Images of mutants are shown in Supplementary Data Sheet 1. Classification of mutants and test plate number for each mutant is provided in Supplementary Table 1.

The *spt3∆* mutant had few or no His^+^ Leu^+^ papillae on all P_*TEF1*_-Ty1*his3AI* screen plates (Figure 1B; Supplementary Data Sheet 1); the difference in His^+^ papillae numbers between the wild-type strain and the *spt3∆* mutant provides a partial range of the assay for each plate. The *chk1∆* mutant, which has no retromobility defect (Curcio et al., 2007), had wild-type levels of P_*TEF1*_-Ty1*his3AI* retromobility, as expected (Plate 42, Supplementary Data Sheet 1). Based on prior studies, both the *dhh1∆* mutant and the *srp68-DAmP* mutant were expected to have reduced P_*TEF1*_-Ty1*his3AI* retromobility, since Dhh1 is required for P_*GAL1*_-driven retromobility of Ty1*his3AI* (Dutko et al., 2010), and Srp68 is required co-translationally for Ty1*his3AI* retromobility (Doh et al., 2014). Indeed, both genes were required for efficient retromobility of P_*TEF1*_-Ty1*his3AI* (Plates 55 and 63, respectively; Supplementary Data Sheet 1).

The P_*TEF1*_-Ty1*his3AI* screen identified 82 of the 188 *rhf* and *rtt* mutants as having a level of retromobility that was either higher or lower than that of the wild-type strain, using a threshold of ∼2-fold increase or decrease in His^+^ Leu^+^ papillae (Figure 1C; Supplementary Table 1). The corresponding factors regulate retromobility regardless of whether Ty1*his3AI* RNA is driven by P_*TEF1*_ or U3, and thus were classified as U3-Independent Regulators, or U3IRs. 76 mutants (including *dhh1∆* and *srp68-DAmP*) were previously reported regulators of Ty1*his3AI* retromobility. Mutants harboring DAmP alleles of all five newly identified, essential *RHF* genes, *CDC36/NOT2*, *CDC39/NOT1*, *MSL5*, *NDC1* and *RPB7*, are also included in the cohort of 82*.* The final one is *not3∆*, another newly identified *rhf* mutant. Greater than 80% of these U3IRs are retromobility co-factors.

As an additional test of the idea that U3IRs regulate retromobility regardless of the strength or architecture of the promoter driving Ty1*his3AI* expression, retromobility was measured quantitatively in eight *rhf-u3ir* mutants using a *CEN*-plasmid-based Ty1*his3AI* element driven from the inducible *GAL1* promoter (P_*GAL1*_; Figure 1D). P_*GAL1*_ is a strong promoter like P_*TEF1*_, but it contains a TATA box, like U3. Retromobility of P_*GAL1-*_Ty1*his3AI* in the *dhh1∆* mutant was 2% of that in a wild-type strain, consistent with previous results (Dutko et al., 2010), and less than 10% of wild-type in the *hcr1∆*, *hmo1∆*, *rpl27a∆*, *rpl31a∆* or *rpl43a∆* mutants. Retromobility in the *loc1∆* and *rpl19a∆* mutant was 26 and 78% of that in the congenic wild-type strain, respectively; these decreases were small but significant (*p* < 0.00001 and *p* < 0.05, respectively; Student’s *t*-test). Together with the results of the P_*TEF1*_ *-*Ty1*his3AI* assay, these findings suggest that U3IRs act in a manner that is at least partially independent of the expression level of Ty1 RNA and the architecture of the U3 promoter.

### Identification of U3-Dependent Regulatory Genes

A subset of 106 *rtt* and *rhf* mutants had no detectable defect in P_*TEF1*_-Ty1*his3AI* retromobility relative to the wild-type strain (Figure 1C). To identify the members of this set whose phenotype reflects the substitution of P_*TEF1*_ for U3 and not the Ty1*his3AI* element being located on an extrachromosomal plasmid, we tested retromobility of a U3-driven Ty1*his3AI* element on a *CEN*-based plasmid in 105 of the 106 *rtt* and *rhf* mutants and the *chk1∆* negative control (Supplementary Table 2). One mutant, *erg6∆*, could not be transformed with the U3 (912)-Ty1*his3AI* plasmid in multiple attempts. Transformants were grown as patches on SC-URA agar at 30°C, replicated to YPD agar and grown at 20°C, and then replicated to SC-Ura-His agar to select for His^+^ papillae in cells that retained the plasmid. A detailed summary of the results of the U3-Ty1*his3AI* screen and plate reference numbers can be found in Supplementary Table 2. Images of each plate showing growth and papillation for multiple transformants of each strain are provided in Supplementary Data Sheet 2.

Fifty-five mutants had wild-type levels of retromobility of the U3-Ty1*his3AI* element (Figure 1C). The 55 corresponding genes do not affect retromobility of a P_*TEF1*_- or U3-driven Ty1*his3AI* element on a *CEN*-plasmid in this strain background. These results do not necessarily contradict the results of earlier studies in which the genes were identified as regulators of Ty1*his3AI* retromobility; instead, they may indicate that these genes act in a context-dependent fashion. The critical context may not have been reproduced in our screen because the Ty1*his3AI* element is in a different chromatin environment or because of host strain differences. These host factors were classified as conditional regulators (CR) of retromobility.

A second major class of mutants identified by this screen contains 51 deletion mutants with a Ty1*his3AI* retromobility phenotype that differed from that of the wild-type strain (Figure 1C). The 51 corresponding genes regulate retromobility of a *CEN*-based, U3-driven but not P_*TEF1*_-driven Ty1*his3AI* element. This set of U3-Dependent Regulators, or U3DRs, contains 23 RTTs and 28 RHFs including Snf2, a component of the SWI/SNF complex that is required for Ty1 RNA transcription (Ciriacy et al., 1991; Happel et al., 1991). Rtt101, which restricts the mobility of a U3-driven but not P_*GAL1*_-driven Ty1*his3AI* element (Curcio et al., 2007), was also found in this class, as predicted. These findings validate the screening method used to identify U3DRs. Both components of the nuclear exosome that were in the cohort of 188 Ty1 regulators, Rrp6 and Lrp1, were indentified as U3DRs.

While all 51 *u3dr* mutants were previously found to affect Ty1*his3AI* retromobility, six of the 51 (12%) had the opposite effect on *CEN*-Ty1*his3AI* retromobility as that reported previously (Sundararajan et al., 2003; Nyswaner et al., 2008; Dakshinamurthy et al., 2010). Deletion of *ARG4*, *CKB2*, *RAD27* or *YLR282C* resulted in hypermobility of a chromosomal Ty1*his3AI* element in previous studies, but had decreased retromobility of the *CEN*-Ty1*his3AI* element; similarly, *pap2∆* and *ssk1∆* had reduced levels of chromosomal Ty1*his3AI* retromobility in a previous study but had substantially increased retromobility of the *CEN*- Ty1*his3AI* element (Supplementary Table 2; Supplementary Data Sheet 2). This unexpected finding underscores the influence of the context of the Ty1*his3AI* element on the retromobility phenotype of certain mutants.

### Gene Ontology and Growth Rates

**FIGURE 2:**
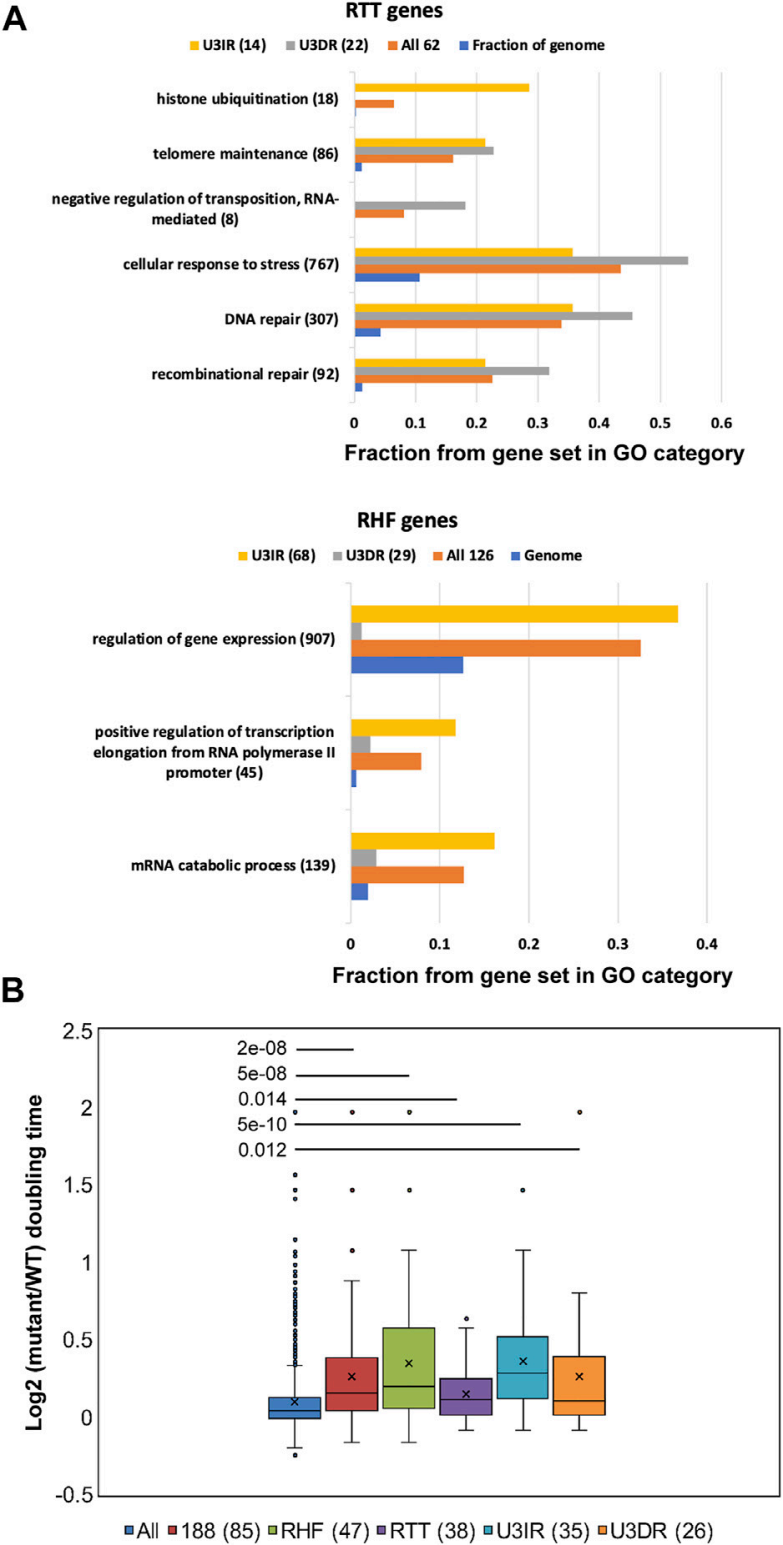
*RHF* and *RTT* genes are enriched for diverse GO categories and for genes affecting growth rate. **(A)** Representative GO process categories enriched among *RHF* and *RTT* genes and their U3IR and U3DR subsets are depicted. The fraction of genes from each category that are found in each subset, and in the entire genome, is shown. Only GO categories with <1,000 entries and corrected *p*-values of <10^−5^ are shown, and highly redundant categories were omitted. Complete listing of all enriched GO process categories with *p* < 0.01 can be found in Supplementary Table 3. **(B)** Box and whisker plots showing the log_2_ of the relative doubling time, compared to wild type, of all 1,312 mutants (“All”) [from O'Duibhir et al. (2014)] and indicated subsets. Average values in each plot are depicted by an X and the median by the solid line; outliers are shown as individual points. The number of genes for which growth was measured in deletion mutants is shown in parenthesis for the various categories of genes affecting Ty1 mobility. *p*-values for each cohort compared to the complete set of 1,312 mutants for which doubling times were measured were calculated using the Mann-Whitney *U*-test.

To determine whether the subset of 82 U3IR genes differs functionally from the subset of 51 U3DR genes, we performed Gene Ontology (GO) analysis. We further divided the genes in these two categories, and in the starting cohort of 188 genes, into *RTT* and *RHF* genes for this analysis. The complete set of enriched GO categories having *p* < 0.01 after correcting for multiple category testing is contained in Supplementary Table 3. Many categories contain highly overlapping gene sets; representative enrichments are depicted in Figure 2A, where we focused on categories having *p* < 10^−5^ for at least one gene set, and including fewer than 1,000 genes. As expected, distinct GO enrichments were observed for *RTT* and *RHF* genes. *RTT* genes were enriched for genes in the related categories of DNA repair and recombination, telomere maintenance, and response to stress (Figure 2A; Supplementary Table 3). Enrichment in these categories was also seen for both U3DR and U3IR cohorts, suggesting that distinct genes within these categories can affect transposition in both promoter-dependent and independent manners. Four of the eight genes in the GO category of Negative Regulators of Transposition (GO:0010529) were among the 22 *U3DR*-*RTT* genes. The enriched category of histone ubiquitylation seen for *U3IR-RTT* genes includes two members of the *CDC73-PAF1* complex, *CDC73* and *LEO1*. Other members of this complex were not tested. The 29 *U3DR-RHF* genes showed no enriched categories using our criteria, while the 68 *U3IR-RHF* genes were enriched for genes involved in mRNA catabolism, gene expression, and transcriptional elongation, the latter including four members of the CCR4-Not complex out of five that were tested (Figure 2; Supplementary Table 3).

A previous study identified a cohort of 1312 *S. cerevisiae* genes contributing to gene expression changes in a large number of transcriptome studies; deletion strains corresponding to these genes share the property of slow growth and have a common gene expression signature that was primarily attributed to a redistribution of cells over the cell cycle, with a greater fraction of cells in G1 (O'Duibhir et al., 2014). *RHF* and U3IR genes (which are mostly *RHF* genes) are enriched for this slow growth gene set and show substantial effects on growth when mutated, while *RTT* and U3DR genes exhibit only modest effects (Figure 2B). Ty1 retrotransposition is thought to be cell-cycle dependent (Xu and Boeke, 1991; Curcio et al., 2007; Curcio et al., 2015); this result suggests that many of the mutations identified as causing decreased Ty1 mobility do so in part by extending the G1 phase of the cell cycle. However, it should be emphasized that this is almost certainly only a partial effect, as many mutants that result in longer doubling times do not alter Ty1 mobility.

### Ty1 RNA and Ty1i RNA Levels in *u3dr* Mutants

Host factors that require the U3 promoter for their effects on retromobility might be expected to regulate Ty1 or Ty1i RNA levels. In mutants lacking one of the U3-dependent *RHF* genes *MED3* or *MED15*, Ty1 gRNA levels were similar to that in the wild-type strain, but Ty1i RNA levels were increased, resulting in elevated levels of the p22-Gag restriction factor (Salinero et al., 2018). We screened other *u3dr-rhf* mutants identified here for a substantially lower level of Ty1 gRNA or detectable level of Ty1i RNA that could result in a hypomobility defect. A northern blot of oligo-dT purified RNA from 21 *u3dr-rhf∆*, two *u3ir-rhf∆* and four CR/*rhf∆* mutants was performed using a riboprobe that hybridizes to both Ty1 RNA and Ty1i RNA, and the levels of Ty1 gRNA relative to a control transcript, *PYK1* mRNA, were measured. (Supplementary Presentation 2). Compared to the relative Ty1 gRNA level in the wild-type strain BY4741, none of these 27 *rhf∆* mutants had Ty1 gRNA levels that were decreased ≥2-fold except for the *CR/rhf∆* mutant, *vps34∆*, which had a 20-fold decrease in Ty1 gRNA. Thus, a substantial decrease in Ty1 gRNA is not likely to be the cause of the hypomobility phenotype of the 21 *u3dr-rhf∆* mutants that were screened. For the most part, we also failed to detect a conspicuous Ty1i RNA band in these mutants, except in the *rpl7a∆* mutant, which has previously been reported to have an elevated level of Ty1i RNA (Ahn et al., 2017). It is possible that diffuse signals below the Ty1 gRNA bands in the *rrp6∆*, *cst1∆* and *scj1∆* mutants are also indicative of increases in Ty1i RNA, but a more thorough analysis is necessary to determine whether this signal is due to smearing of the Ty1 gRNA band or increased Ty1i RNA. In summary, most of the U3-dependent and U3-independent *rhf* mutants tested did not have a conspicuous increase in Ty1i RNA that accompanied their decreased Ty1 retromobility.

Oligo-dT purified RNA from 16 *u3dr-rtt∆* mutants, four *u3ir-rtt∆* mutants and six CR/*rtt∆* mutants was also compared to that of the wild-type strain to determine whether increased Ty1 gRNA underlies the elevated retromobility in these mutants. Only the *u3dr-rtt* mutants *rtt109∆* and *mms1∆* had an increase of ≥2-fold (2.7-fold and 2-fold, respectively; Supplementary Presentation 2). A previous report saw no increase in total Ty1 gRNA in the *rtt109∆* mutant (Scholes et al., 2001); potentially, the difference between our results reflects the use of different strains or the use of total RNA earlier and polyA^+^ RNA here. The same study observed a 2-fold increase in steady-state Ty1 gRNA levels in the *mms1∆* (*rtt108∆*) mutant, in agreement with our results. We conclude that higher Ty1 RNA expression in the *mms1∆* and *rtt109∆* mutants may be an underlying cause of higher retromobility levels. The other 24 *rtt* mutants tested had only minor changes in Ty1 gRNA compared to the wild-type strain. Together, these findings indicate that most of the *rhf* and *rtt* mutants tested do not have substantially altered levels of Ty1 gRNA that are consistent with their retromobility defect; thus, some U3 promoter-dependent host factors may act at a post-transcriptional step in retromobility or affect expression of Ty1i RNA. While major increases in Ty1i RNA were not observed in this single northern, resolving Ty1i RNA presents a challenge, and it may be that subtle changes in Ty1i RNA levels can have more pronounced effects on retromobility. Furthermore, Ty1i RNA was not detected in the wild-type strain, so northern blotting could not be used to determine whether a decrease in Ty1i RNA accompanies the increase in retromobility in *u3dr-rtt* mutants. These challenges prompted us to develop a sensitive assay to measure the relative level of Ty1i RNA product, p22-Gag, in *u3dr-rtt* and other mutants.

### p22-Gag Expression in *u3dr* Mutants

**FIGURE 3:**
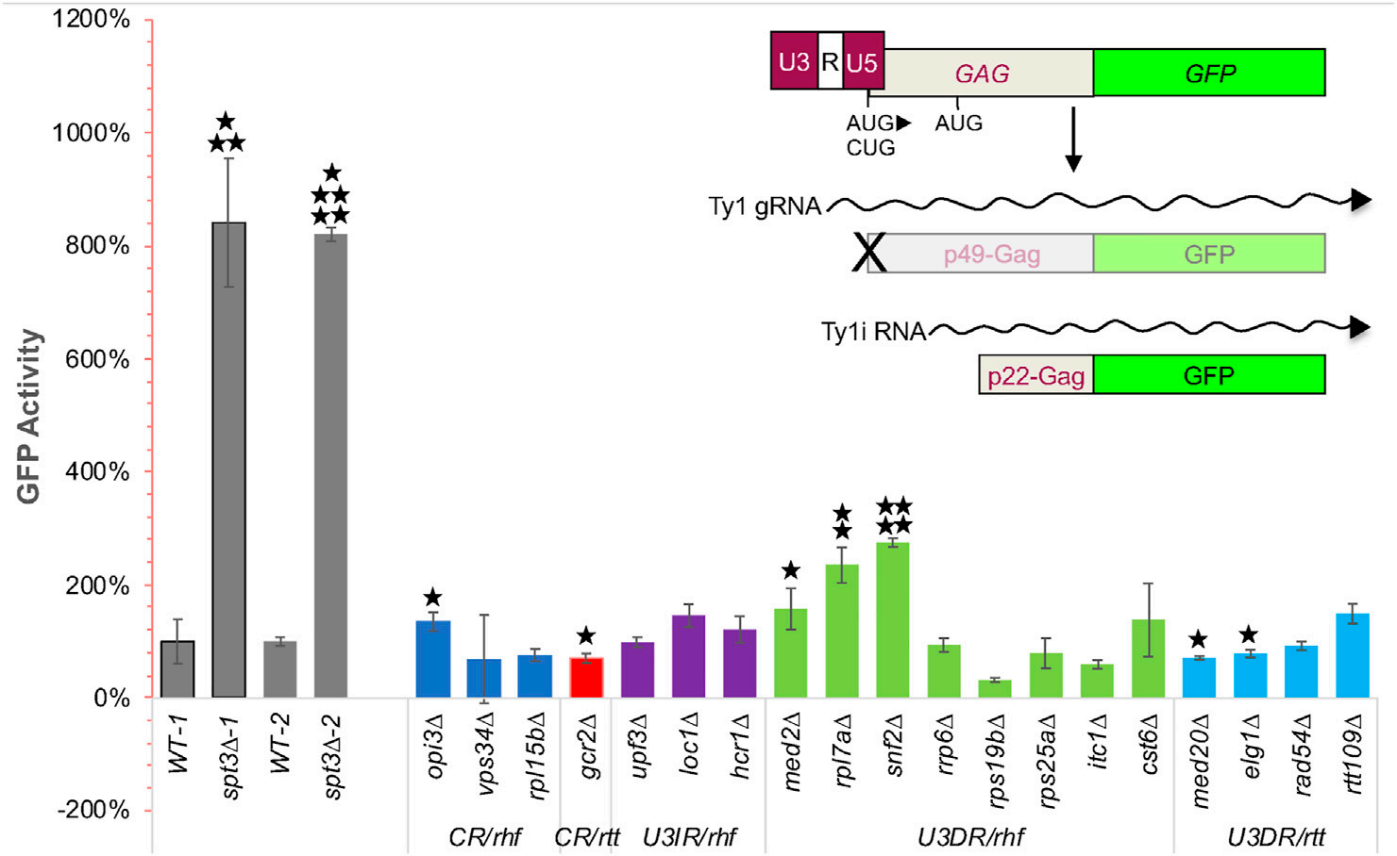
A *CEN*-plasmid based p22-Gag:GFP expression assay to detect relative increases and decreases in GFP activity in *rhf* and *rtt* mutants, respectively, identifies mutants with altered p22-Gag expression that corresponds to their retromobility defect. Schematic on upper right shows the LTR-GAG:GFP cassette in plasmid pBJC1270 that was used to measure p22-Gag:GFP expression; p45-Gag:GFP (indicated by the “X”) is not expressed because the first AUG codon in the *GAG* ORF is mutated. The transcript from the internal Ty1i promoter is not affected by this mutation and expresses p22-Gag:GFP. In the graph, GFP activity is the average of the geometric mean of GFP signal in 10,000 cells of each of three independent transformants of plasmid pBJC1270 corrected for autofluorescence by subtracting the average of the geometric means of GFP signal in 10,000 cells of three independent transformants of the vector in the same strain. WT-1 and WT-2 are two independent measurements of GFP activity in wild-type strain BY4741 using independent transformants that were performed in parallel with one of two independent measurements of GFP activity in the *spt3∆* derivative of BY4741 (*spt3∆-1* and *spt3∆-2*, repectively). Four other independent measurements of GFP activity in the wild-type strain performed in parallel with other mutants are not shown. *p*-values for the GFP activity of each mutant relative to the GFP activity in the wild-type strain analyzed in parallel were determined using a one-tailed Student’s *t*-test. Only increased GFP activity in *rhf* mutants or decreased GFP activity in *rtt* mutants was deemed of interest. *, *p* < 0.05; **, *p* < 0.01, ***, *p* < 0.001; ****, *p* < 0.0001; *****, *p* < 0.000001.

To compare levels of the self-encoded Ty1 restriction factor among hypermobile *rtt* and hypomobile *rhf* mutants, we designed a *CEN*-plasmid reporter construct that expresses p22-Gag:GFP from the Ty1i promoter (Figure 3). Since the Ty1i promoter has not been completely delineated and may overlap regions of the Ty1 promoter, the plasmid included the entire 5′ LTR and most of the *GAG* ORF, up to the C-terminal protease cleavage site, where *GFP* was fused (Doh et al., 2014). A single A to C nucleotide substitution was introduced in the most upstream start codon within *GAG*, such that p49-Gag:GFP can no longer be translated from Ty1 gRNA, and protein expression from a downstream in-frame AUG at nucleotide 321 is obstructed by four out-of-frame AUG codons between CUG_1_ and AUG_321_ (Dvir et al., 2013). However, p22-Gag is not expressed from Ty1 gRNA, and the nucleotide substitution is not present in Ty1i RNA, so p22-Gag:GFP expression should not be affected by this nucleotide substitution (Figure 3). The p22-Gag:GFP expression vector was introduced into strain BY4741 and mutant derivatives, and the relative level of GFP activity in 10,000 individual cells of each of three biological replicates was measured by flow cytometry.

To determine whether this assay yields results that are comparable to those in earlier studies, we compared p22-Gag:GFP expression in the wild-type strain to that of the *spt3∆* mutant, which has little Ty1 gRNA but increased levels of Ty1i RNA and p22-Gag (Saha et al., 2015). In two individual experiments, deletion of *SPT3* resulted in a 7- or 10-fold increase in the p22-Gag:GFP level (*p* < 0.001 to *p* < 0.000001, Student’s *t*-test; Figure 3). Retromobility mutants, including control strains lacking Mediator subunits Med2 and Med20, were then compared to the wild-type strain assayed in parallel in one of six separate experiments. In the hypomobile *med2∆* mutant, p22-Gag:GFP was increased 1.57-fold (*p* < 0.05; Student’s *t*-test), in agreement with previous findings of elevated Ty1i RNA and p22-Gag (Salinero et al., 2018). In contrast, p22-Gag:GFP activity in the hypermobile *med20∆* strain decreased to 71% of that in the wild-type strain (*p* < 0.05, Student’s *t*-test; note that because GFP activity of mutants was compared to that of wild type assayed in parallel and not all mutants were assayed together, significance is not necessarily apparent from visual inspection of expression level and standard deviation). This reduction in p22-Gag:GFP activity is consistent with the hypermobility of a *med20∆* strain but decreased expression of Ty1i RNA or p22-Gag was not detectable by northern or western blot analysis (Salinero et al., 2018). Together, these findings support the use of this assay to monitor p22-Gag expression, and suggest that the assay provides greater sensitivity in detecting decreased Ty1i or p22-Gag expression than northern or western blot analysis.

The p22-Gag:GFP activity in three *u3dr-rtt* mutants was measured. The *elg1∆* mutant had reduced GFP activity (*p* < 0.05, Student’s *t*-test), but *rtt109∆* and *rad54∆* did not. Of seven *u3dr-rhf* mutants tested, only *rpl7a∆* and *snf2∆* had significant increases in p22-Gag:GFP levels consistent with their hypomobility phenotypes (*p* < 0.01 and *p* < 0.00001, respectively; Student’s *t*-test). An elevated level of p22-Gag in the *rpl7a∆* mutant parallels the elevated level of Ty1i RNA observed by northern blot analysis (Supplementary Presentation 2). The other five *u3dr-rhf* mutants, *rrp6∆*, *rps19b∆*, *rps25a∆*, *itc1∆* and *cst6∆* did not have significantly elevated levels of GFP activity (Figure 3), consistent with the absence of a discrete Ty1i RNA band in northern blot analysis (Supplementary Presentation 2). Together these findings reveal that a host factor’s dependence on the U3 promoter is not necessarily predictive of it modulating the level of Ty1i RNA or p22-Gag.

Other classes of mutants were examined for comparison. Three U3-independent *rhf* mutations, *upf3∆*, *loc1∆* and *hcr1∆* had little effect on p22-Gag:GFP levels. Notably, two mutants that had a wild-type Ty1*his3AI*-CEN retromobility phenotype did show significant changes in p22-Gag:GFP activity. The *gcr2∆* mutant, which has been reported to have a hypermobility phenotype (Nyswaner et al., 2008), had a reduced amount of p22-Gag:GFP activity (*p* < 0.05), while *opi3∆*, shown previously to have hypomobile phenotype (Dakshinamurthy et al., 2010), had more GFP activity than the wild-type strain (*p* < 0.05) (Figure 3). These changes in the amount of p22-Gag:GFP were detected in the absence of any detectable change in Ty1i RNA (Supplementary Presentation 2). Since Gcr2 and Opi3 modulate Ty1 retromobility under certain conditions, they may do so by controlling p22-Gag expression. Overall though, our findings suggest that some U3-dependent regulators, notably Elg1, Rpl7a and Snf2, can modulate the level of Ty1i RNA or p22-Gag in a manner consistent with their retromobility phenotype, while others may regulate retromobility by mechanisms that do not directly impact expression from the Ty1 or Ty1i promoter. The latter possibility is explored below.

### Promoter Activity or Architecture Govern the Dependence of Host Factors on U3

The U3-dependence of some modulators of Ty1 retromobility could be related to U3 being a weak promoter. A co-factor could improve the translational efficiency of Ty1 gRNA translation when U3 drives Ty1 expression but be dispensable when higher levels of Ty1 RNA are expressed from a stronger promoter. Another possibility is that host factors exhibit dependence on U3 because of its architecture. U3DR host factors could be directed via an interaction with the U3 promoter to associate with Ty1 RNA and control the modification, export, localization or translation of Ty1 RNA or Ty1i RNA (Zid and O'Shea, 2014; Espinar et al., 2018). We asked whether U3DR genes fall into one or both of these functional categories by testing retromobility of a Ty1 element driven by the P_*PSP2*_ promoter in the corresponding mutants (Figure 1A). P_*PSP2*_ is a weak promoter, like U3 (Supplementary Presentation 1), but is TATA-less and Taf1-enriched (Rhee and Pugh, 2012), and is insensitive to rapid depletion of SAGA (Donczew et al., 2020), indicating that it is more similar in architecture to P_*TEF1*_ than to the SAGA-dominated U3 promoter. A *u3dr* mutation that does not affect P_*PSP2*_-driven Ty1*his3AI* retromobility by definition also does not affect P_*TEF1*_-driven Ty1*his3AI* retromobility and therefore is likely to depend primarily on the architecture of U3 rather than the level of expression. On the other hand, any *u3dr* mutation that also affects P_*PSP2*_-driven Ty1*his3AI* retromobility may exert its effect in a way that depends on promoter strength and not architecture, or may reflect an architectural effect that is suppressed when Ty1*his3AI* is driven by a strong promoter that is not affected by the specific *u3dr* mutation.

**FIGURE 4:**
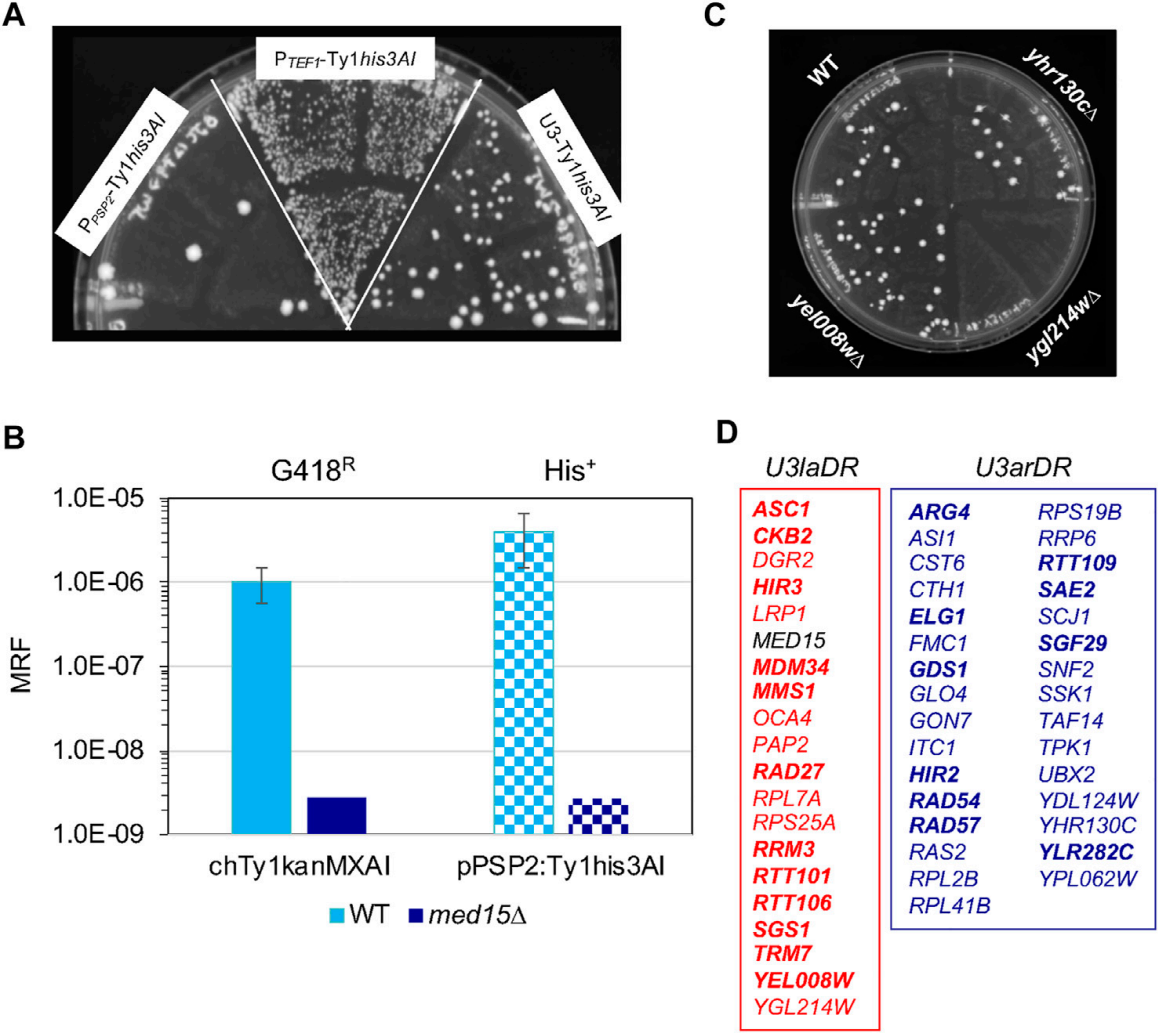
Identification of host *rhf* and *rtt* mutants that are dependent on the low activity or architecture of the U3 promoter. **(A)** A semi-quantitative assay comparing retromobility of Ty1*his3AI* on the identical *CEN*-plasmid and driven from the P_*TEF1*_, U3 or P_*PSP2*_ promoter. His^+^ Leu^+^ papillation (indicative of Ty1*his3AI* retromobility) of three independent transformants of the wild-type strain carrying the *LEU2*-marked *CEN*-plasmid with P_*PSP2*_-Ty1*his3AI*, P_*TEF1*_-Ty1*his3AI* or U3-Ty1*his3AI*, as indicated, following growth on YPD agar at 20°C to induce retrotransposition. **(B)** Graph showing the mean retromobility frequency (MRF) in six independent transformants of the *CEN*-P_*PSP2*_-Ty1*his3AI* plasmid in the wild-type (light blue bars) or congenic *med15∆::URA3* (dark blue bars) strain bearing a chromosomal Ty1*kanMXAI* element. Solid bars- frequency of G418^R^ reversion (indicative of Ty1*kanMXAI* retromobility); checked bars-frequency of His^+^ reversion (indicative of Ty1*his3AI* retromobility). Error bars; standard error. The absence of error bars on dark blue or dark-blue-checked bars indicates that the value is an estimated maximum retromobility frequency because zero G418^R^ or His^+^ colonies were observed in cultures of six independent transformants of the *med15∆* strain. **(C)** A SC-Leu-His plate from the P_*PSP2*_-Ty1*his3AI* screen showing relative levels of His^+^ Leu^+^ papillation (indicative of Ty1*his3AI* retromobility) in transformants of the wild-type strain, *yhr130C∆* (an *rhf* mutant with no change in His^+^ Leu^+^ papillation relative to the wild-type strain), *ygl214w∆* (an *rhf* mutant with decreased His^+^ Leu^+^ papillation) and *yel008w∆* (an *rtt* mutant with increased His^+^ Leu^+^ papillation) mutants. **(D)** Lists of U3-low activity dependent regulators (*U3laDR*) in red and U3-architecture dependent regulators (*U3arDR*) in blue, as identified by the P_*PSP2*_-Ty1*his3AI* screen. Bold type indicates *RTT* genes; regular type indicates *RHF* genes. *MED15*, indicated in black, is a control.

The relative strength of U3, P_*TEF1*_, and P_*PSP2*_ in driving retromobility in the wild-type strain was determined using the identical *CEN*-vector and Ty1*his3AI* sequence. P_*PSP2*_ had the weakest activity, followed by U3 and then P_*TEF1*_ (Figure 4A). Thus, if the dependence of retromobility on a host factor requires the weak strength of the U3 promoter but is independent of its architecture, that factor should also regulate P_*PSP2*_ *-*Ty1*his3AI.* Our labs have previously shown that *med15∆* abolishes retromobility of a chromosomal Ty1*kanMXAI* element but has only a minor effect on retromobility of a plasmid-based P_*TEF1*_-Ty1*his3AI* in the same strain (Salinero et al., 2018). In a parallel experiment, we compared the effect of deleting *MED15* on the same chromosomal U3-driven Ty1*kanMXAI* element and the plasmid-based P_*PSP2*_-Ty1*his3AI* element transformed into the same strain (Figure 4B). In the absence of *MED15*, retromobility of both elements was abolished. These findings indicate that suppression of the reduced Ty1 retromobility of the *med15∆* mutant seen when Ty1*his3AI* is driven by the P_*TEF1*_ promoter requires the strong promoter activity of the *TEF1* promoter in addition to any possible contribution of promoter architecture.

P_*PSP2*_-Ty1*his3AI-CEN* was introduced into the 51 *u3dr* mutants, and the retromobility patch assay was performed as it was for P_*TEF1*_-Ty1*his3AI* (Figure 1B). The relative number of His^+^ Leu^+^ prototrophs formed in patches of multiple transformants of each mutant was compared to that of the congenic wild-type strain, grown on the same plate (Figure 4C; Supplementary Data Sheet 3). Mutants with an elevated or reduced number of His^+^ Leu^+^ papillae per patch such as *ygl214w∆* and *yel008w∆* (Figure 4C), respectively, control Ty1*his3AI* retromobility when transcription was driven from the *PSP2* promoter. Like Med15, these host co-factors and restriction factors may depend primarily on Ty1 gRNA being driven from a weak promoter for their effect on retromobility, and thus are designated as U3 low activity-dependent regulators (U3laDRs). Nineteen *U3laDR* genes were identified, 12 (63%) of which encode Ty1 repressors (Figure 4D, red box, *RTT* genes in bold). Another 31 mutants, including *yhr130c∆* (Figure 4C), had a similar level of His^+^ papillation as the wild-type strain (Figure 4D, blue box), suggesting that the architecture of U3 is more critical than its low activity for their function. The phenotype of one mutant, *caf130∆*, could not be determined due to a growth defect (Plate 10, Supplementary Data Sheet 3). Genes that didn’t modulate P_*PSP2*_-driven retromobility were classified as U3 architecture-dependent regulators (U3arDRs). Most U3arDRs (21/31) were Ty1 retromobility co-factors. Snf2, a component of the SWI/SNF complex that is required for U3 but not Ty1i promoter activity, was identified as a U3 architecture-dependent regulator, as predicted. Other U3arDR transcription-related proteins include the SAGA and TFIID component, Taf14; SAGA component, Sgf29; histone gene transcription factors, Hir2 and Hir3; and the putative transcription factor Cth1.

## Discussion

A major impediment to understanding how host cells control the activity of retroviruses and retrovirus-like transposons is the multitude of host genes that influence retroelement replication at different stages and in different cellular compartments. Genetic screens for retroelement host factors often yield large non-overlapping gene sets (Maxwell and Curcio, 2007a; Goff, 2008; Zhou et al., 2008), suggesting that the function of many host factors depends on the individual element assayed, method and level of expression, the genotype of the host, or environmental variables. Here, we explore the hypothesis that many Ty1 host factors act conditionally by focusing on differences in their dependence on the Ty1 promoter. Several previous studies have suggested that the level of Ty1 RNA could impact the function of certain Ty1 co-factors and restriction factors, but this is the first study to take a systematic approach to exploring the dependence of Ty1 retromobility modulators on the U3 promoter and to examine the specific features of U3 involved in regulation.

We used serial retromobility screens of different plasmid-based Ty1*his3AI* elements to classify host genes encoding modulators of Ty1 retrotransposition based on whether or not they retain their effects on retromobility when a promoter with higher activity and different architecture drives the expression of Ty1 gRNA. Analysis of 181 previously identified and seven newly identified regulators of Ty1 retromobility revealed two major classes. The largest class comprises 82 U3-independent regulators that repress or stimulate retromobility of a Ty1*his3AI* element even when it is expressed from P_*TEF1*_, a strong, TATA-less, TFIID-dominated promoter. The second class of 51 U3-dependent regulators modulates the activity of a plasmid-based Ty1*his3AI* element when it is expressed from the TATA containing, SAGA-dependent U3 promoter but not when expressed from P_*TEF1*_. Of these U3DRs, 19 regulate the mobility of Ty1*his3AI* with the weak, TATA-less, TFIID-dominated *PSP2* promoter, whereas 31 did not affect P_*PSP2*_-Ty1*his3AI* retromobility. Characterization of these subclasses, U3laDRs and U3arDRs, respectively, suggests that there are multiple genetic pathways that modulate Ty1 retromobility even within the categories of U3 low activity-dependent and U3 architecture-dependent promoters (Figure 4).

Of the 188 *rhf* and *rtt* mutations that we analyzed, 55 (29%) had no detectable effect on retromobility of the *CEN*-Ty1*his3AI* element (CR/*rtt* and CR/*rhf* mutants; Figure 1C; Supplementary Table 1), despite the corresponding genes having been identified in one or more earlier genetic screens as modulators of Ty1*his3AI* mobility. Some members of this group may have been false positives in earlier screens; however, we favor the idea that most of these genes regulate Ty1 retromobility in a conditional manner. The discrepancy between our results and earlier studies could be due to a difference between our retromobility assay, which employs a *CEN*-based Ty1*his3AI* element, and that of previous genetic screens, which employed a Ty1*his3AI* element on an integrating plasmid in the host genome or introduced into the host genome by transposition (Scholes et al., 2001; Nyswaner et al., 2008; Dakshinamurthy et al., 2010; Risler et al., 2012). We previously observed retromobility of an LTR-driven Ty1*his3AI* element to be >30-fold higher when expressed from a *CEN*-plasmid than from a chromosomal site, with concomitant partial suppression of the elevated retromobility seen for deletion of Mediator head and middle subunits (Salinero et al., 2018). An example of a CR/*rtt* mutant is *mre11∆*, which has a wild-type *CEN*-Ty1*his3AI* retromobility phenotype (Supplementary Table 1). The *mre11∆* mutant has previously been shown to have elevated retromobility of a chromosomal Ty1*his3AI* element and higher levels of total cellular Ty1 cDNA in two different strain backgrounds (Scholes et al., 2001; Curcio et al., 2007). Moreover, there is increased retromobility of Ty1*his3AI* on a plasmid integrated into the genome in the *mre11∆* mutant (Nyswaner et al., 2008). Perhaps higher expression of Ty1*his3AI* when it is located on a *CEN*-plasmid versus in the chromosome overcomes the restriction activity of Mre11 (Salinero et al., 2018).

A few mutants in the category of potentially conditional regulators also had specific changes in Ty1 retromobility intermediates that were consistent with their previously reported phenotypes, arguing against the idea that they are false positives. Three *rhf* mutants that fall in this category, *opi3∆*, *elo1∆* and *vps34∆* had decreased levels of polyA^+^ Ty1 RNA in northern analysis (Supplementary Presentation 2). Two of four conditional mutants tested, the *rhf* mutant *opi3∆* and the *rtt* mutant *gcr2∆*, had an increase or decrease in p22-Gag:GFP levels, respectively, that was consistent with their reported retromobility phenotype (Figure 3). The chromatin context of Ty1*his3AI*, variations in plasmid copy number or some other factor related to the location of the Ty1*his3AI* on an episomal plasmid may lead to changes in the ratio of Ty1i:Ty1 RNA that influence the retromobility phenotype of mutants with deletions of CR genes.

The cohort of U3-independent Ty1 regulators identified by this work includes all five of the essential genes we tested using DAmP alleles: CCR-NOT complex components, *CDC36/NOT2* and *CDC39/NOT1*; *MSL5*, which encodes a splicing factor; *NDC1*, which encodes a subunit of the transmembrane ring of the nuclear pore complex; and *RPB7*, which encodes a subunit of the RNA Polymerase II core complex. *U3IR* genes are enriched for genes that when deleted, cause slow growth and typically, an elongated G1 phase of the cell cycle (O'Duibhir et al., 2014). This category of enrichment is also seen in the cohort of 126 *RHF* genes (Figure 2B). Thus, some of the effects on retromobility that have been observed in mutants corresponding to the *U3IR* cohort and/or *RHF* cohort may be an indirect result of an elongated G1 phase of the cell cycle. The idea that a prolonged G1 phase of the cell cycle inhibits Ty1 retromobility is also supported by the observation of a U3-independent decrease in Ty1 retrotransposition, cDNA and integrase in cells temporarily arrested in G1 by treatment with mating pheromone (Xu and Boeke, 1991).

Over 80% of U3-independent genes are *RHF* genes. The 68 *U3IR-RHF* genes include four of the five CCR4-NOT complex subunits that were analyzed. (Caf130 is a U3-dependent regulator). This cohort is enriched for genes involved in mRNA catabolism, gene expression, and transcriptional elongation. Notably, mRNA catabolism-related genes are one of the most highly enriched gene categories among Ty1 co-factors (Ahn et al., 2017). The 14 *U3IR-RTT* genes are enriched for genes involved in histone ubiquitylation, including two *CDC73-PAF1* complex components (Figure 2A). The opposing effects of genes in the *CDC73-PAF1* complex and in the CCR4-NOT complex raise the possibility that these complexes may exert counterbalancing effects on Ty1 retromobility that are promoter-independent.

The smaller group of U3-dependent genes has similar numbers of *RHF* and *RTT* genes and is not enriched for any GO categories that are not also enriched in the starting set of mutants. Enriched GO categories in both the U3-dependent *RTT* gene set and the entire *RTT* gene set were cellular response to stress, DNA repair, DNA recombination and telomere maintenance (Figure 2A). *U3DR* genes were also not enriched for genes that cause slow growth and extension of G1 when deleted (Figure 2B). Overall, our findings suggest that U3-dependent Ty1 regulatory genes may be more functionally diverse than U3-independent genes.

The *U3DR* gene set includes an unexpected set of six genes that influenced the retromobility of the *CEN*-Ty1*his3AI* element, but in the opposite direction to that reported previously; four known *RTT* genes had a hypomobility phenotype in the *CEN*-Ty1*his3AI* assay, whereas two known *RHF* mutants had a hypermobility phenotype (Supplementary Table 2). Mutations in all six genes failed to alter retromobility of P_*TEF1*_-Ty1*his3AI*, which is also located on a *CEN*-plasmid. Three genes were U3 architecture-dependent (*ARG4*, *YLR282C*, *SSK1*) and the other three were U3 low activity-dependent (*RAD27*, *CKB2* and *PAP2*). Of the latter three, only deletion of *RAD27* caused the opposite phenotype for P_*PSP2*_-Ty1*his3AI* retromobility to that anticipated based on previous findings (Plate 10, Supplementary Data Sheet 3) (Sundararajan et al., 2003). The molecular mechanisms underlying these conditional phenotypes are not understood, but they are likely varied and may influence Ty1 element stability, expression and retromobility. The role of one of these genes, *RAD27* in restricting retromobility lends some possible insight into one of these phenomena (Sundararajan et al., 2003). *RAD27* encodes a multifunctional exonuclease and flap endonuclease involved in DNA replication and repair and genome stability. In a *rad27* null mutant, spontaneous recombination is increased (Tishkoff et al., 1997; Debrauwère et al., 2001); moreover, Ty1 gRNA and Ty1*his3AI* gRNA levels are modestly elevated, Ty1 cDNA accumulates, cDNA multimers form, and both elevated Ty1 cDNA recombination and cDNA integration likely account for the observed increase in Ty1*his3AI* retromobility (Sundararajan et al., 2003). Thus, the conditional retromobility phenotypes of a *rad27∆* mutant may be related to changes in Ty1 cDNA levels or fate. One idea is that the relative amounts of Ty1*HIS3* cDNA produced from a *CEN*-based element, transposed chromosomal element and integrated plasmid-based element are different in the *rad27∆* mutant versus the congenic wild-type strain; such differences could be a reflection of varied expression levels of Ty1*his3AI* RNA from elements in these different contexts. A lower level of Ty1*HIS3* cDNA derived from *CEN*-Ty1*his3AI* and *CEN*-P_*PSP2*_-Ty1*his3AI* in the *rad27∆* mutant, possibly caused by loss of the Ty1*his3AI* element by recombination between LTRs or plasmid instability, could account for the apparent decrease in Ty1 retromobility. In the case of the *rad27∆* mutant carrying *CEN*-P_*TEF1*_-Ty1*his3AI,* an increase in Ty1*HIS3* cDNA resulting from higher expression of Ty1*his3AI* RNA might counteract DNA-based pathways of Ty1*his3AI* element loss, resulting in an apparent lack of change in the retromobility of *CEN*-P_*TEF1*_-Ty1*his3AI.*

The function of three-fourths of the U3-dependent host co-factors is dependent on the architecture of U3. As predicted, Snf2, which is required for U3-driven transcription of Ty1 gRNA (Happel et al., 1991), fell into the U3arDR-*RHF* class. However, none of the 16 other *u3ardr-rhf* mutants screened by northern analysis had a substantial decrease in Ty1 gRNA (Supplementary Presentation 2). This suggests that some U3-dependent co-factors may exert post-transcriptional effects. Precedence for such a scenario exists, as factors associated with transcriptional initiation and elongation can affect mRNA export and decay in a gene-or promoter-dependent fashion (Trcek and Singer, 2010; Haimovich et al., 2013; Fischl et al., 2017; Catala and Abou Elela, 2019). Nonetheless, the results of northern blot screen of steady-state levels of Ty1 gRNA should be interpreted with caution. Small changes in the level of Ty1 gRNA or relative level of Ty1*his3AI* RNA can lead to major differences in retromobility in some host mutants (Bonnet et al., 2021). Additional studies that probe U3 promoter occupancy, Ty1 RNA synthesis, and transcriptional activity of individual Ty1 elements would be necessary to confirm that individual co-factors do not affect U3 activity.

The unique relationship between Ty1 and Ty1i allows the possibility of an additional promoter-dependent mechanism that can affect retromobility. The promoters for Ty1 and Ty1i may be in competition, and mutants that affect the balance of this competition could affect retromobility without substantially altering Ty1 transcript levels. A similar competitive model was first suggested as a mechanism by which *spt* mutants could allow transcription of *his4* alleles that were disabled by insertion of the delta-912 element (an LTR) upstream of the *HIS4* proximal promoter (Hirschman et al., 1988). We previously found that loss of specific Mediator subunits altered Ty1 retromobility by altering the balance of Mediator and Pol II at the Ty1 and Ty1i promoters. The effect of these mutations on levels of Ty1 gRNA were minor but Ty1i RNA and p22-Gag levels were both increased in the absence of Mediator tail subunits (Salinero et al., 2018). However, none of the 16 *u3ardr-rhf* mutants tested had detectable Ty1i RNA, and only one of five tested (*snf2∆*) had an increased level of p22-Gag:GFP. Overall, the findings suggest that not all U3arDRs affect levels of the Ty1 restriction factor. Nonetheless, establishing whether the *u3ardr*-*rhf* mutants identified here operate by altering the ratio of Ty1i:Ty1 expression will require more in-depth studies.

Many ribosomal protein genes and ribosome biogenesis factors have been identified as RHFs, and they enhance retromobility by multiple mechanisms (Dakshinamurthy et al., 2010; Risler et al., 2012; Suresh et al., 2015; Ahn et al., 2017; Park et al., 2017). Thus, it was not surprising to find ribosomal protein genes scattered among U3-independent, U3-low activity dependent and U3 architecture-dependent categories. Rpl7a is a host factor whose absence causes a similar phenotype to the absence of Mediator tail subunit Med15. Both Rpl7a and Med15 function in a manner dependent on the weak activity of U3 (Figure 4D), and both *rpl7a∆* and *med15∆* have increased Ty1i RNA levels and p22-Gag levels (Figure 3; Supplementary Presentation 2) (Ahn et al., 2017; Salinero et al., 2018). Strikingly, Ty1 gRNA and Gag protein levels remain unchanged, but cDNA levels are low in both mutants (Risler et al., 2012; Ahn et al., 2017; Palumbo et al., 2017; Salinero et al., 2018). These phenotypes are consistent with changes expected as a result of increased p22-Gag, which inhibits VLP assembly and Ty1 RNA packaging (Saha et al., 2015; Ahn et al., 2017). Together, the data raise the possibility that *RPL7A* and *MED15* act in a common pathway to modulate retromobility. While Mediator subunits interact directly with both the Ty1 and Ty1i promoter, Rpl7a has not been reported to do so. It seems more likely that a stress pathway is induced in the absence of Rpl7a that impacts the relative expression of Ty1i, potentially via the Mediator complex. Notably, *loc1∆* was isolated in the same screen for genes that work in a copy-number specific manner as *rpl7a∆*; accordingly, both *LOC1* and *RPL7A* repress p22-Gag expression (Ahn et al., 2017). But in this work, *LOC1* was categorized as a U3-independent gene, and the p22-Gag:GFP activity was not increased in the *loc1∆* mutant. Loc1 may modulate p22-Gag levels by a different mechanism than Rpl7a; another possibility is that the p22-Gag:GFP assay used here is not as sensitive to elevated levels of p22-Gag as assays employed in other studies.

*S. cerevisiae* has co-opted Ty1 as a family of mostly autonomous LTR-retrotransposons (Curcio, 2019). Domestication of Ty1 benefits its host by ensuring cDNA production and genomic copies of Ty1 sequences associated with DNA fragile sites to repair chromosome breaks, extend chromosome ends in the absence of telomerase, and trigger potentially adaptive mutations and genome rearrangements in response to genomic and environmental stresses (Scholes et al., 2003; Maxwell et al., 2004; Maxwell and Curcio, 2007b; Gresham et al., 2008; Maxwell and Curcio, 2008; Chan and Kolodner, 2011; Maxwell et al., 2011; Curcio, 2019). Deleterious effects of retrotransposition are limited by targeting of integration events to gene poor regions of the genome and by copy number control, a process that involves extensive host factor-mediated regulation of the Ty1i-encoded restriction factor (Ahn et al., 2017; Salinero et al., 2018; Bonnet and Lesage, 2021). This systematic analysis illustrates how deeply enmeshed *S. cerevisiae* is in the regulation of Ty1 retrotransposition and cDNA-mediated recombination, potentially at different steps in the retromobility cycle, in a variety of subcellular domains and at different points of the cell cycle.

## Data Availability

The original contributions presented in the study are included in the article/Supplementary Material, further inquiries can be directed to the corresponding author.
