# Supplementary figures and images for: Reliance of Host-Encoded Regulators of Retromobility on Ty1 Promoter Activity or Architecture
Source: Front Mol Biosci. 2022 Jul 1;9:896215. doi: 10.3389/fmolb.2022.896215 (PMC9283973; doi:10.3389/fmolb.2022.896215)

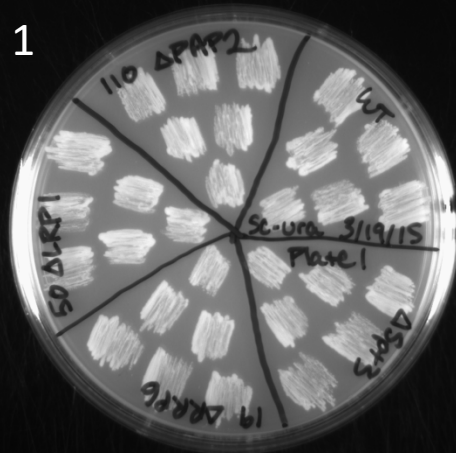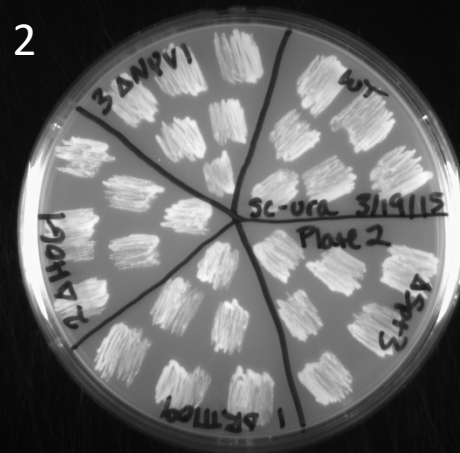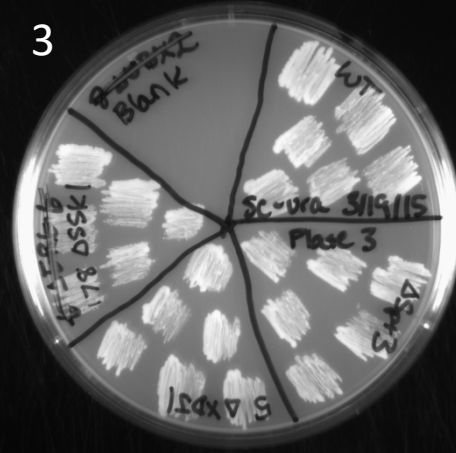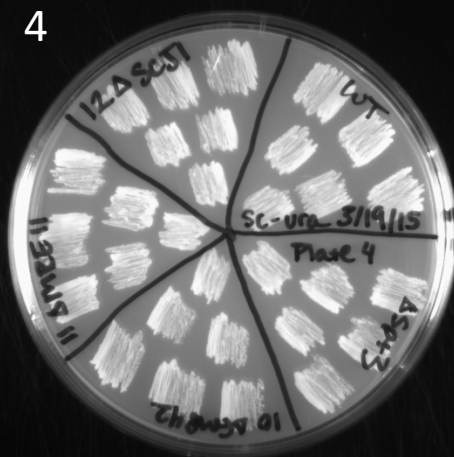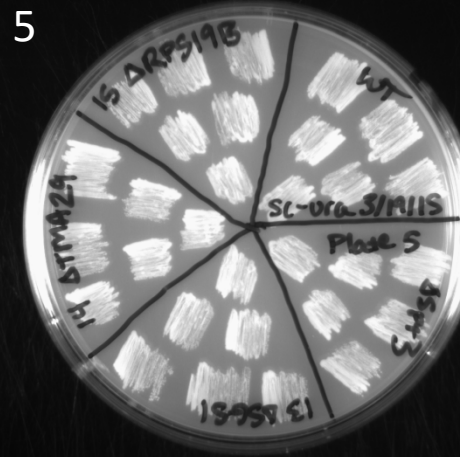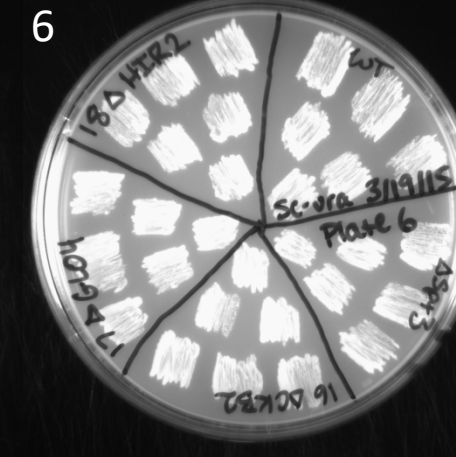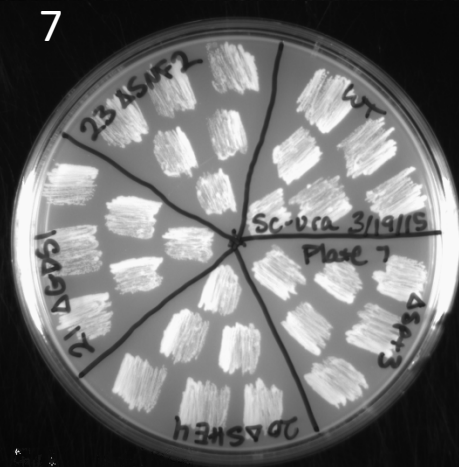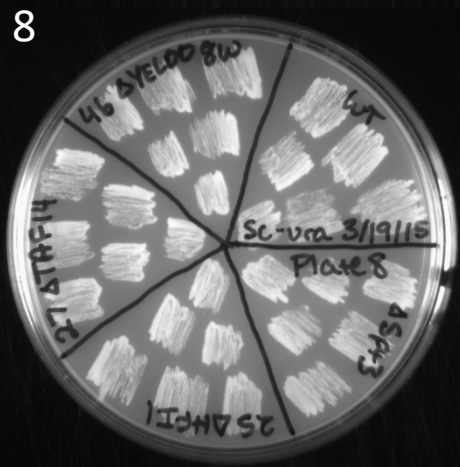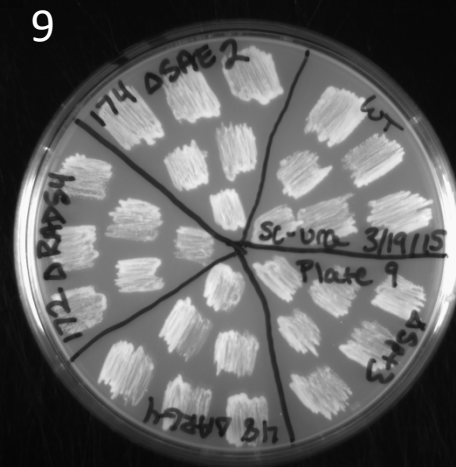

1

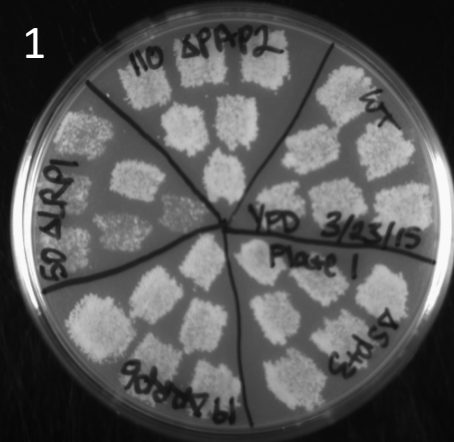

2

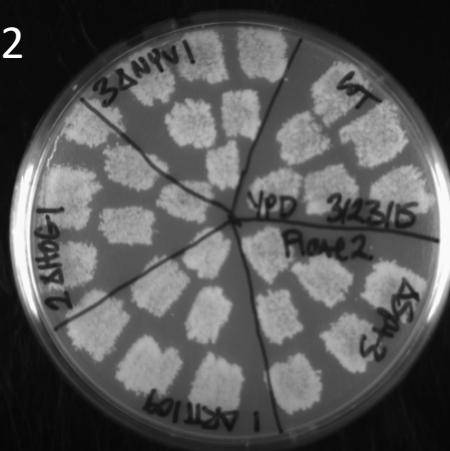

3

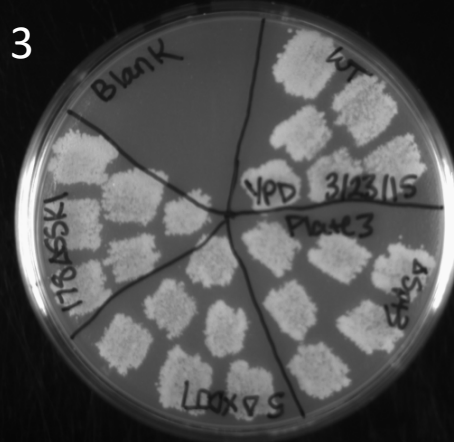

4

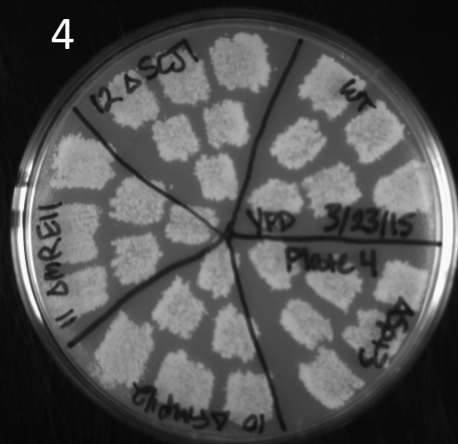

5

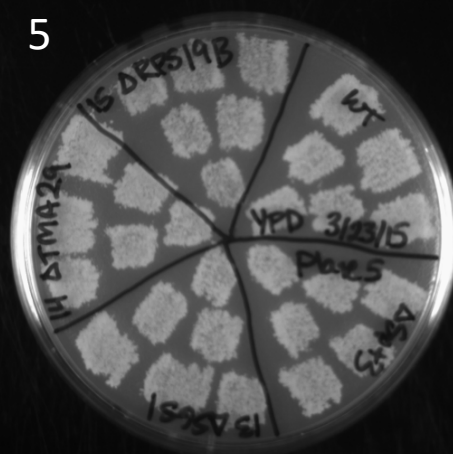

6

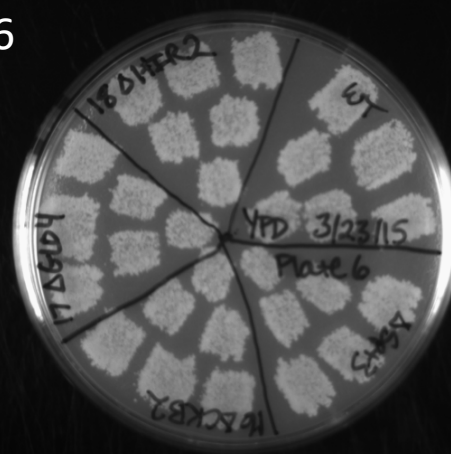

7

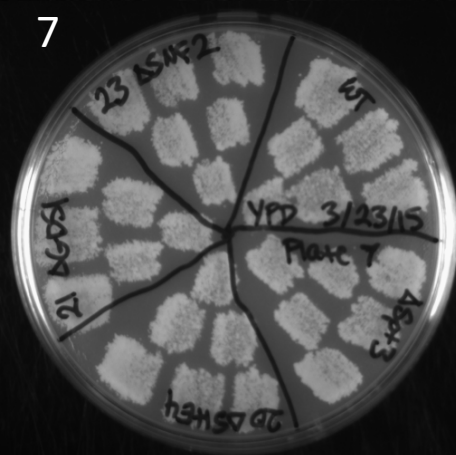

8

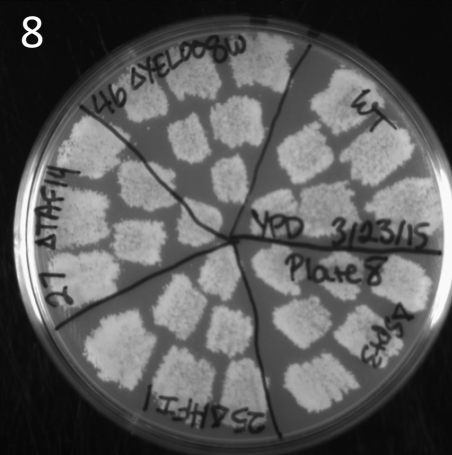

9

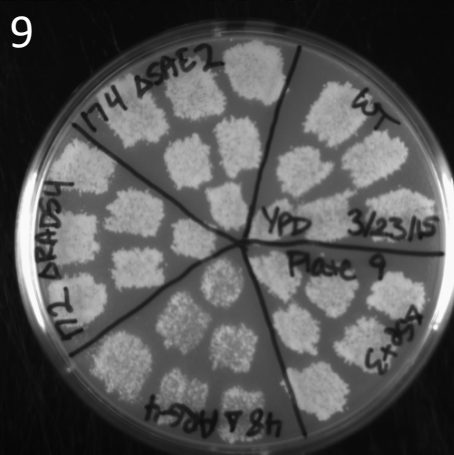

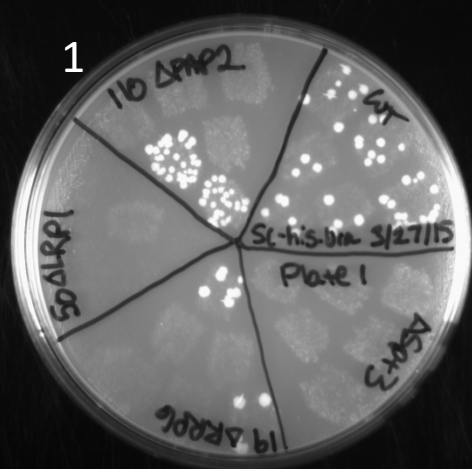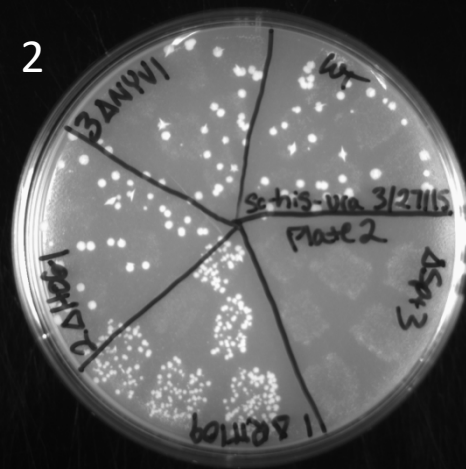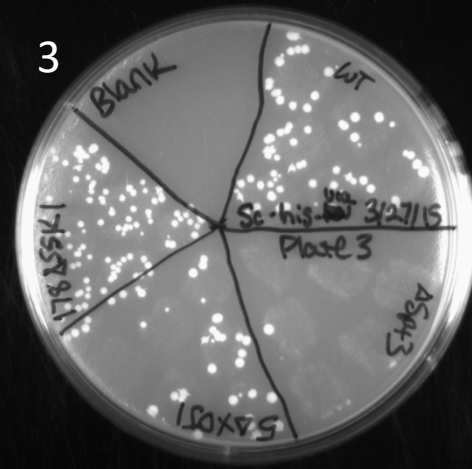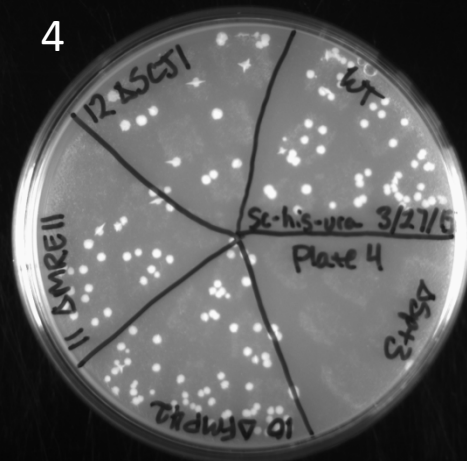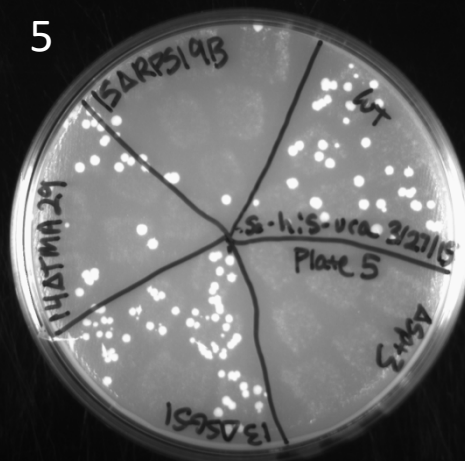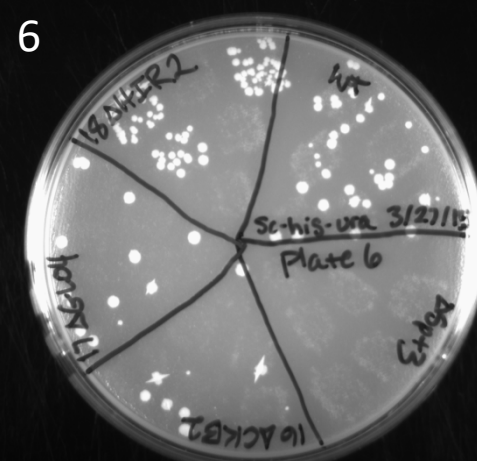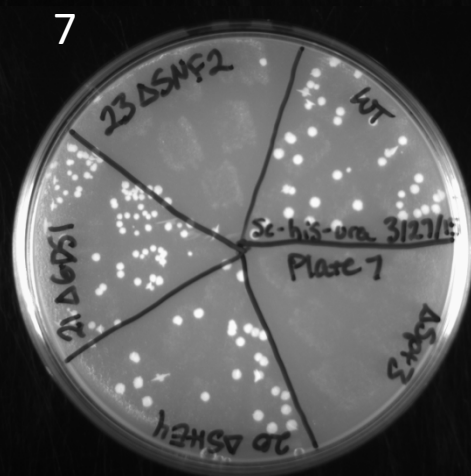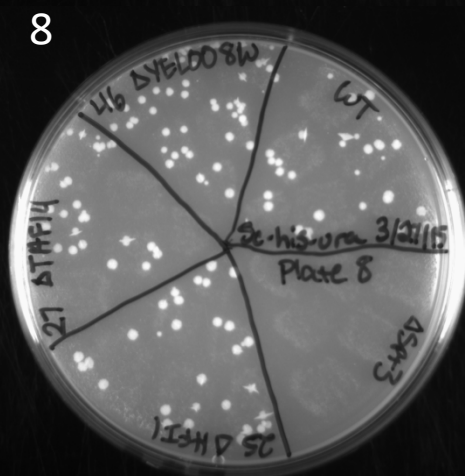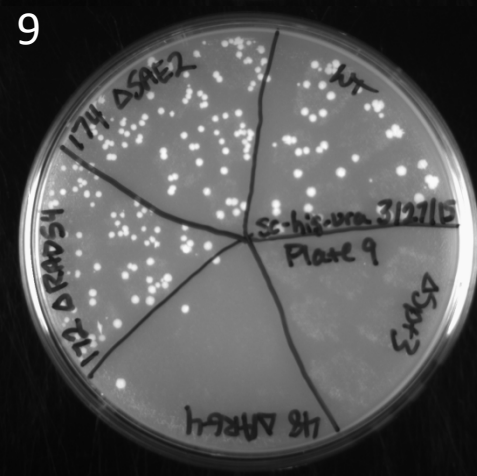

33

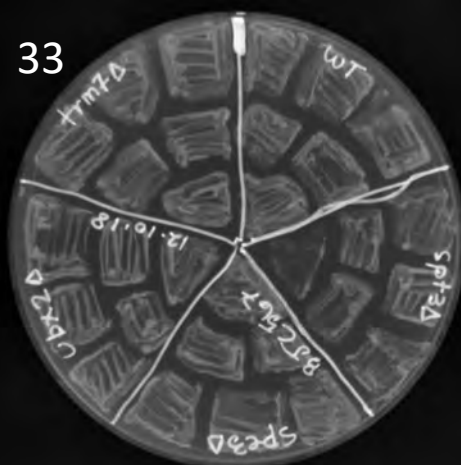

34

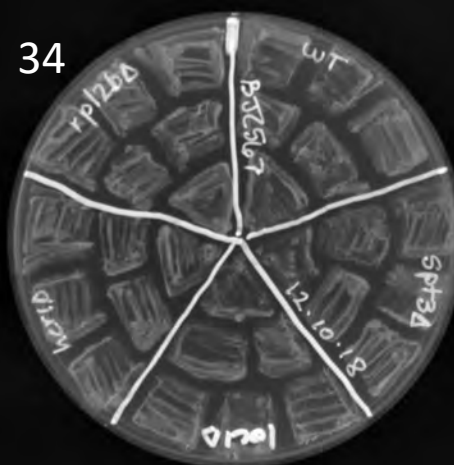

35

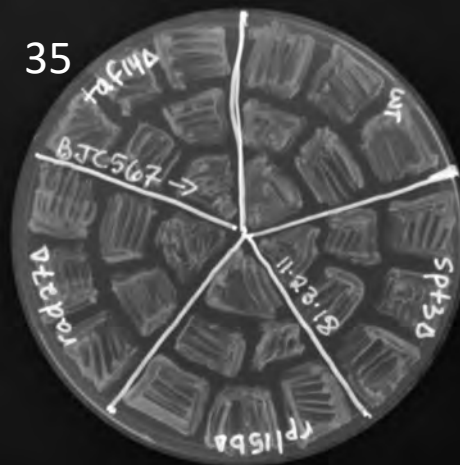

36

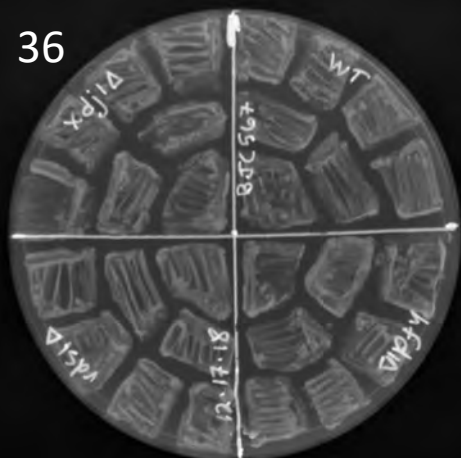

37

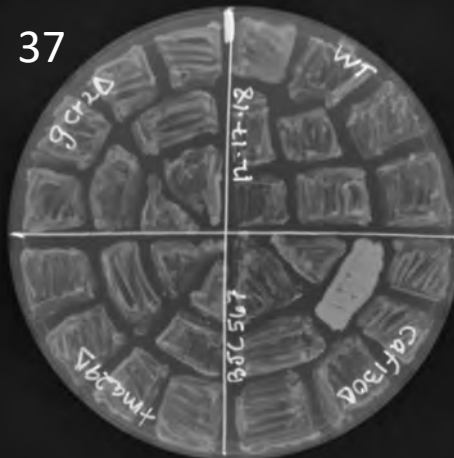

38

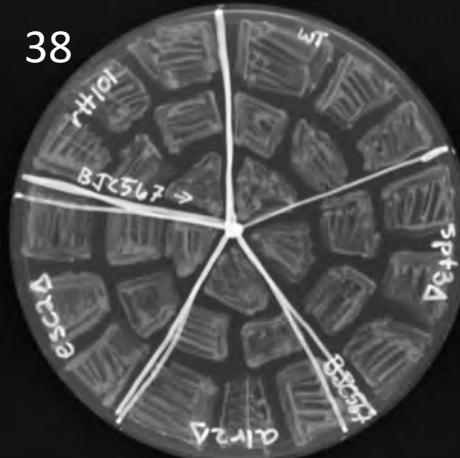

39

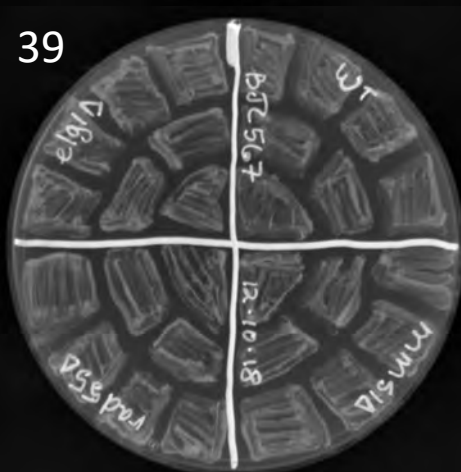

40

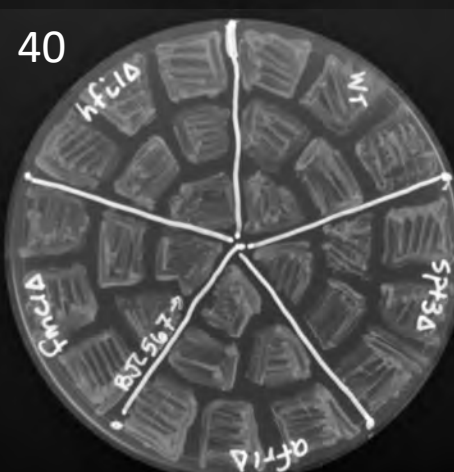

41

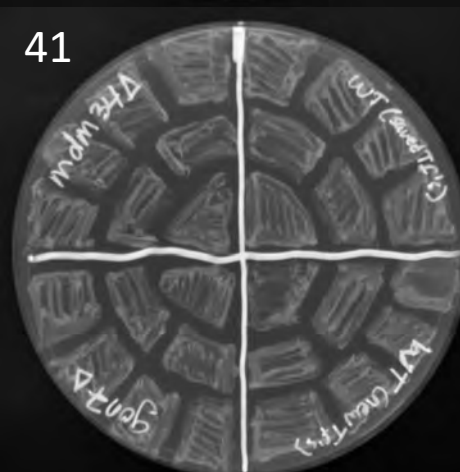

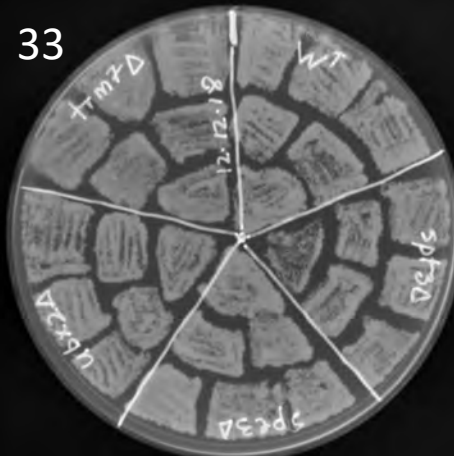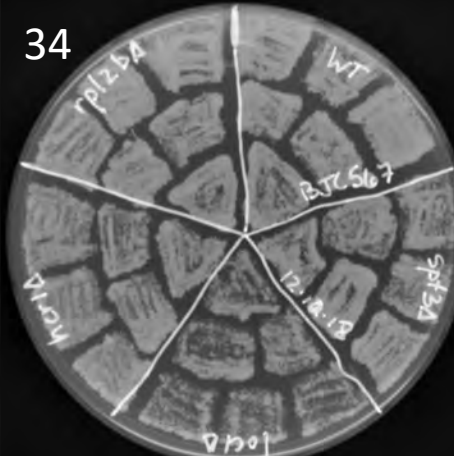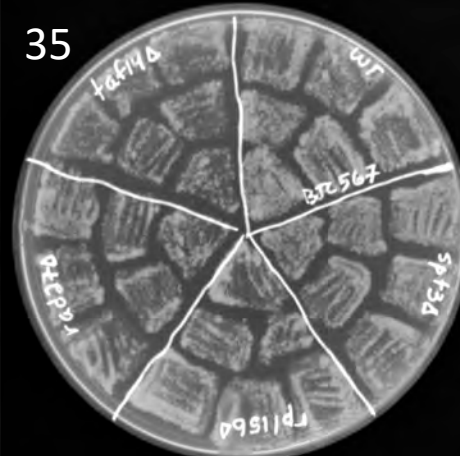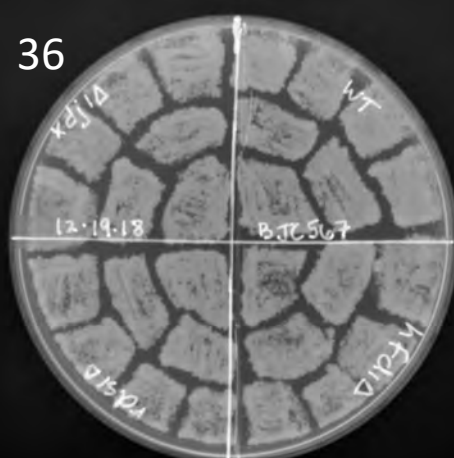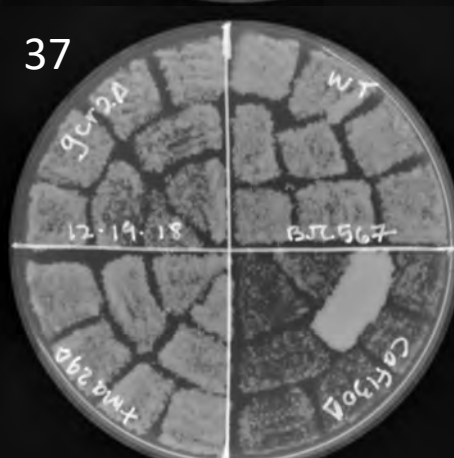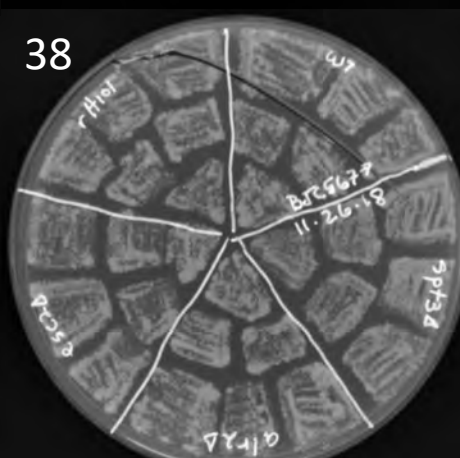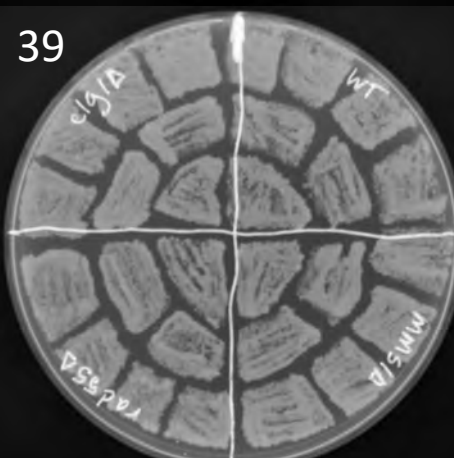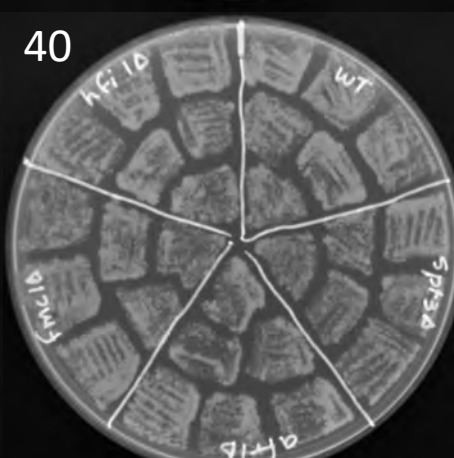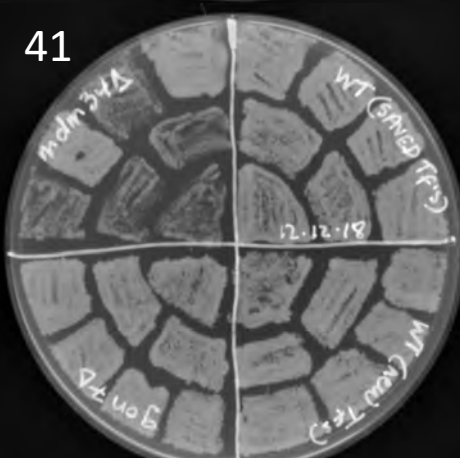

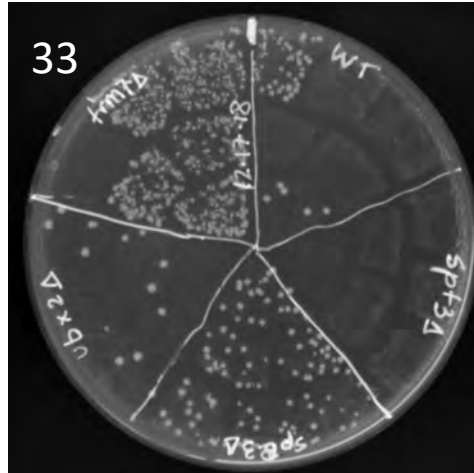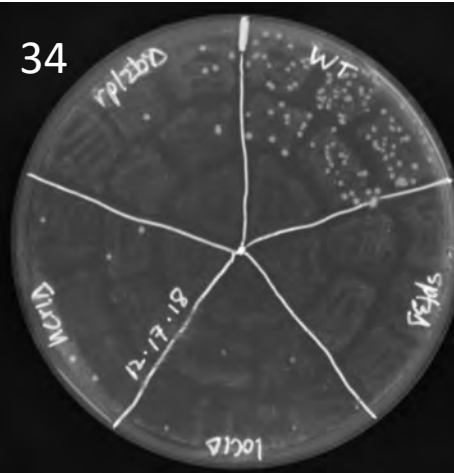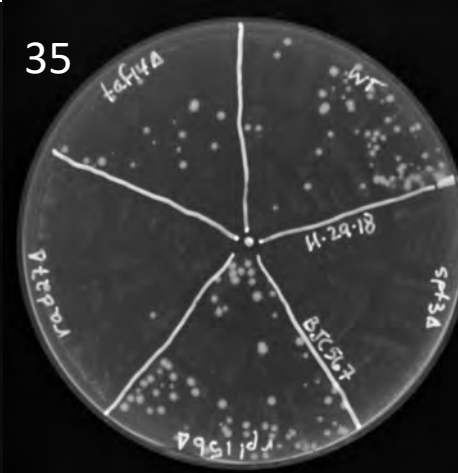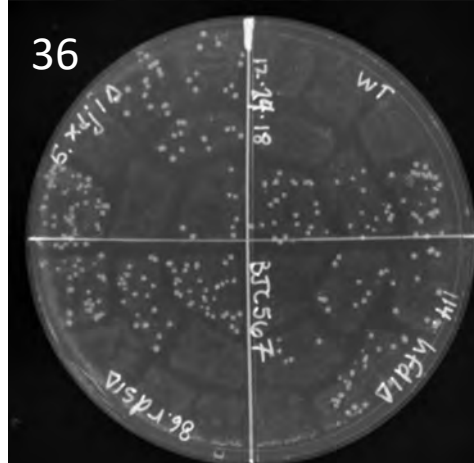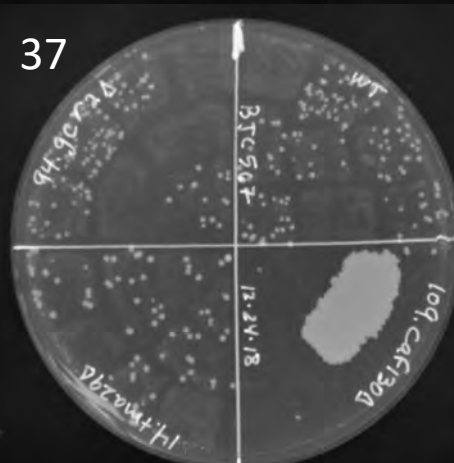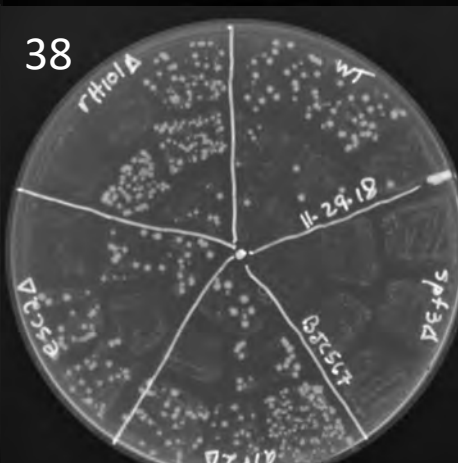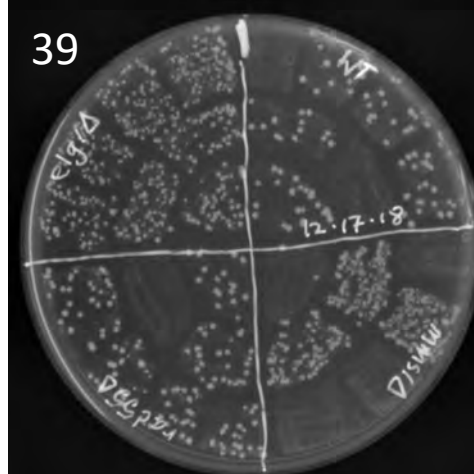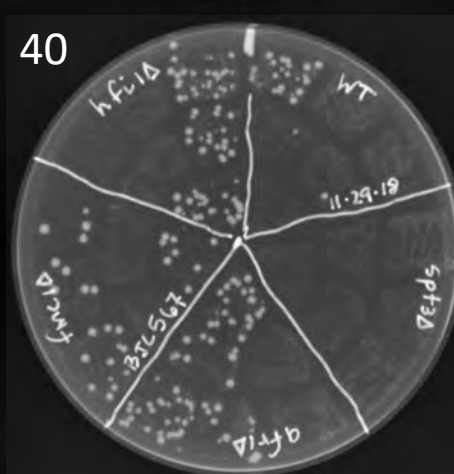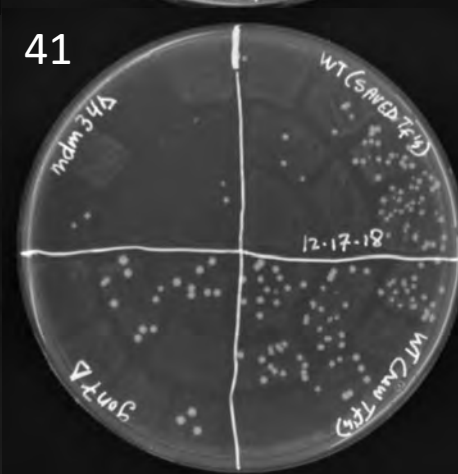

28

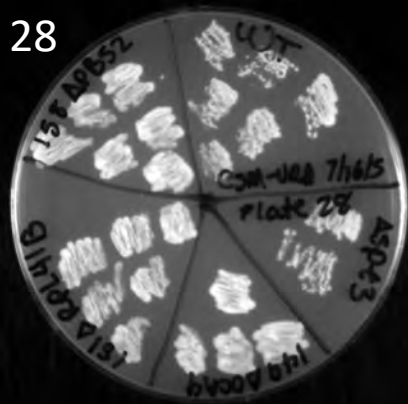

29

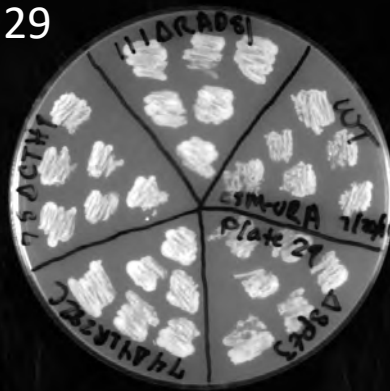

30

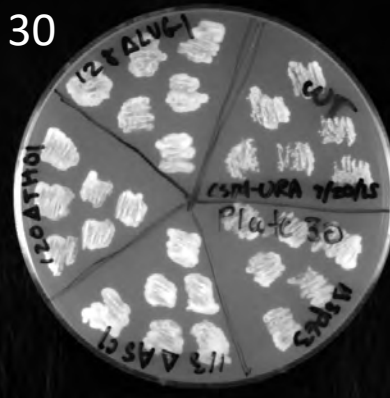

31

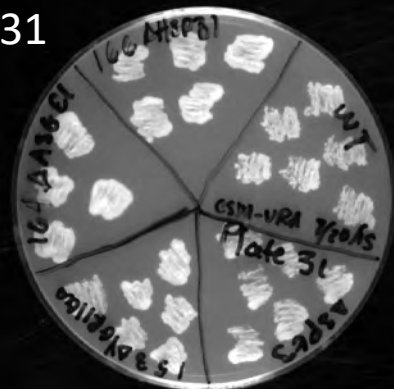

32

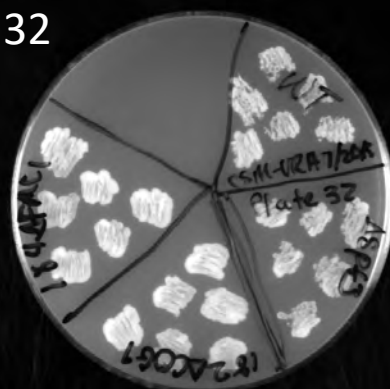

28

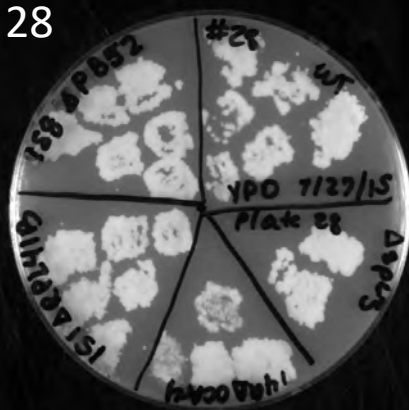

29

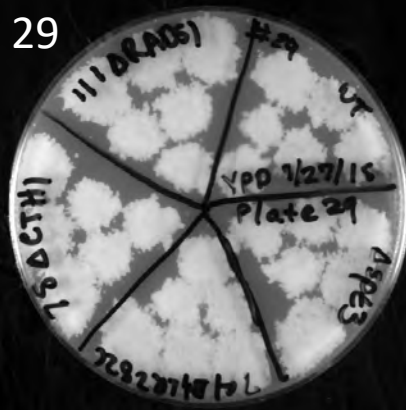

30

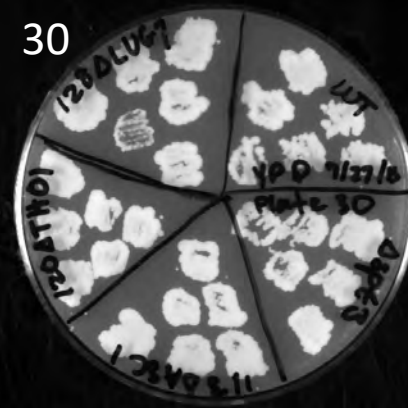

31

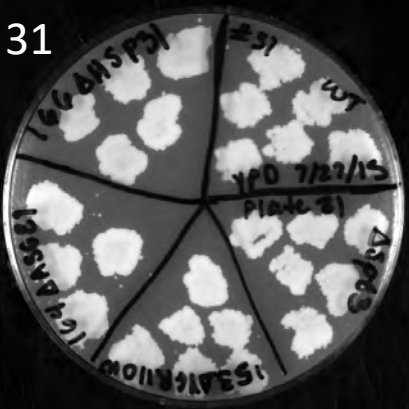

32

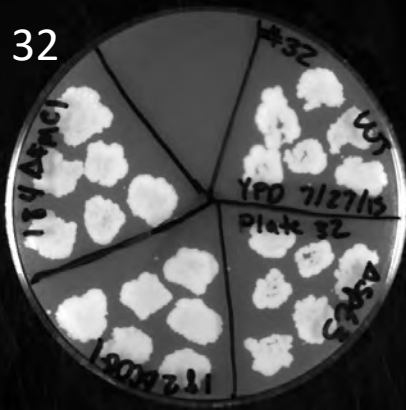

28

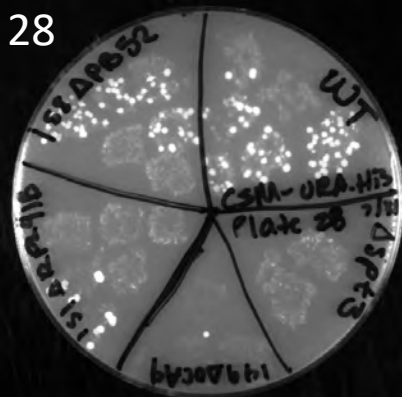

29

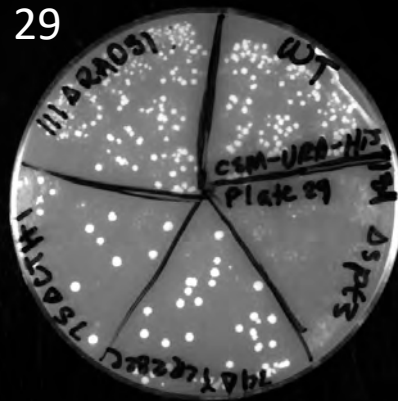

30

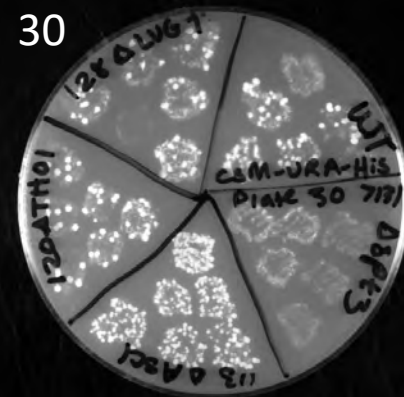

31

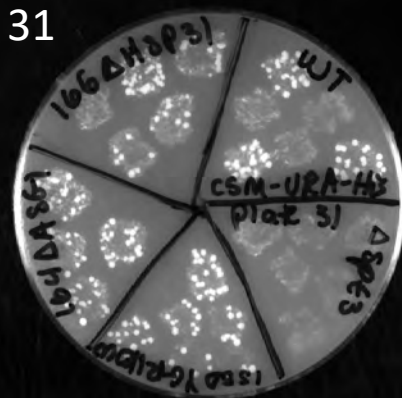

32

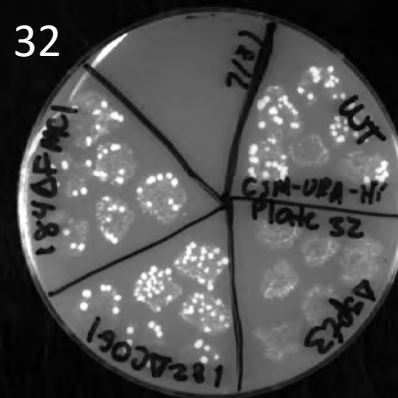

19

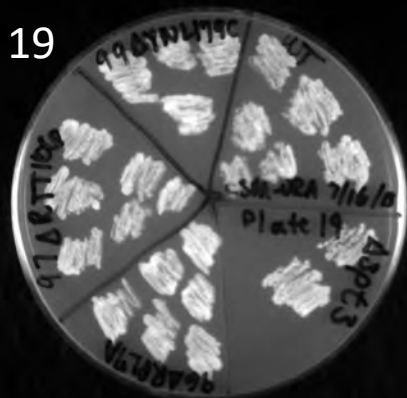

20

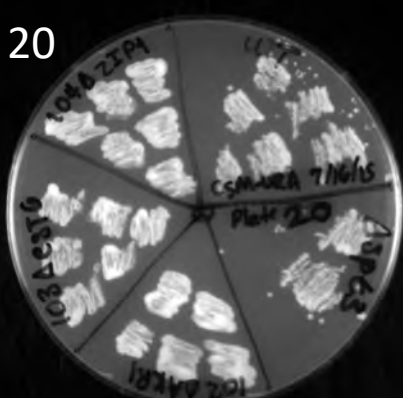

21

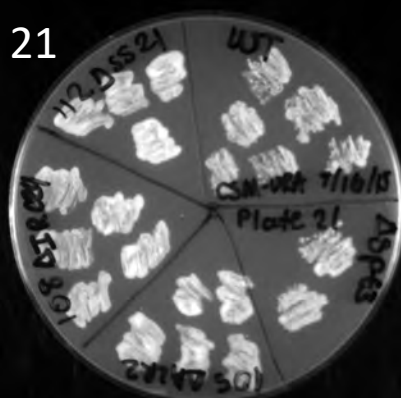

22

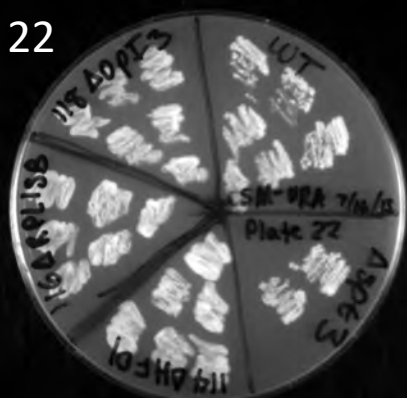

23

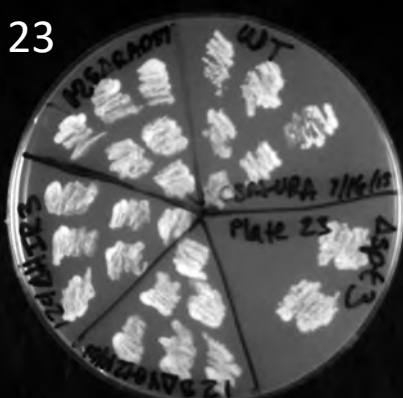

24

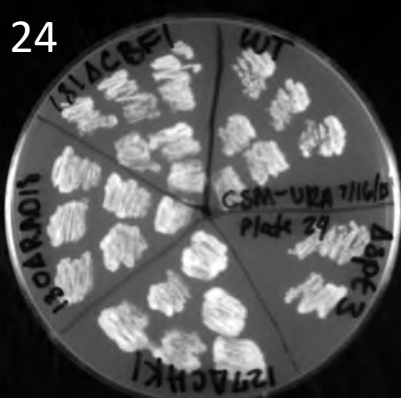

25

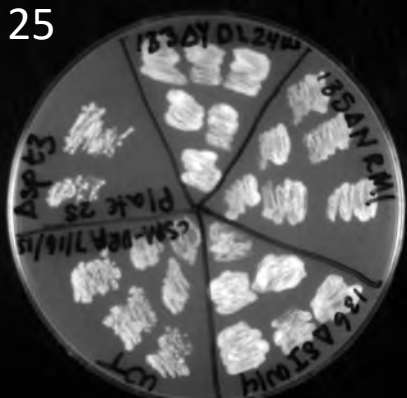

26

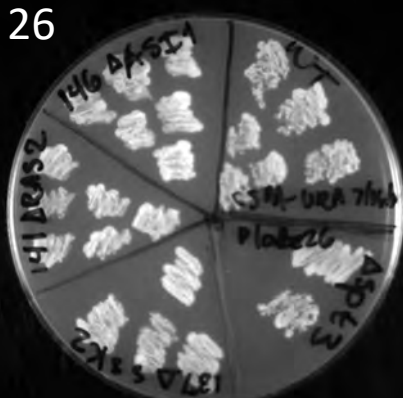

27

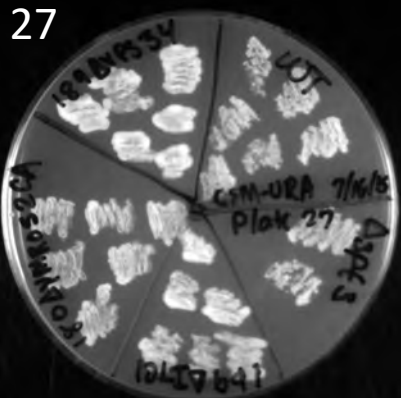

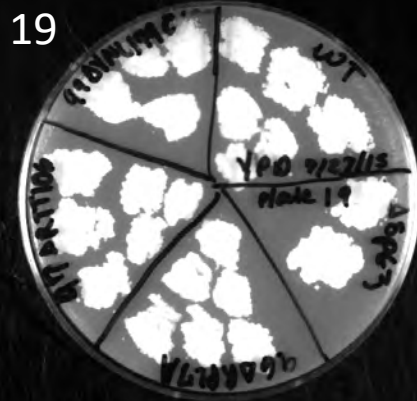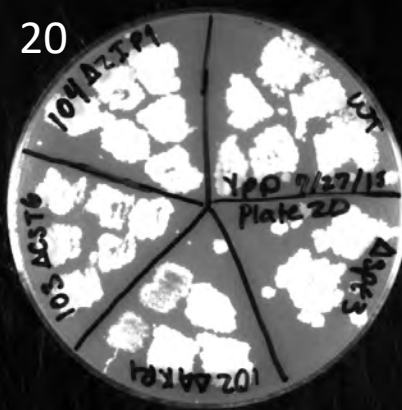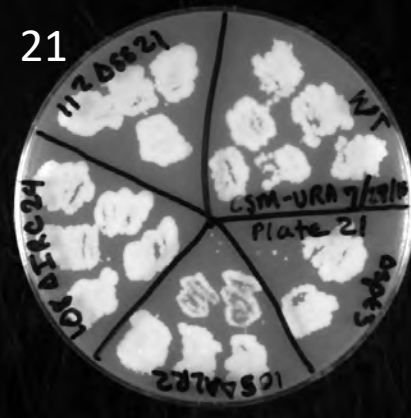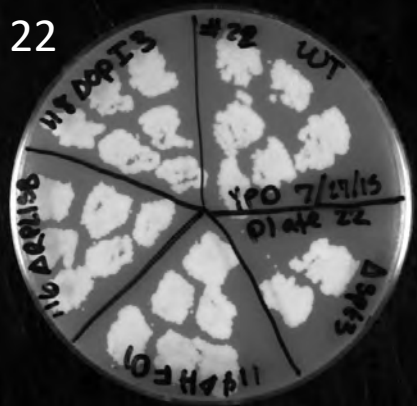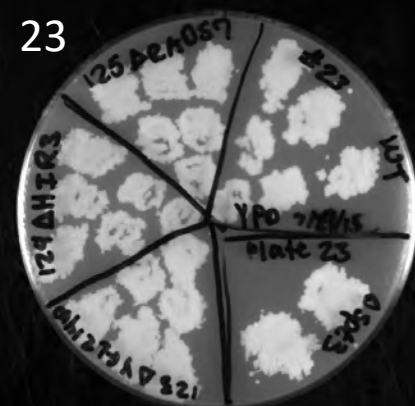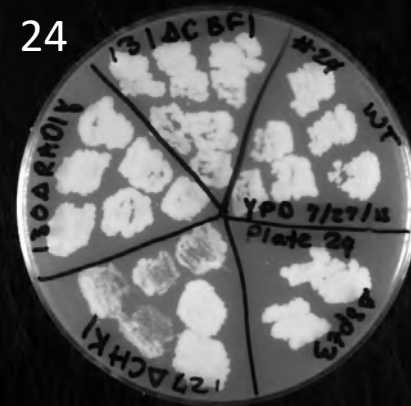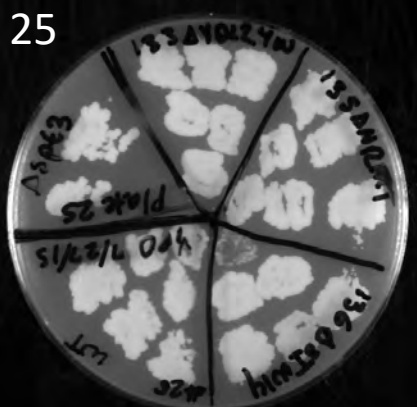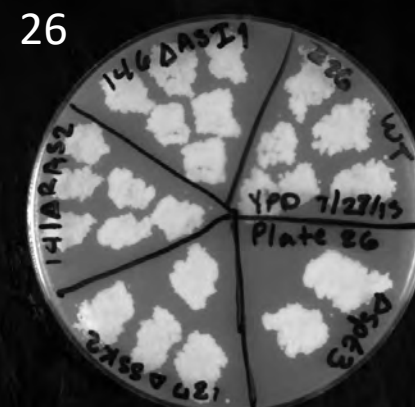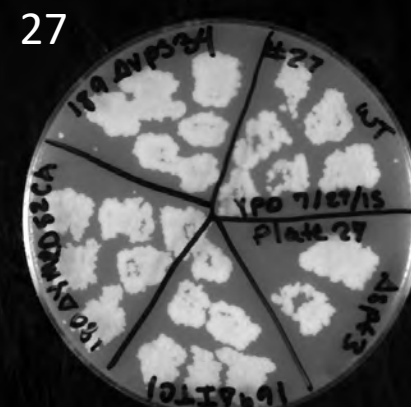

19

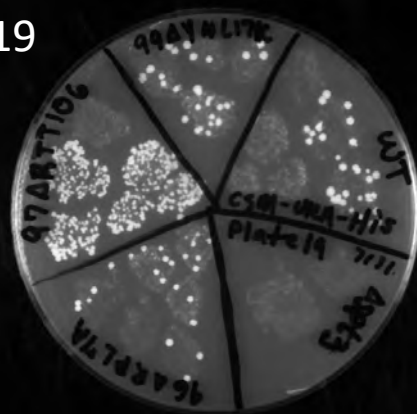

20

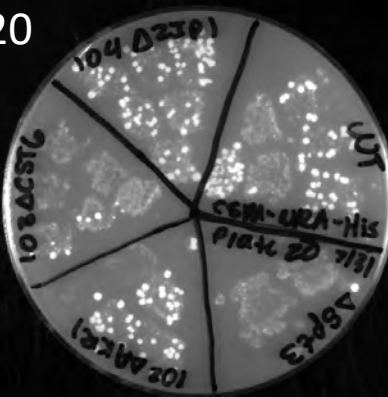

21

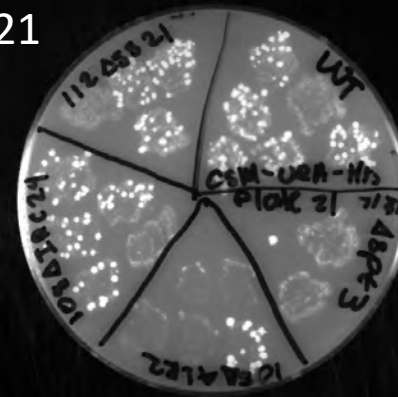

22

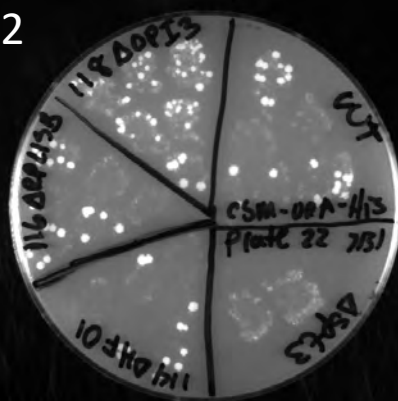

23

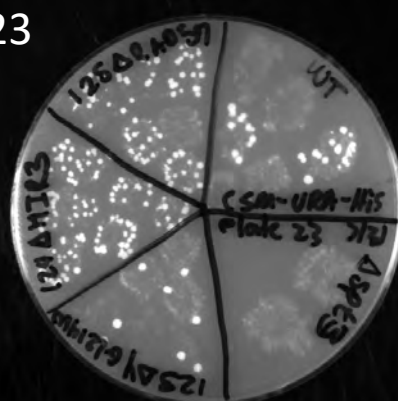

24

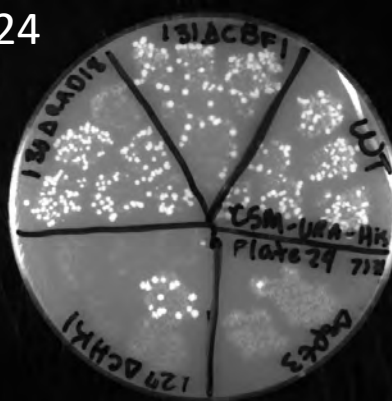

25

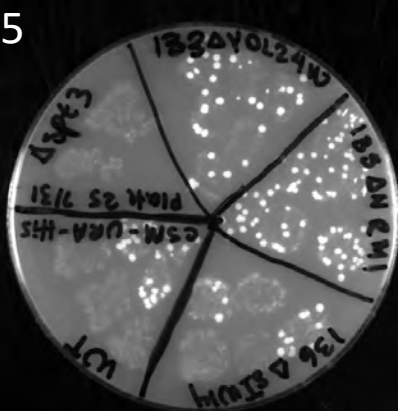

26

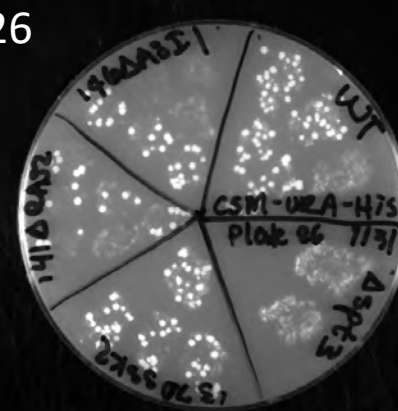

27

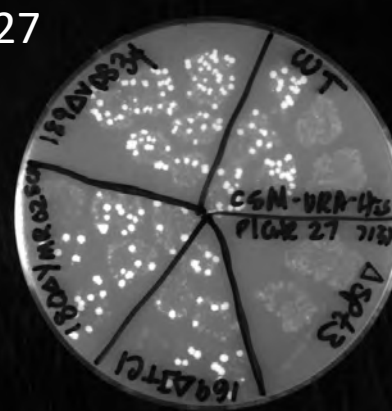

10

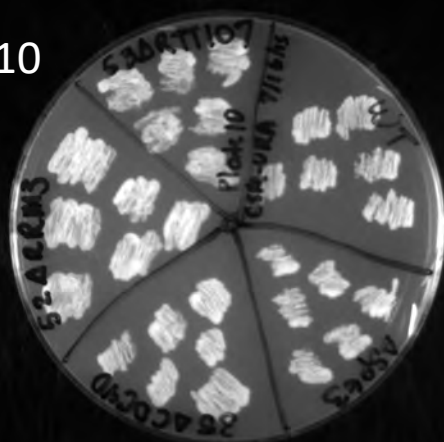

11

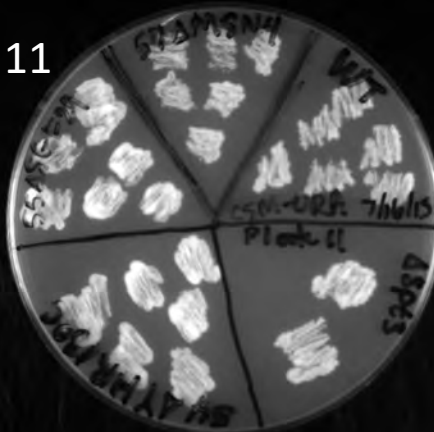

12

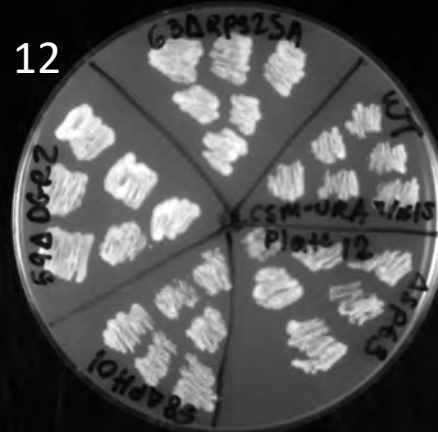

13

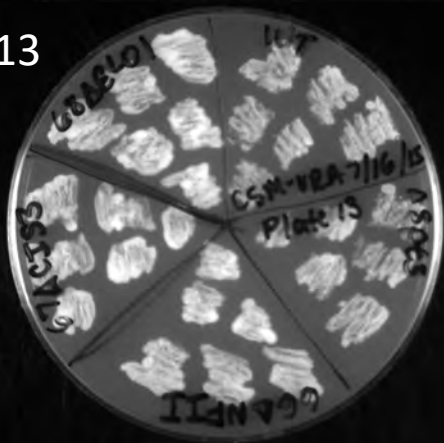

14

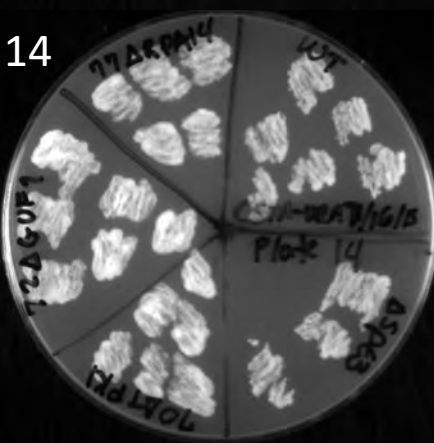

15

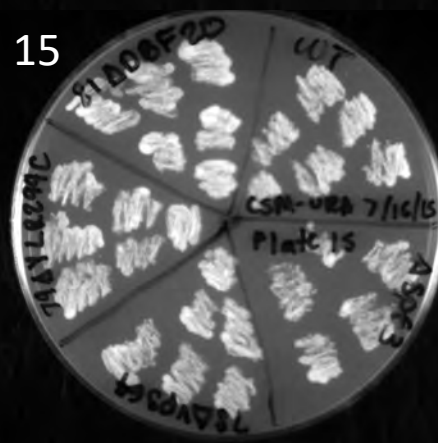

16

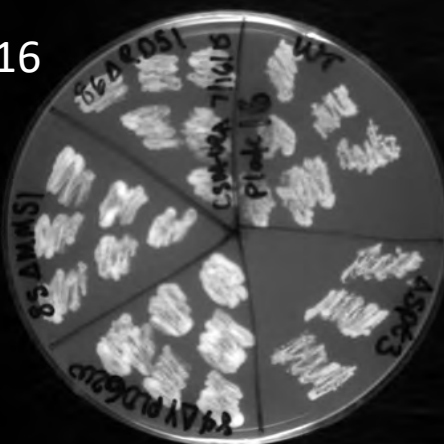

17

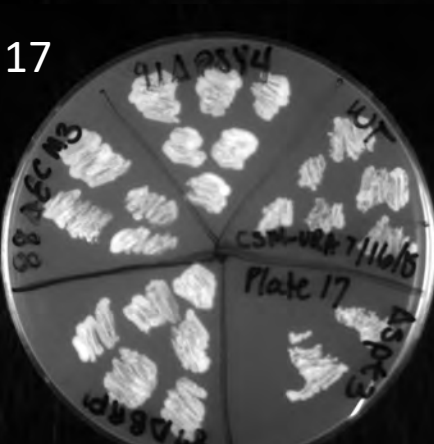

18

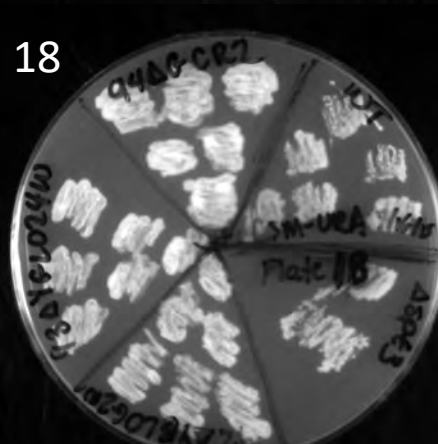

10

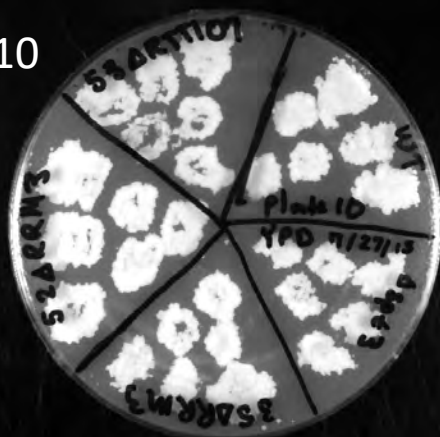

11

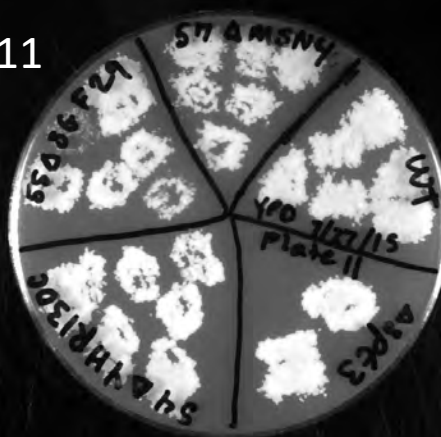

12

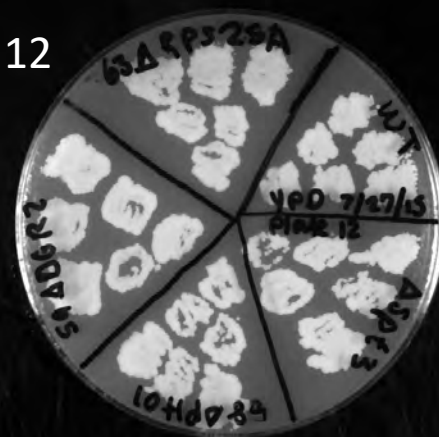

13

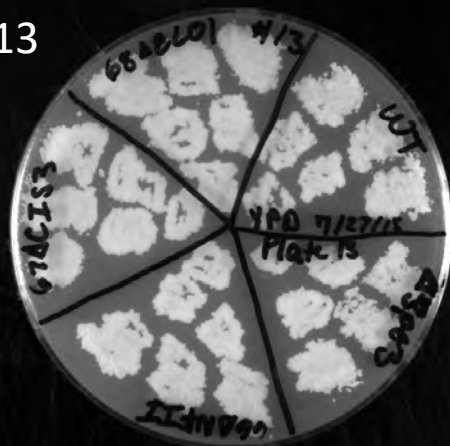

14

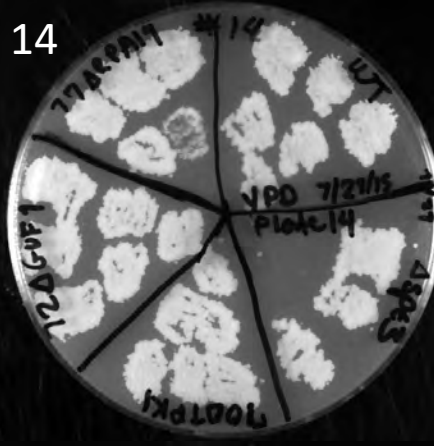

15

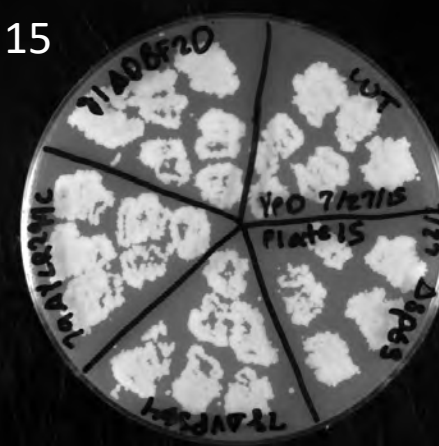

16

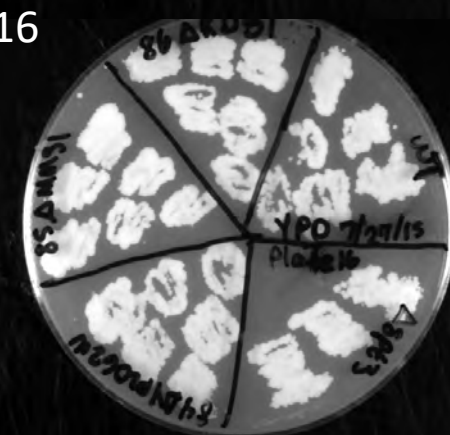

17

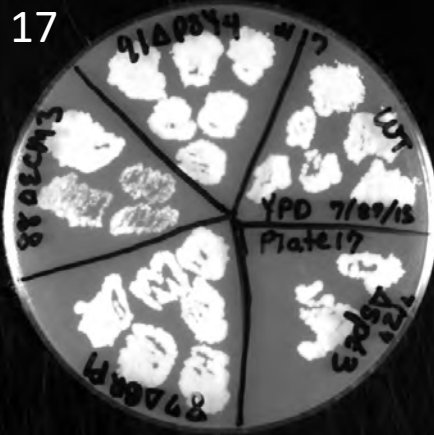

18

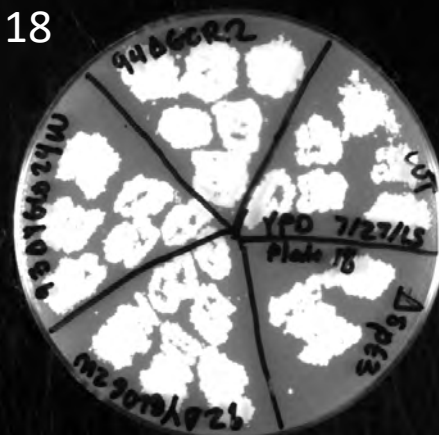

10

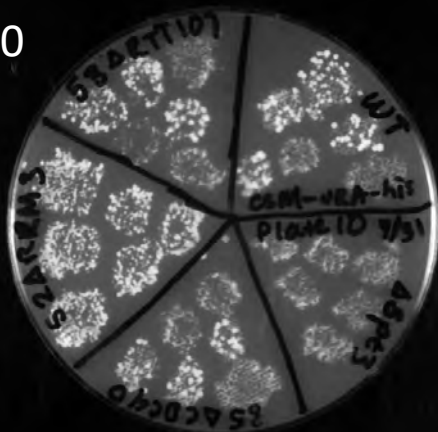

11

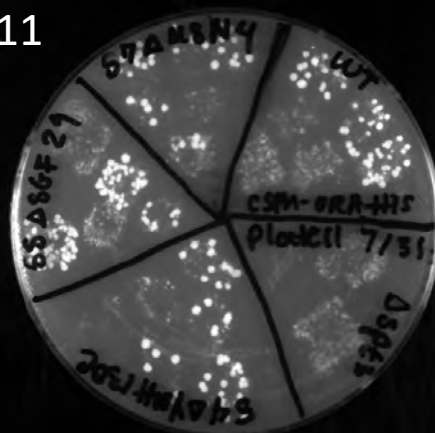

12

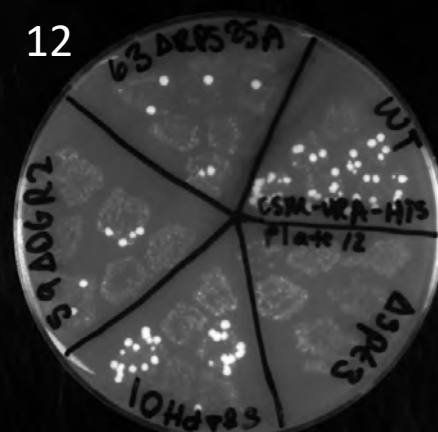

13

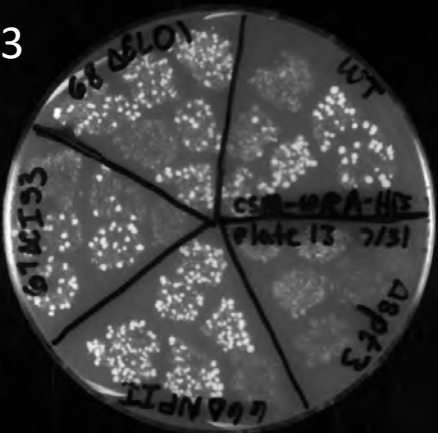

14

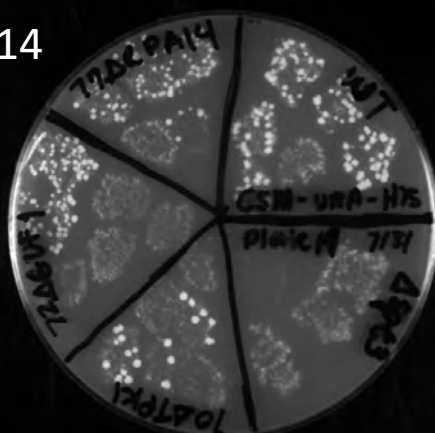

15

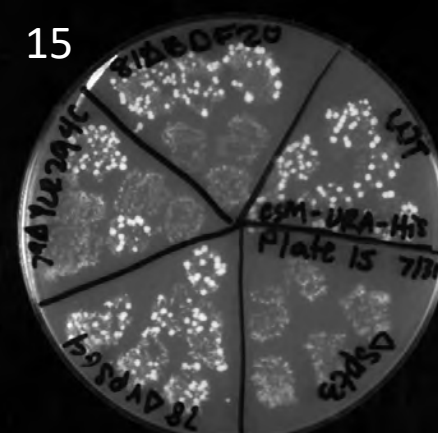

16

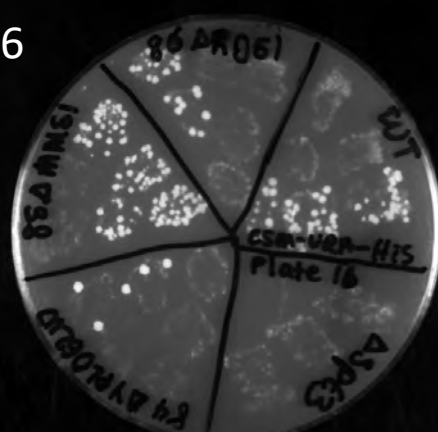

17

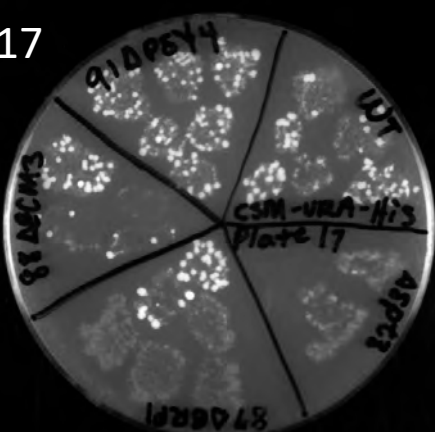

18

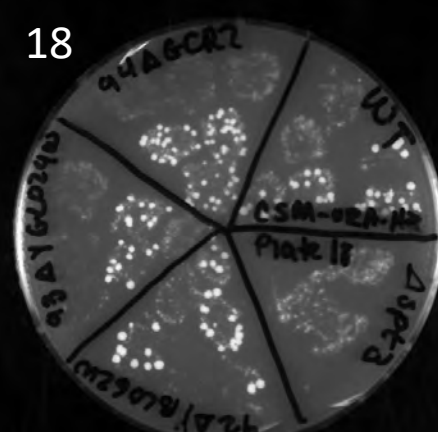

Supplement: Supplementary file 1 [file DataSheet2.PDF]
