# Supplementary material for: Reliance of Host-Encoded Regulators of Retromobility on Ty1 Promoter Activity or Architecture
Source: Front Mol Biosci. 2022 Jul 1;9:896215. doi: 10.3389/fmolb.2022.896215 (PMC9283973; doi:10.3389/fmolb.2022.896215)
Supplement: Supplementary file 4 [file Presentation1.PPTX]

## Slide 1
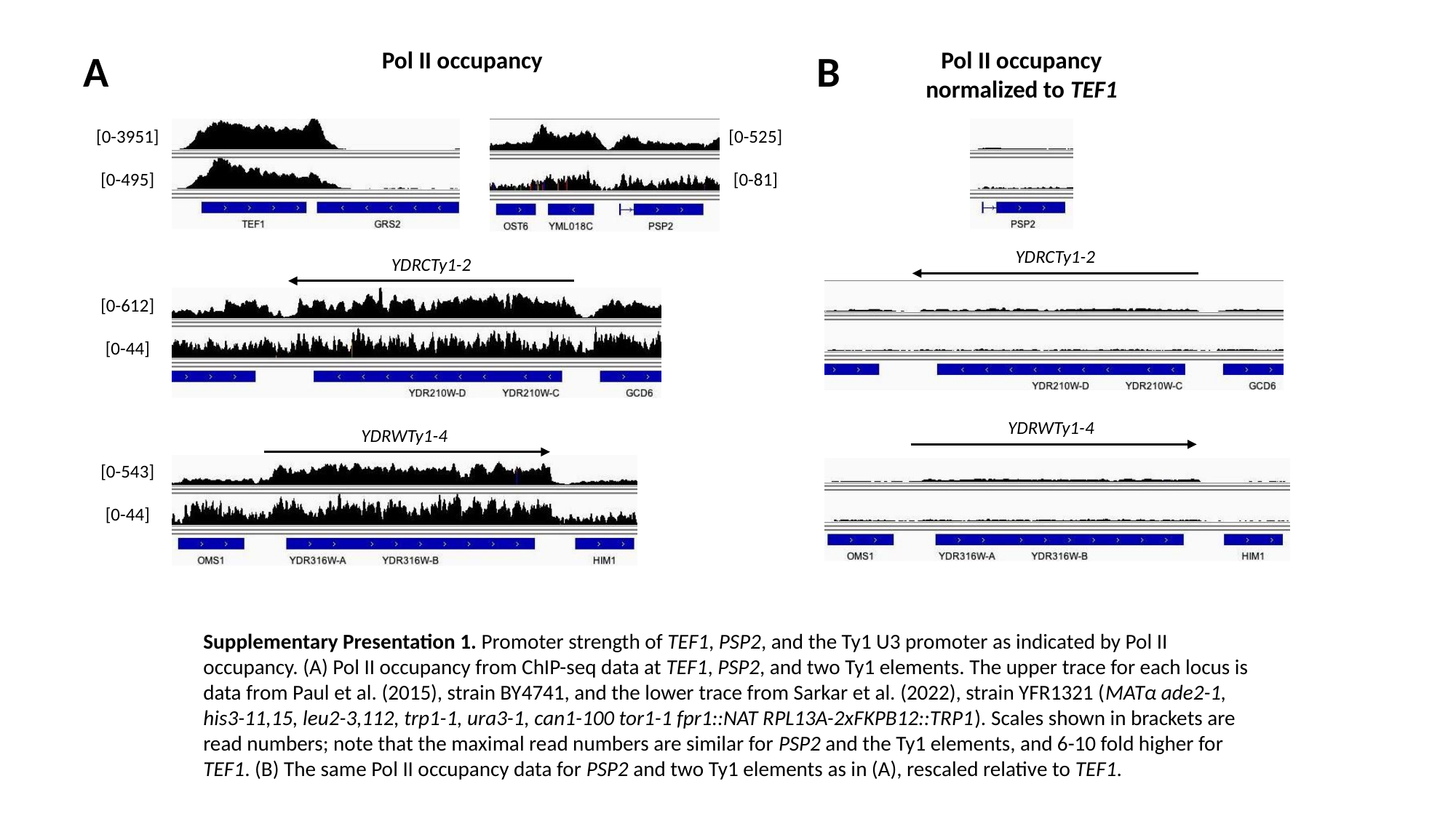

A
Pol II occupancy
B
Pol II occupancy normalized to TEF1
[0-3951]
[0-525]
[0-495]
[0-81]
YDRCTy1-2
YDRCTy1-2
[0-612]
[0-44]
YDRWTy1-4
YDRWTy1-4
[0-543]
[0-44]
Supplementary Presentation 1. Promoter strength of TEF1, PSP2, and the Ty1 U3 promoter as indicated by Pol II occupancy. (A) Pol II occupancy from ChIP-seq data at TEF1, PSP2, and two Ty1 elements. The upper trace for each locus is data from Paul et al. (2015), strain BY4741, and the lower trace from Sarkar et al. (2022), strain YFR1321 (MATα ade2-1, his3-11,15, leu2-3,112, trp1-1, ura3-1, can1-100 tor1-1 fpr1::NAT RPL13A-2xFKPB12::TRP1). Scales shown in brackets are read numbers; note that the maximal read numbers are similar for PSP2 and the Ty1 elements, and 6-10 fold higher for TEF1. (B) The same Pol II occupancy data for PSP2 and two Ty1 elements as in (A), rescaled relative to TEF1.
