# Supplementary material for: Reliance of Host-Encoded Regulators of Retromobility on Ty1 Promoter Activity or Architecture
Source: Front Mol Biosci. 2022 Jul 1;9:896215. doi: 10.3389/fmolb.2022.896215 (PMC9283973; doi:10.3389/fmolb.2022.896215)
Supplement: Supplementary file 5 [file DataSheet3.PDF]

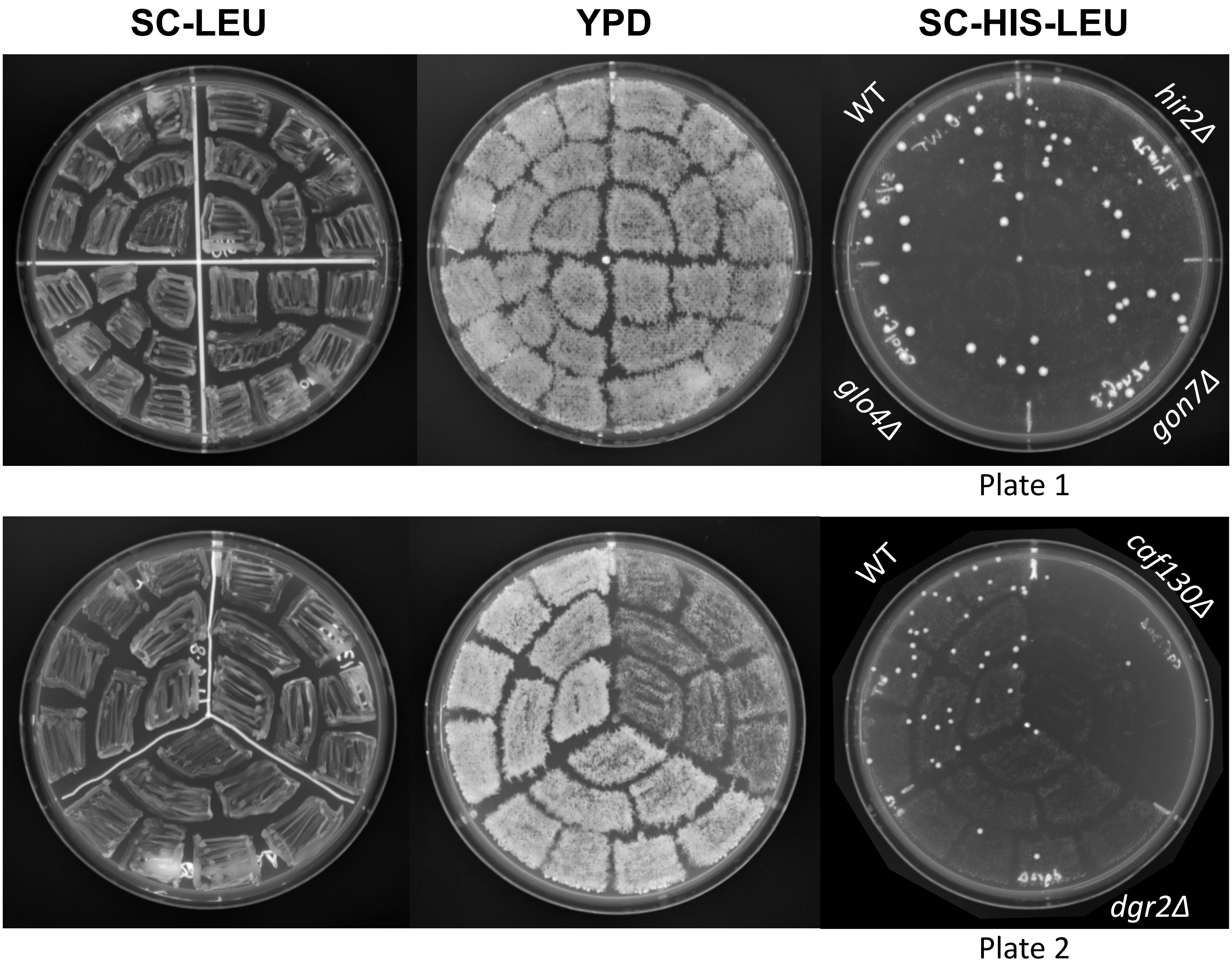
SC-LEU
YPD
SC-HIS-LEU
WT
hir2Δ
glo4Δ
gon7Δ
Plate 1
WT
caf130Δ
dgr2Δ
Plate 2

SC-HIS-LEU plates

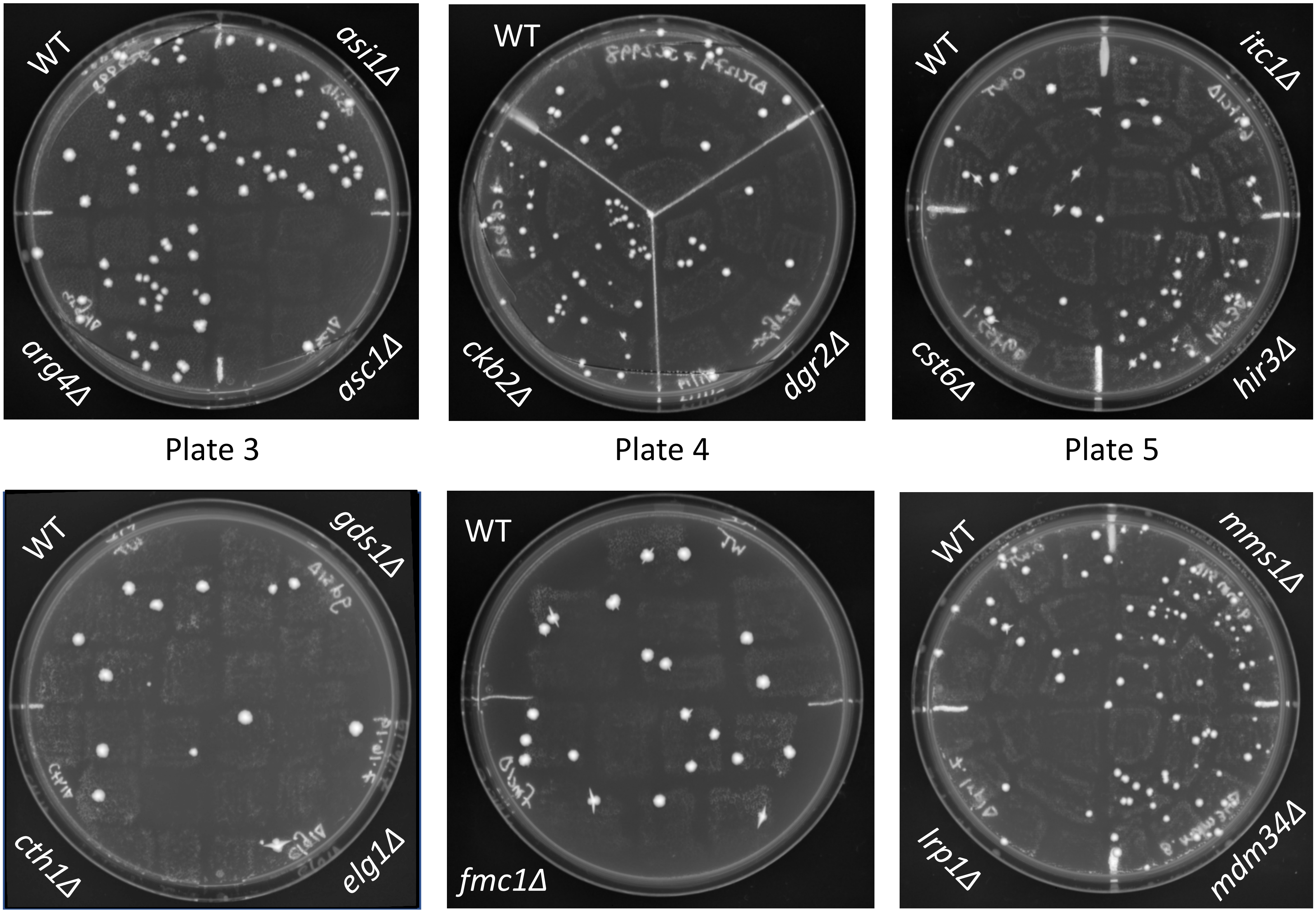

Plate 6 Plate 7 Plate 8

SC-HIS-LEU plates

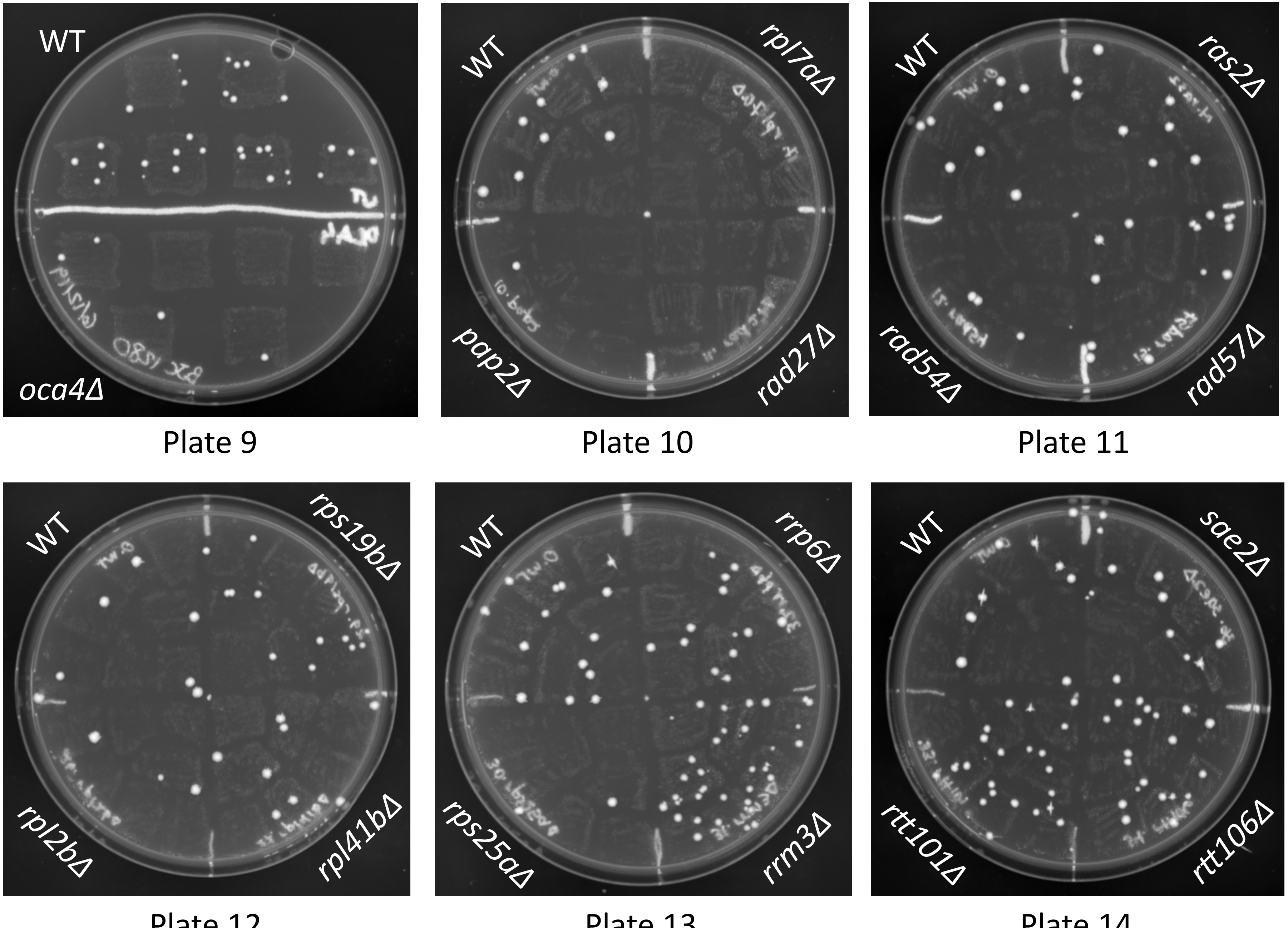

SC-HIS-LEU plates

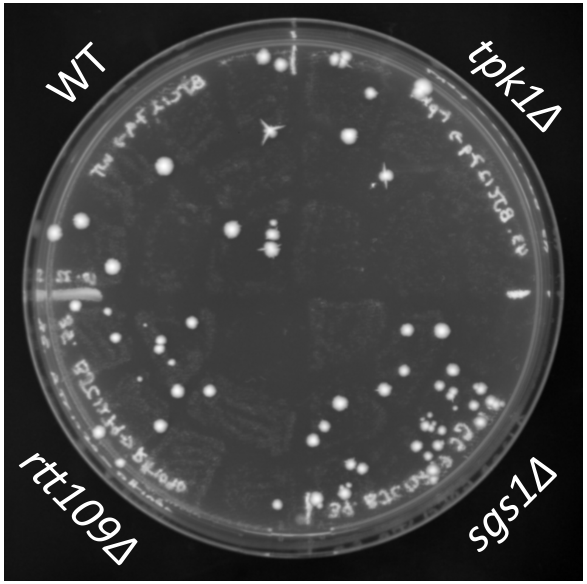

Plate 15

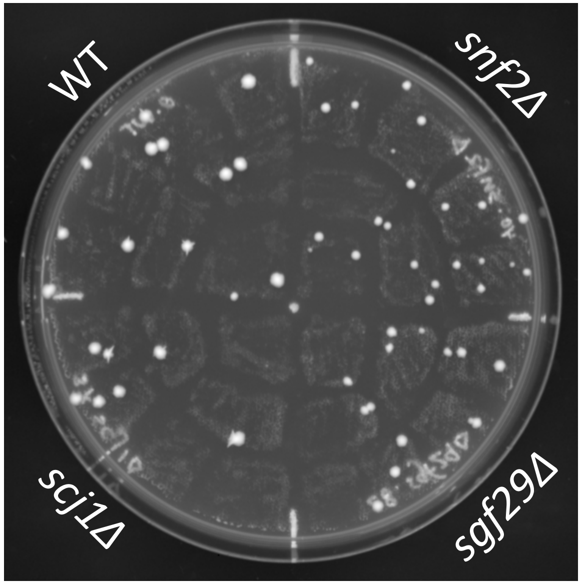

Plate 16

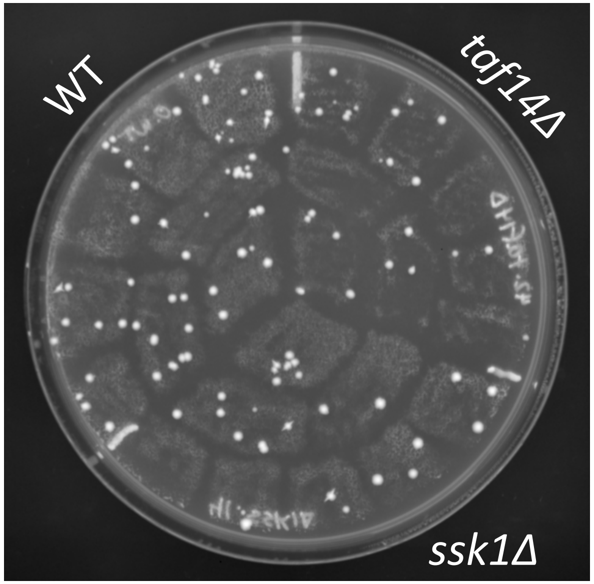

Plate 17

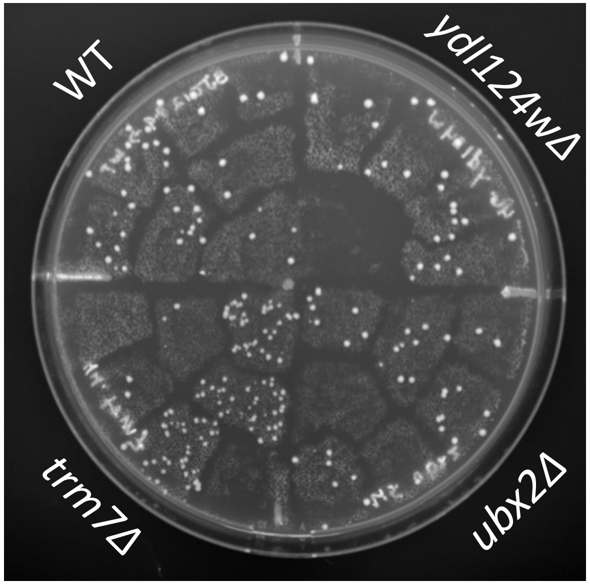

Plate 18

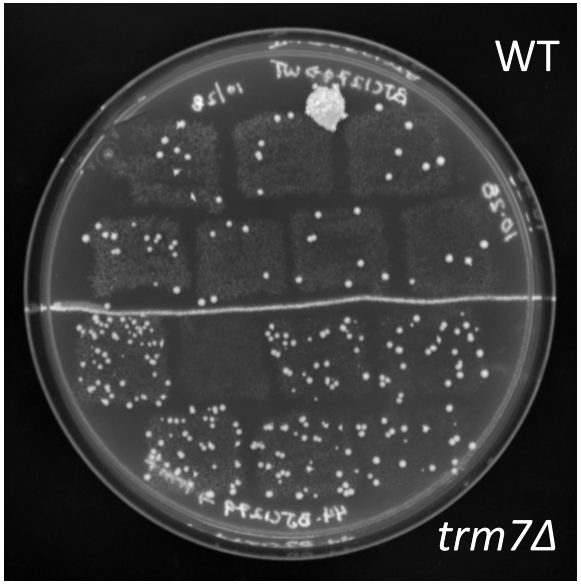

Plate 19

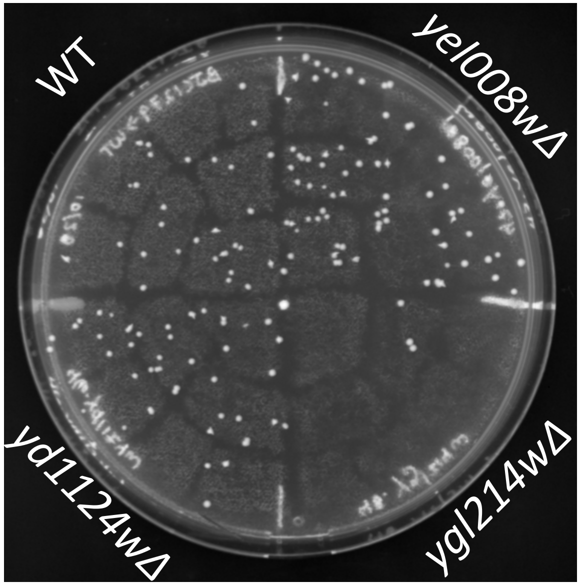

Plate 20

SC-HIS-LEU plates

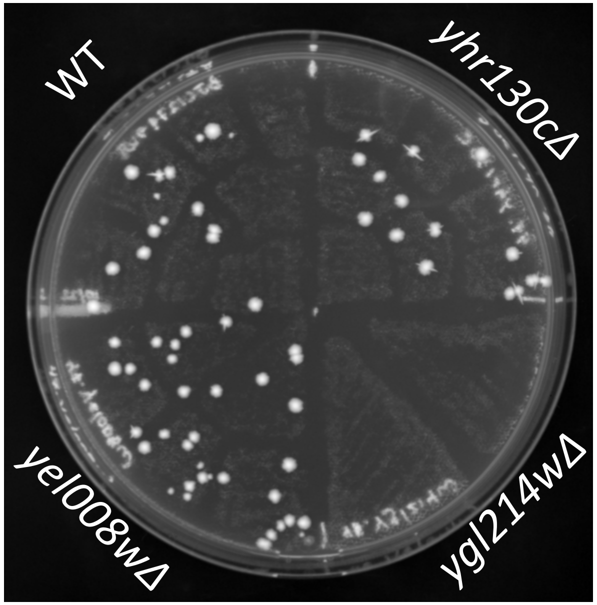

Plate 21

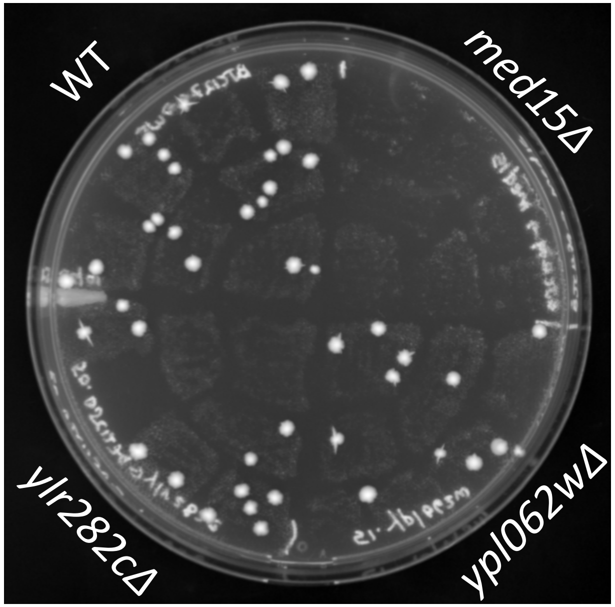

Plate 22
