# Supplementary material for: Reliance of Host-Encoded Regulators of Retromobility on Ty1 Promoter Activity or Architecture
Source: Front Mol Biosci. 2022 Jul 1;9:896215. doi: 10.3389/fmolb.2022.896215 (PMC9283973; doi:10.3389/fmolb.2022.896215)

Plate 0

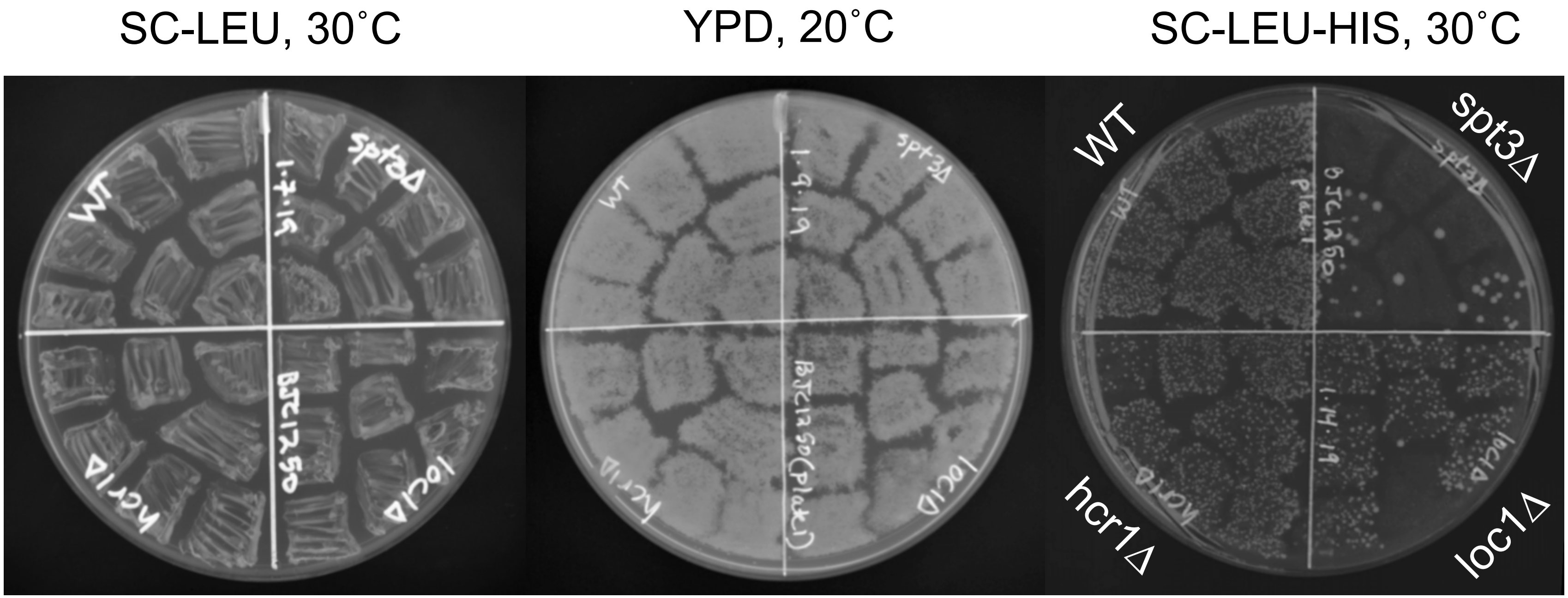

SC-LEU

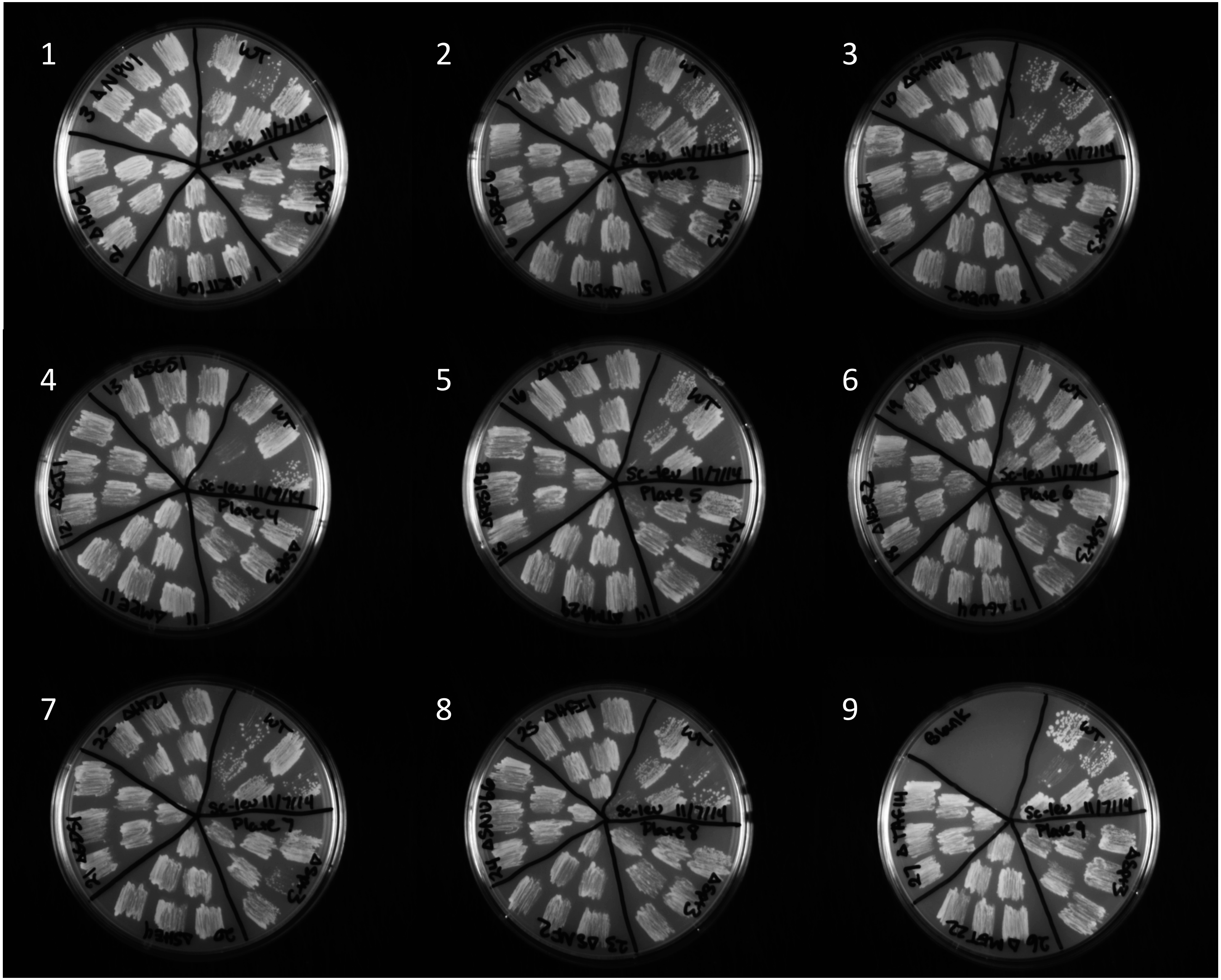

YPD

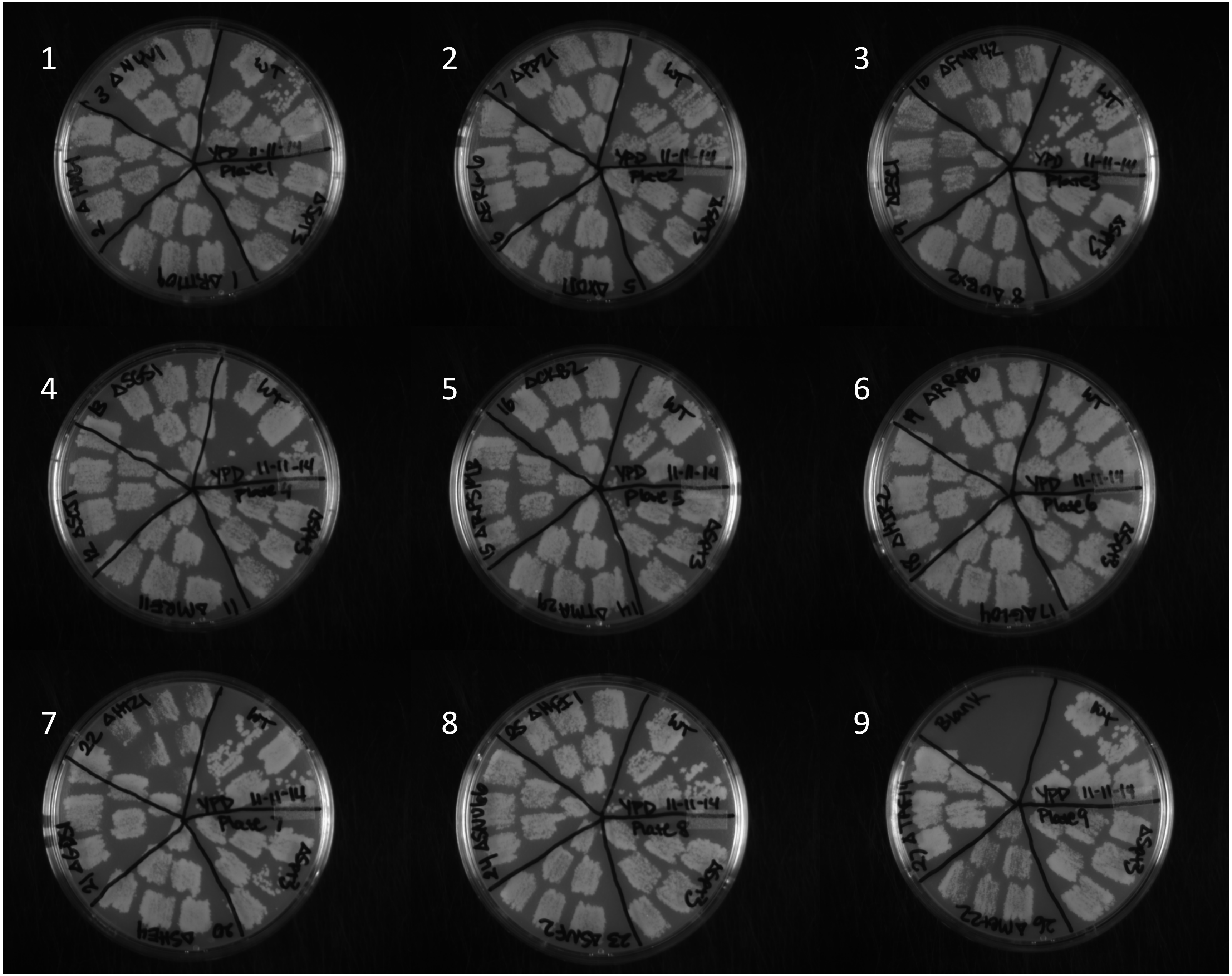

SC-HIS-LEU

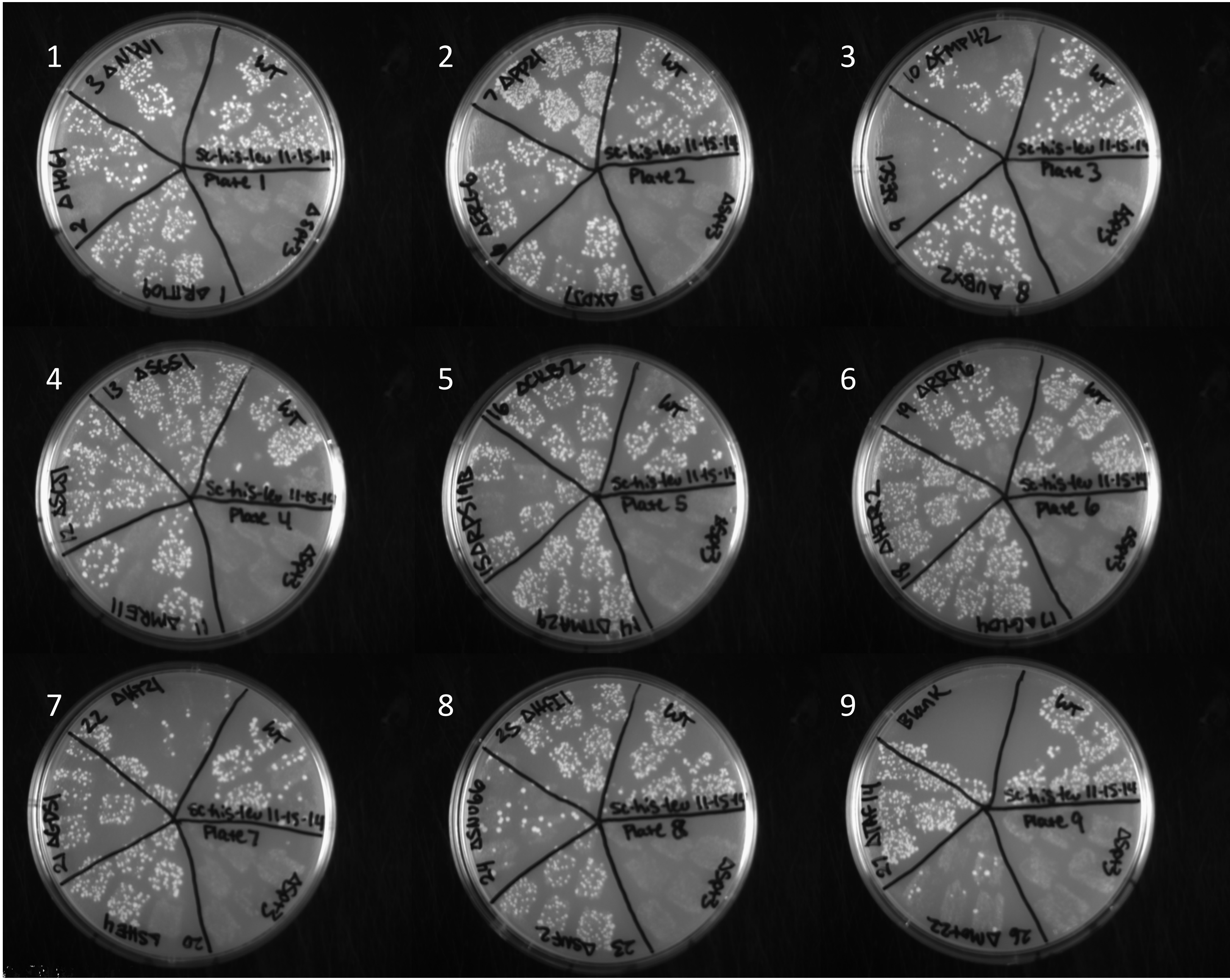

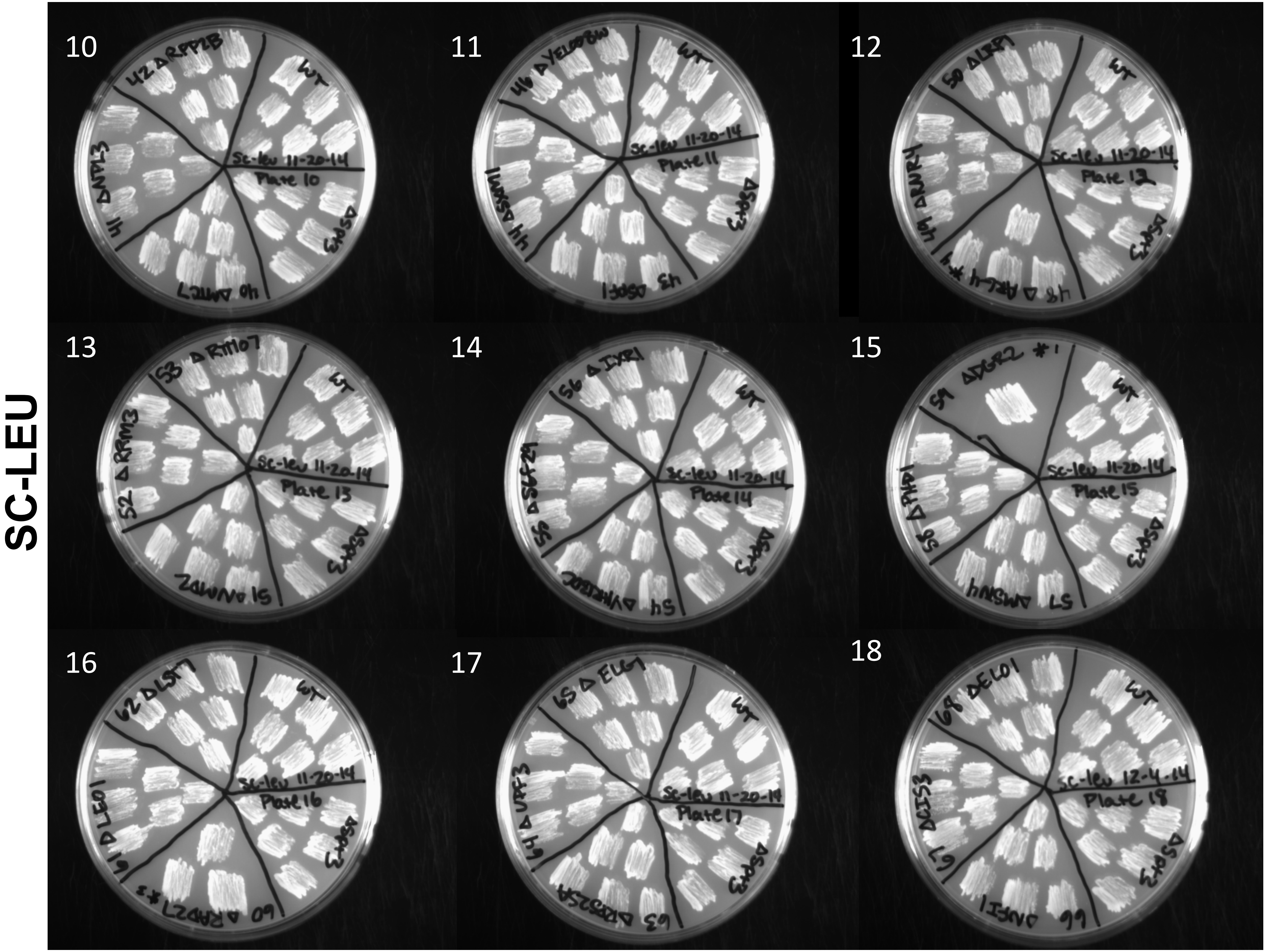
SC-LEU
10
11
12
13
14
15
16
17
18

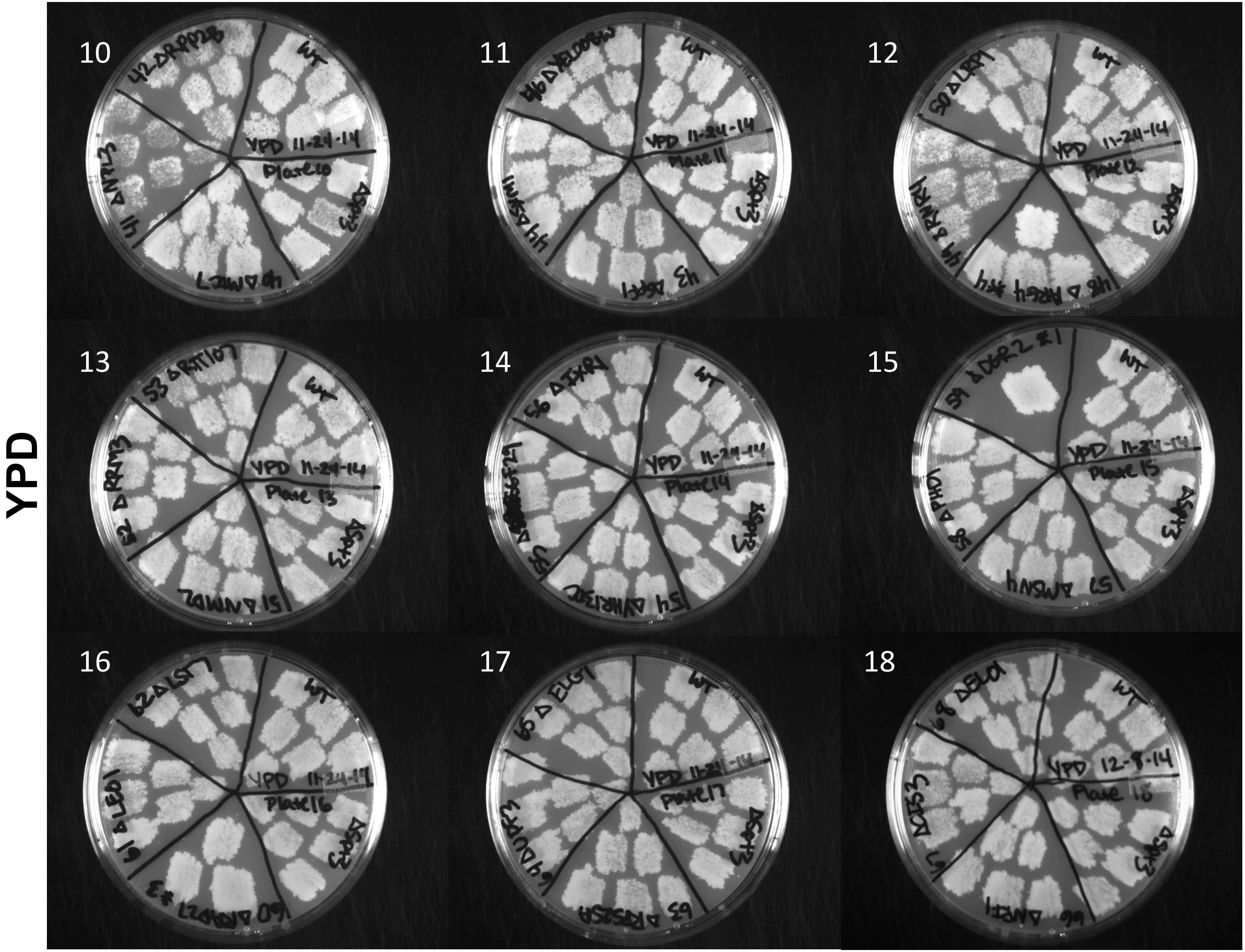
YPD
10
11
12
13
14
15
16
17
18

SC-HIS-LEU

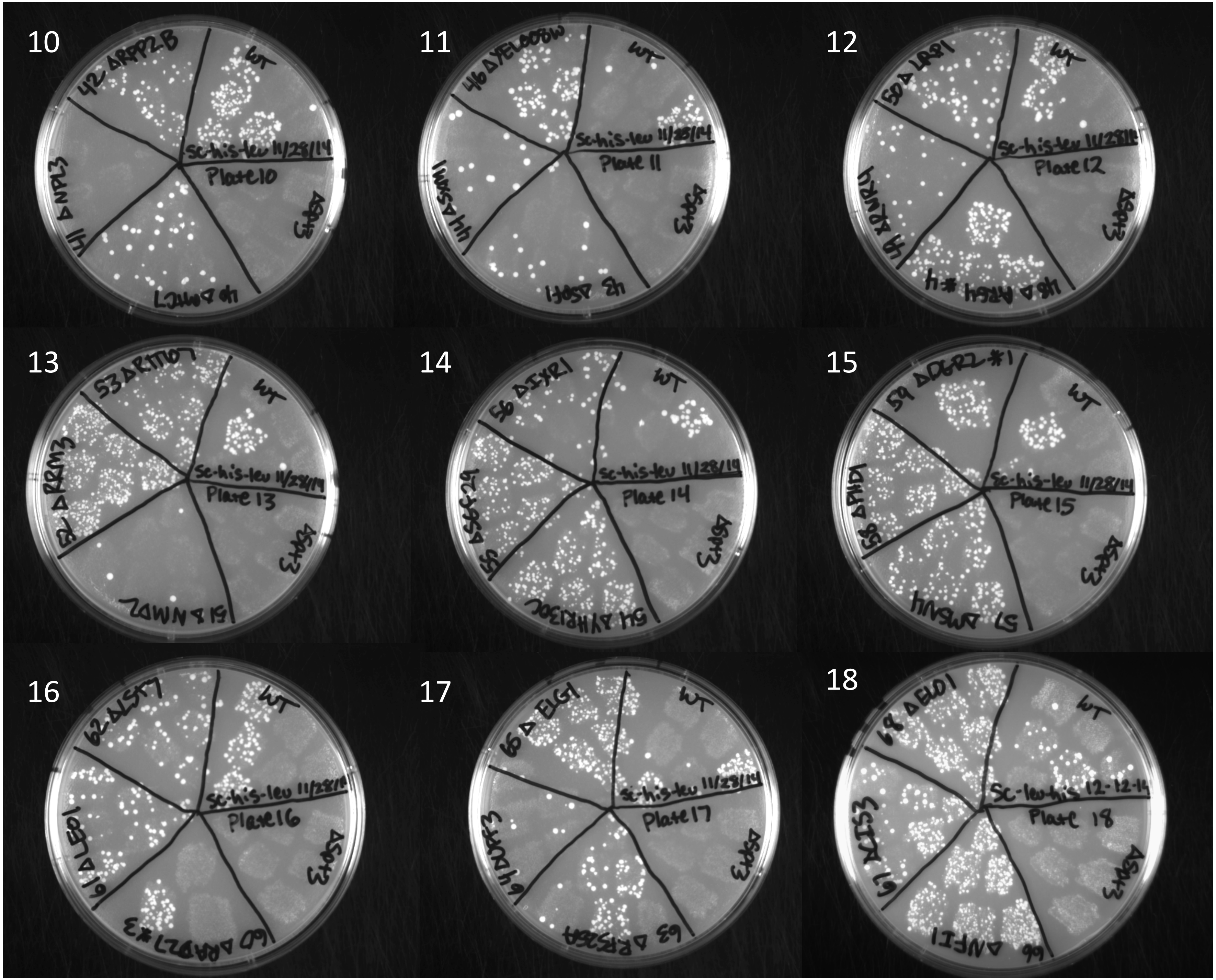

SC-LEU

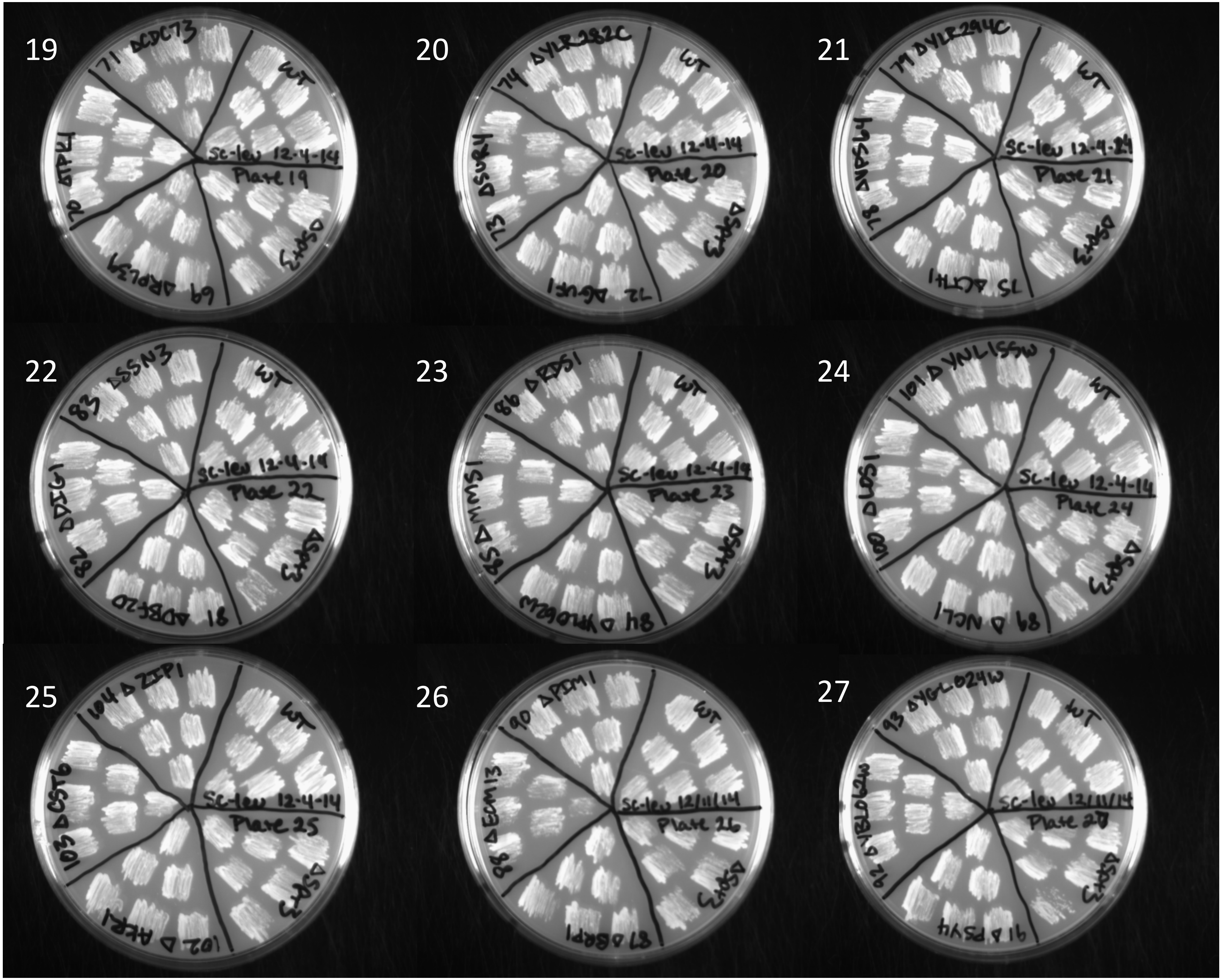

YPD

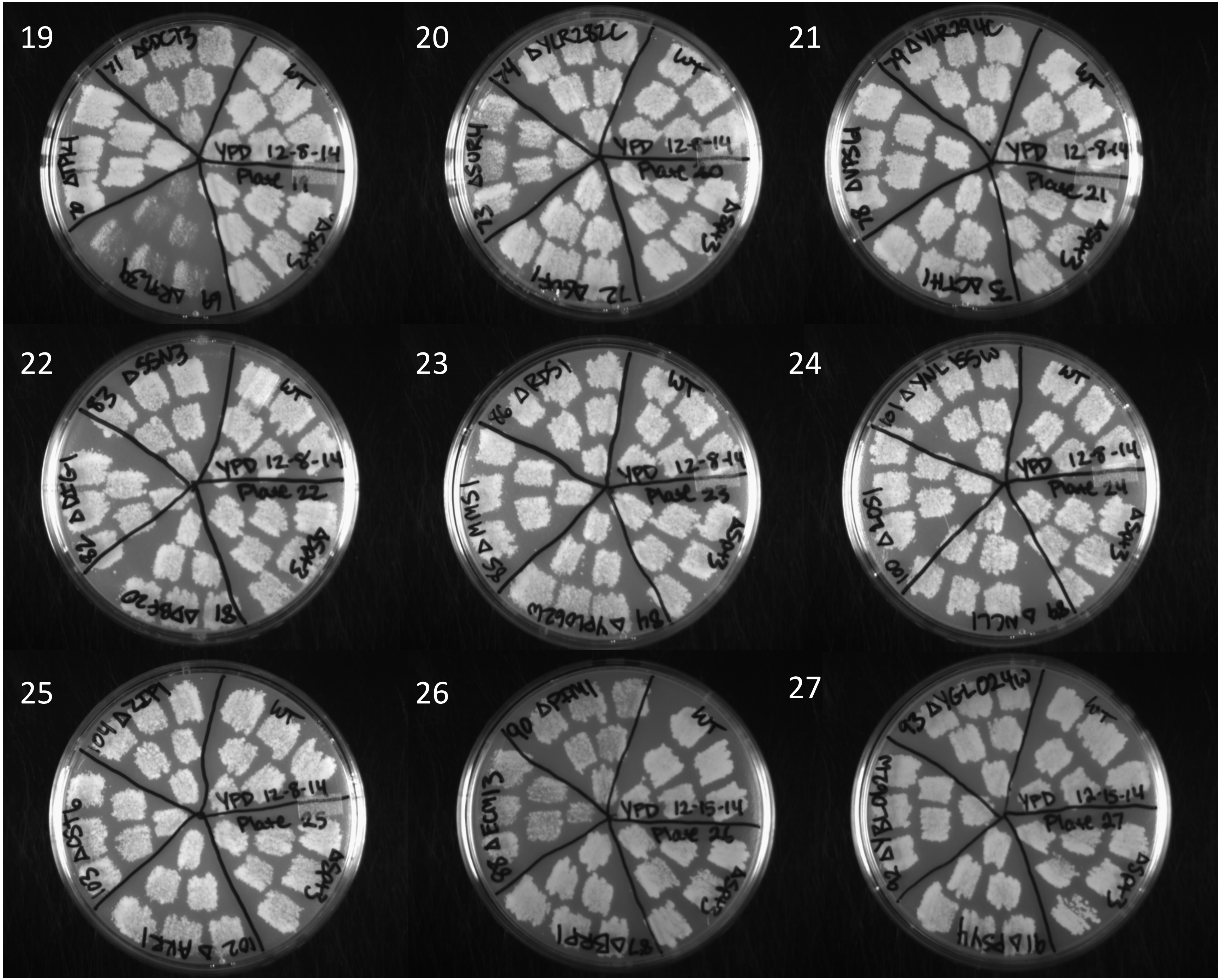

SC-HIS-LEU

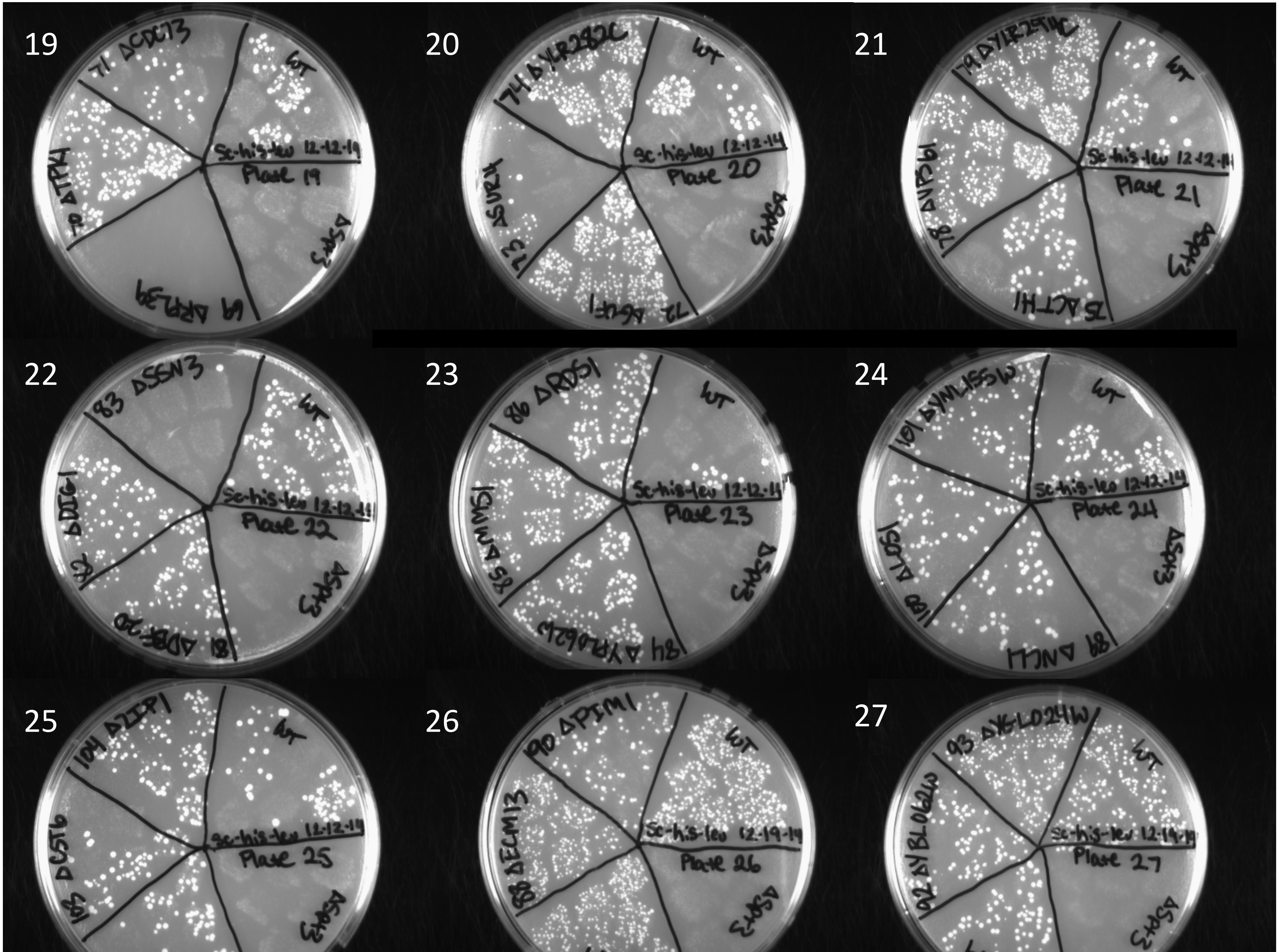

SC-LEU

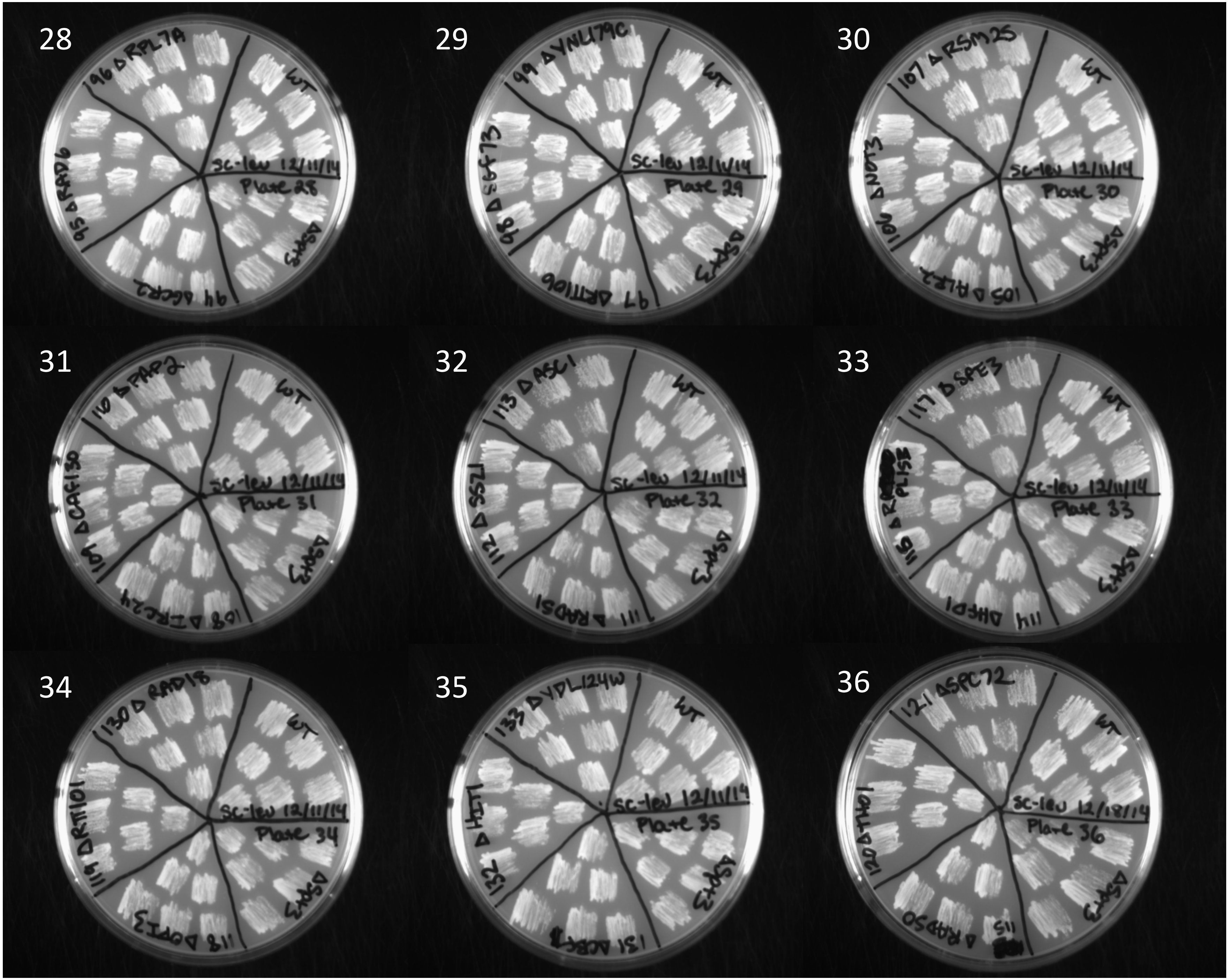

YPD

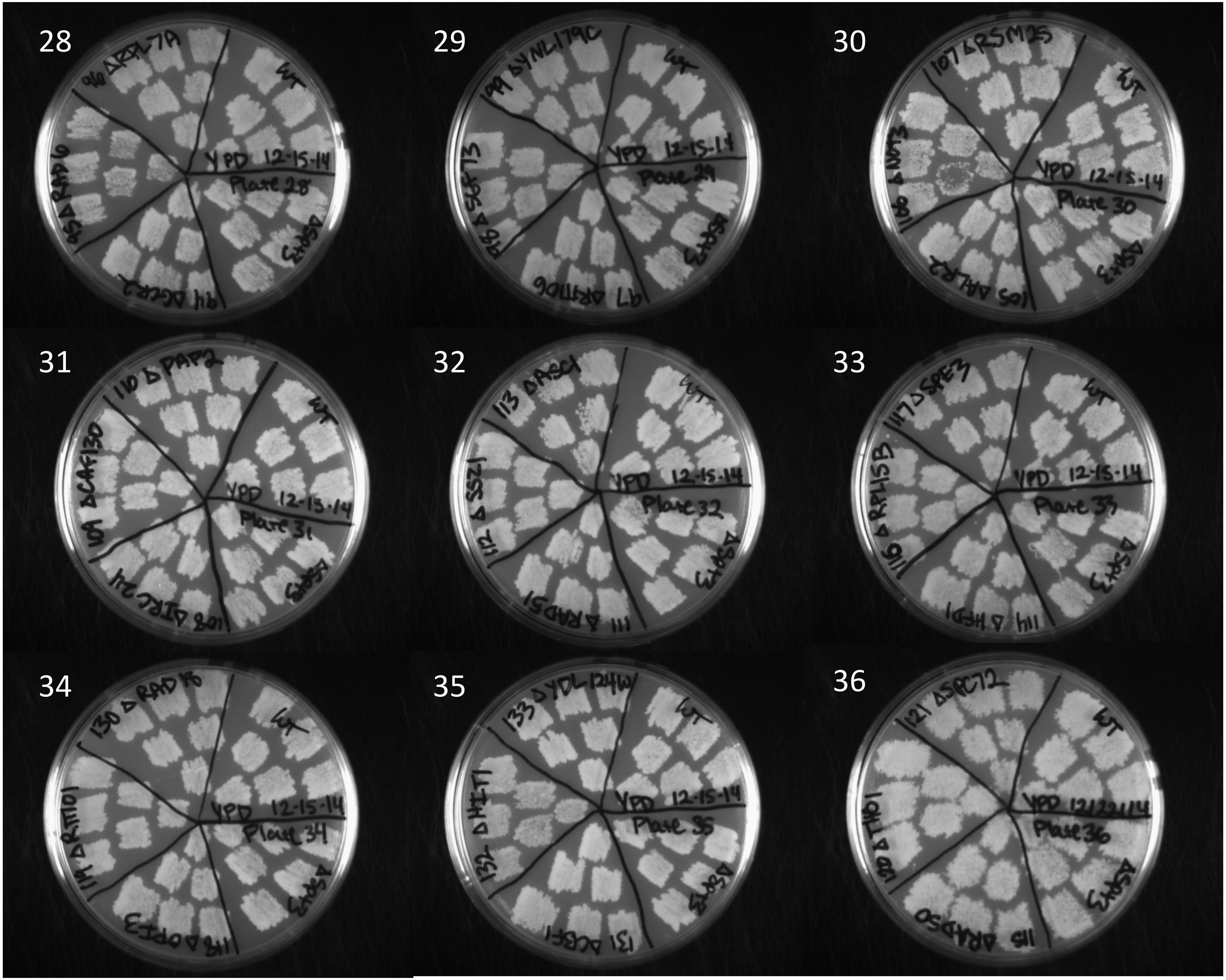

SC-HIS-LEU

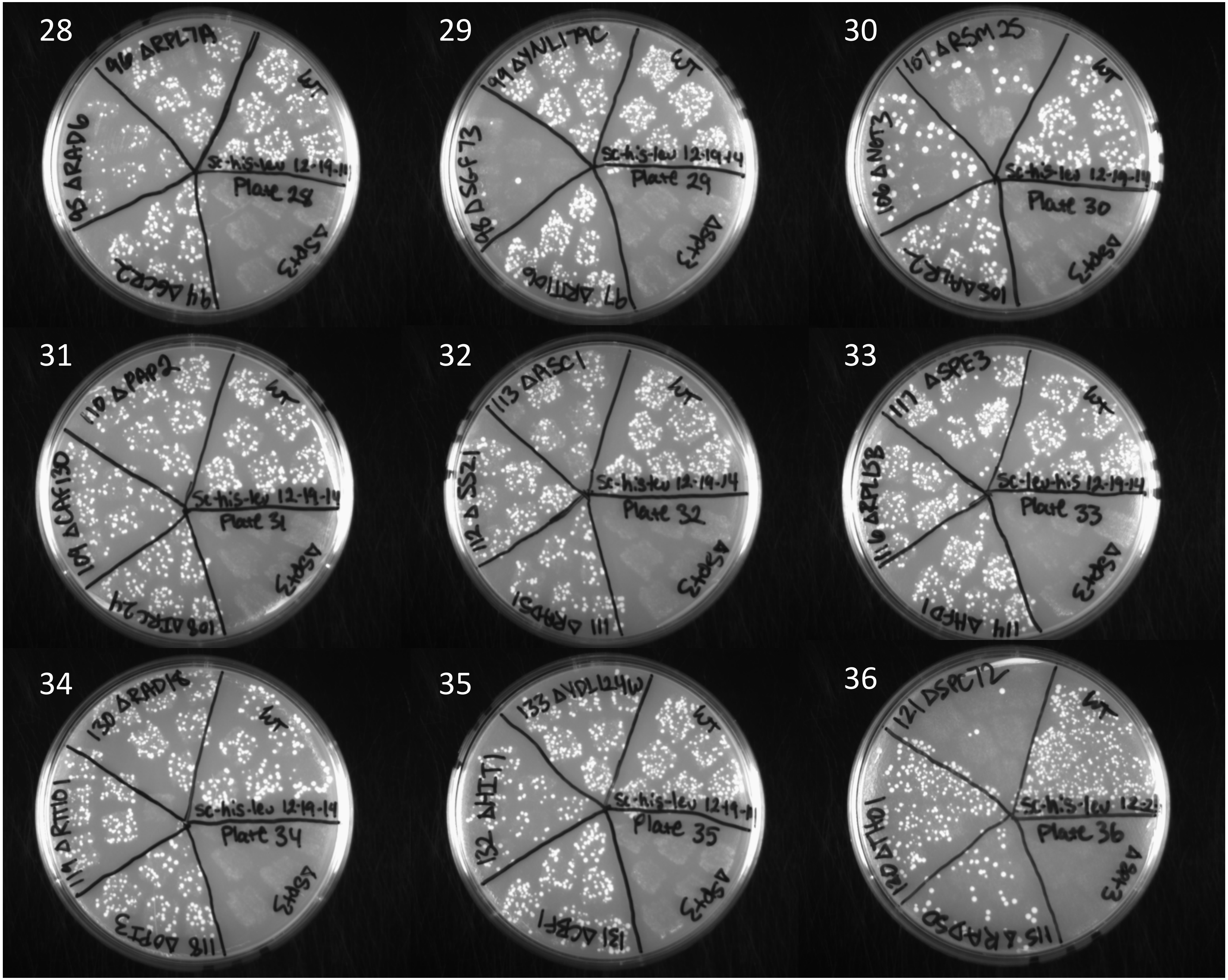
28
29
30
31
32
33
34
35
36
WT
Plate 28
Plate 29
Plate 30
Plate 31
Plate 32
Plate 33
Plate 34
Plate 35
Plate 36
sc-his-leu 12-19-14

SC-LEU

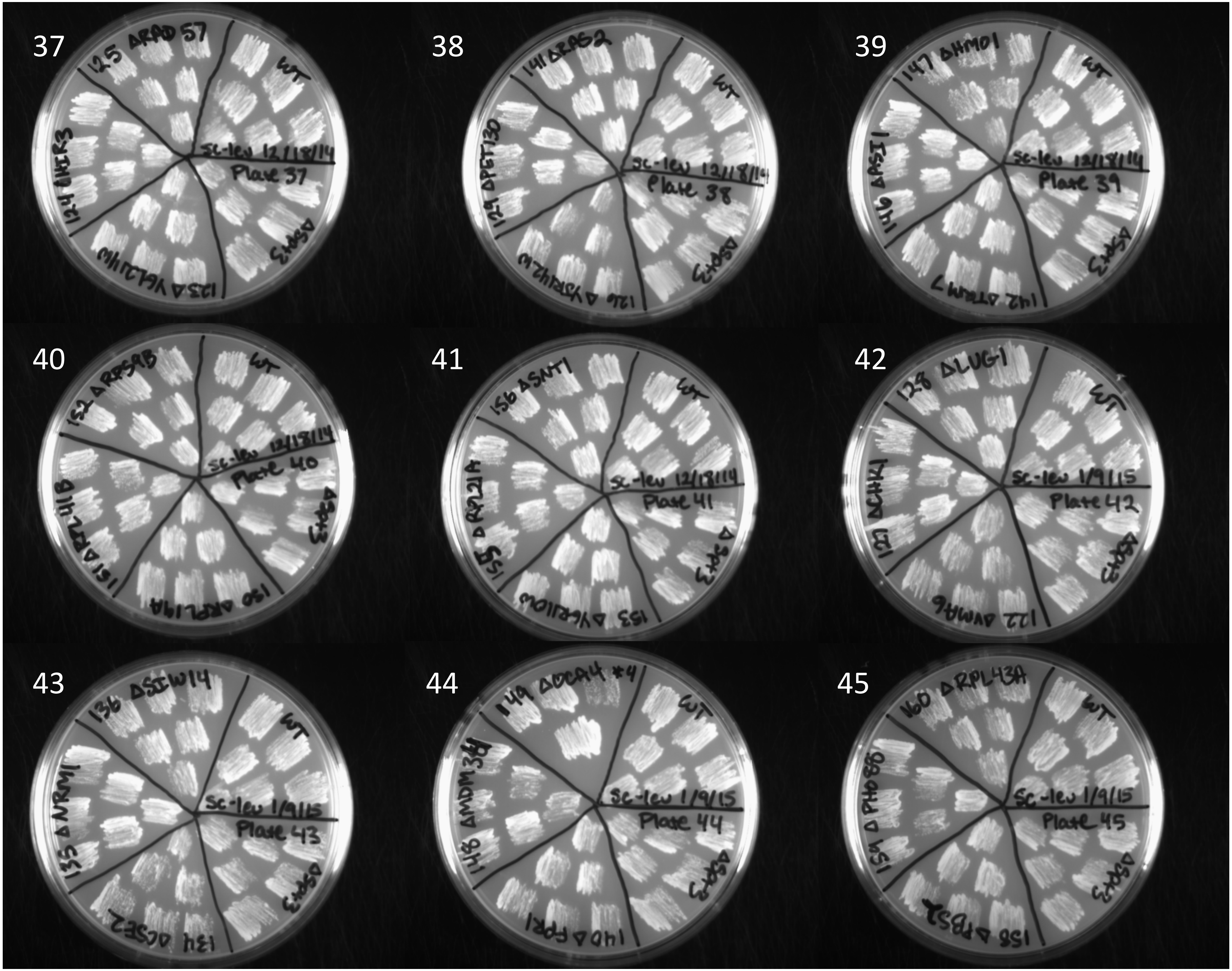

YPD

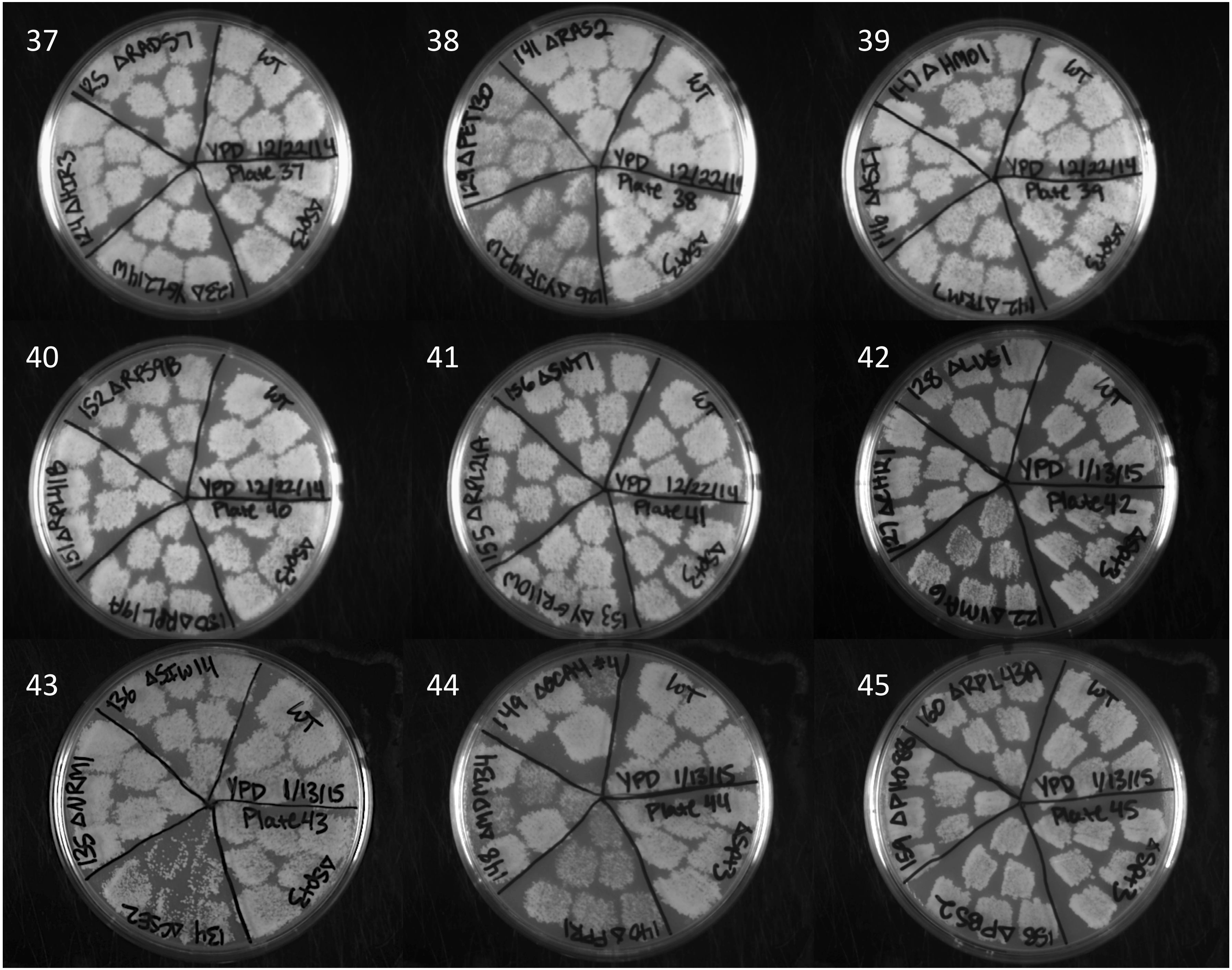

37
38
39
40
41
42
43
44
45
YPD 12/22/14
Plate 37
Plate 38
Plate 39
Plate 40
Plate 41
YPD 1/13/15
Plate 42
Plate 43
Plate 44
Plate 45
WT

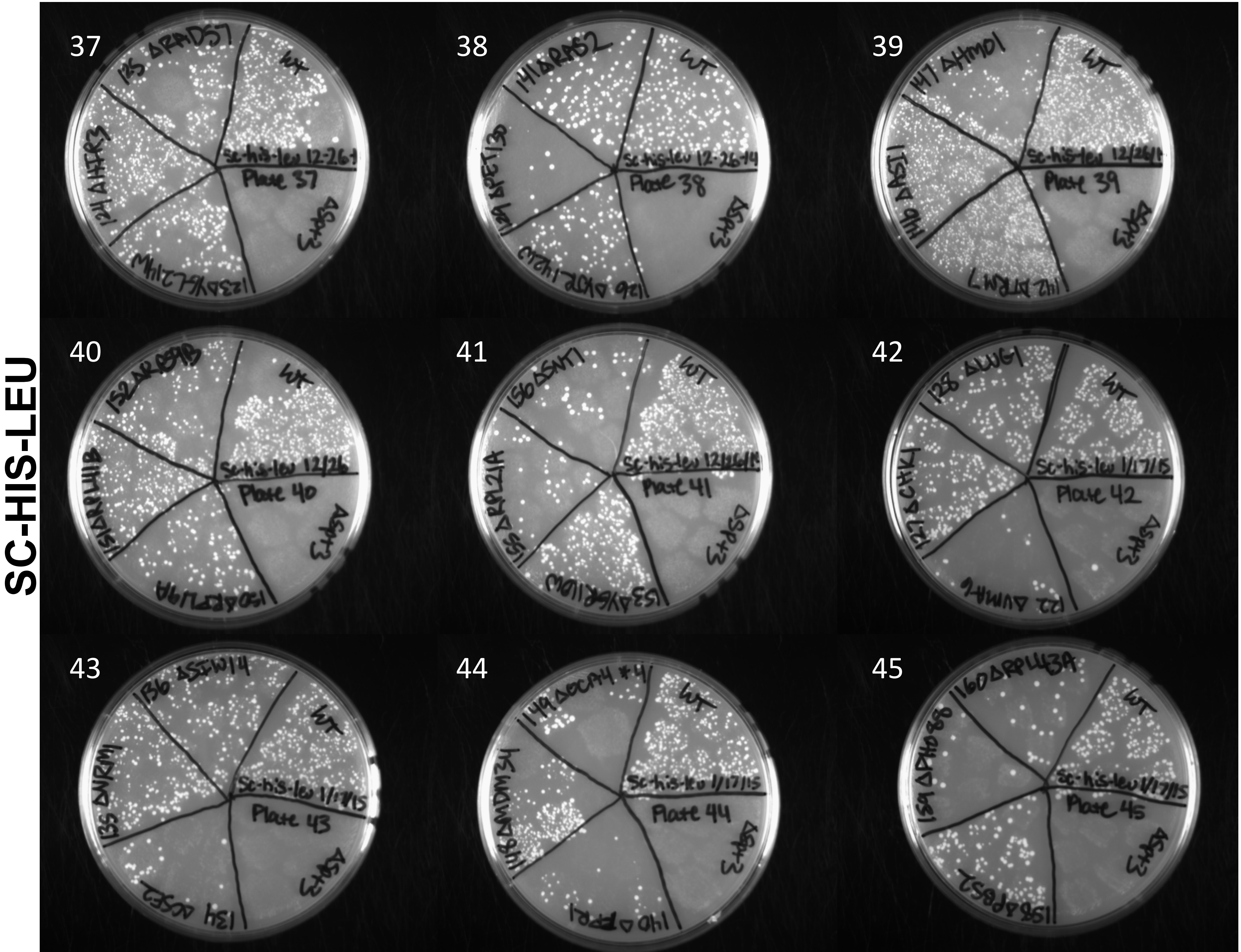
SC-HIS-LEU
37
38
39
40
41
42
43
44
45

**SC-LEU**

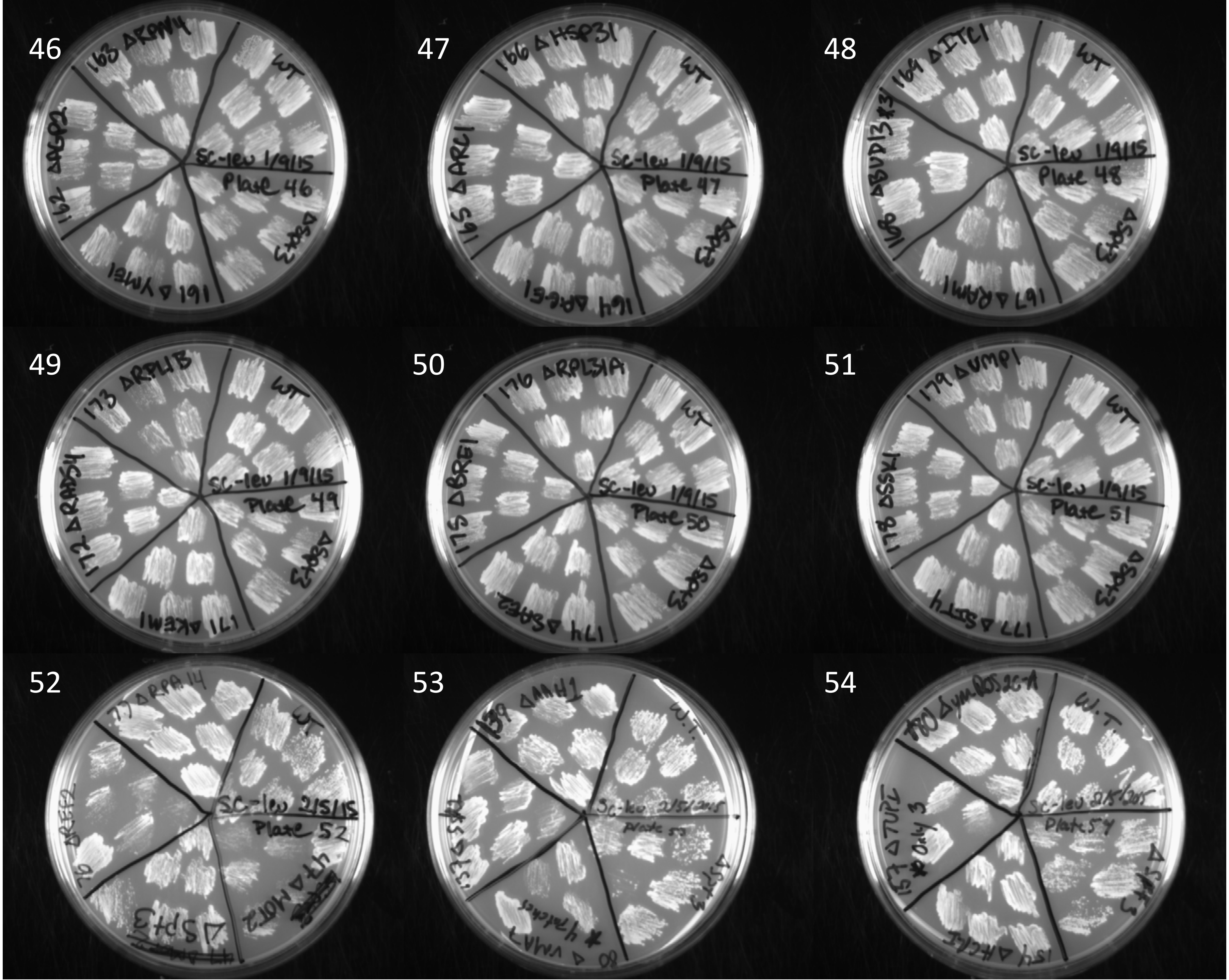

YPD

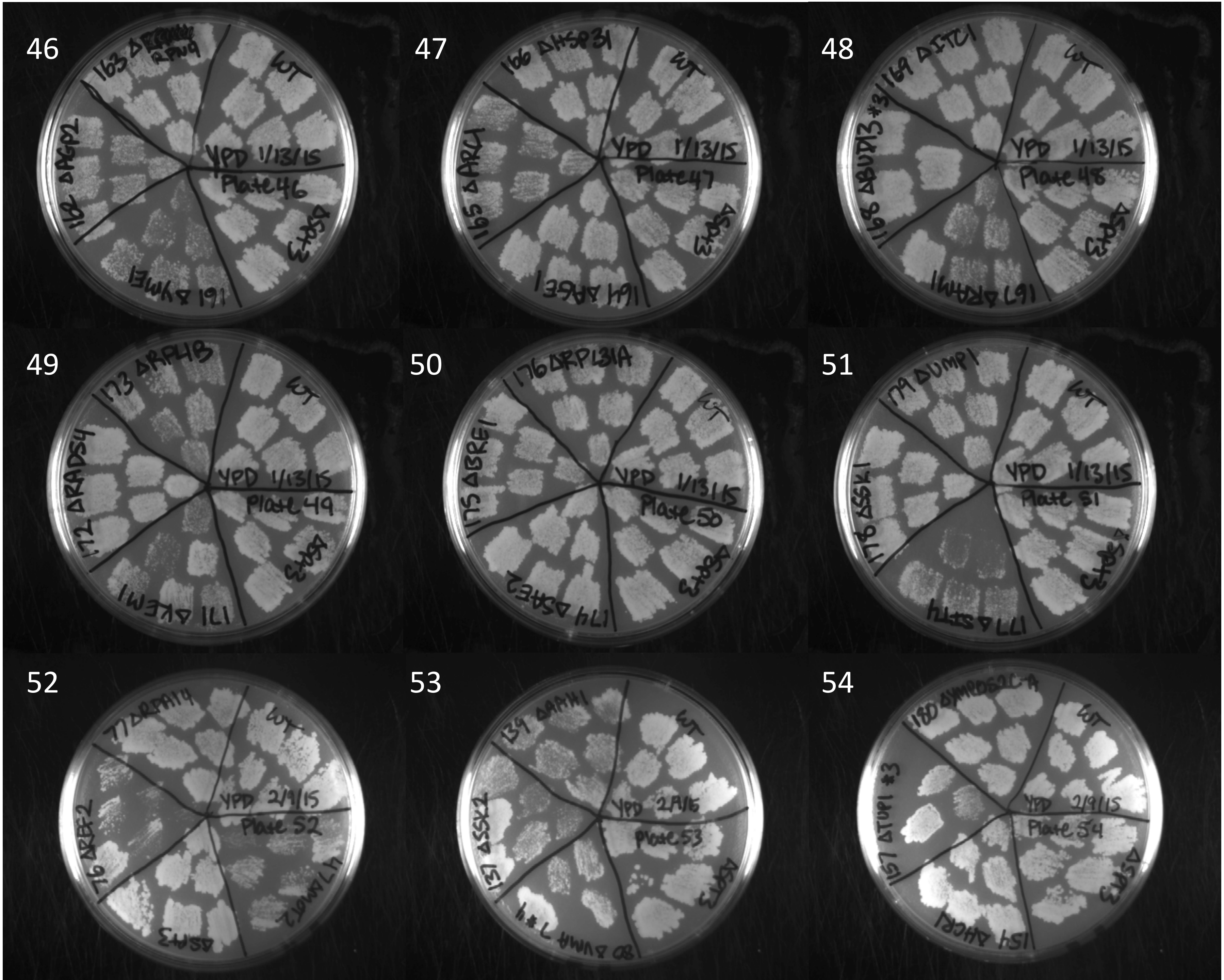

SC-HIS-LEU

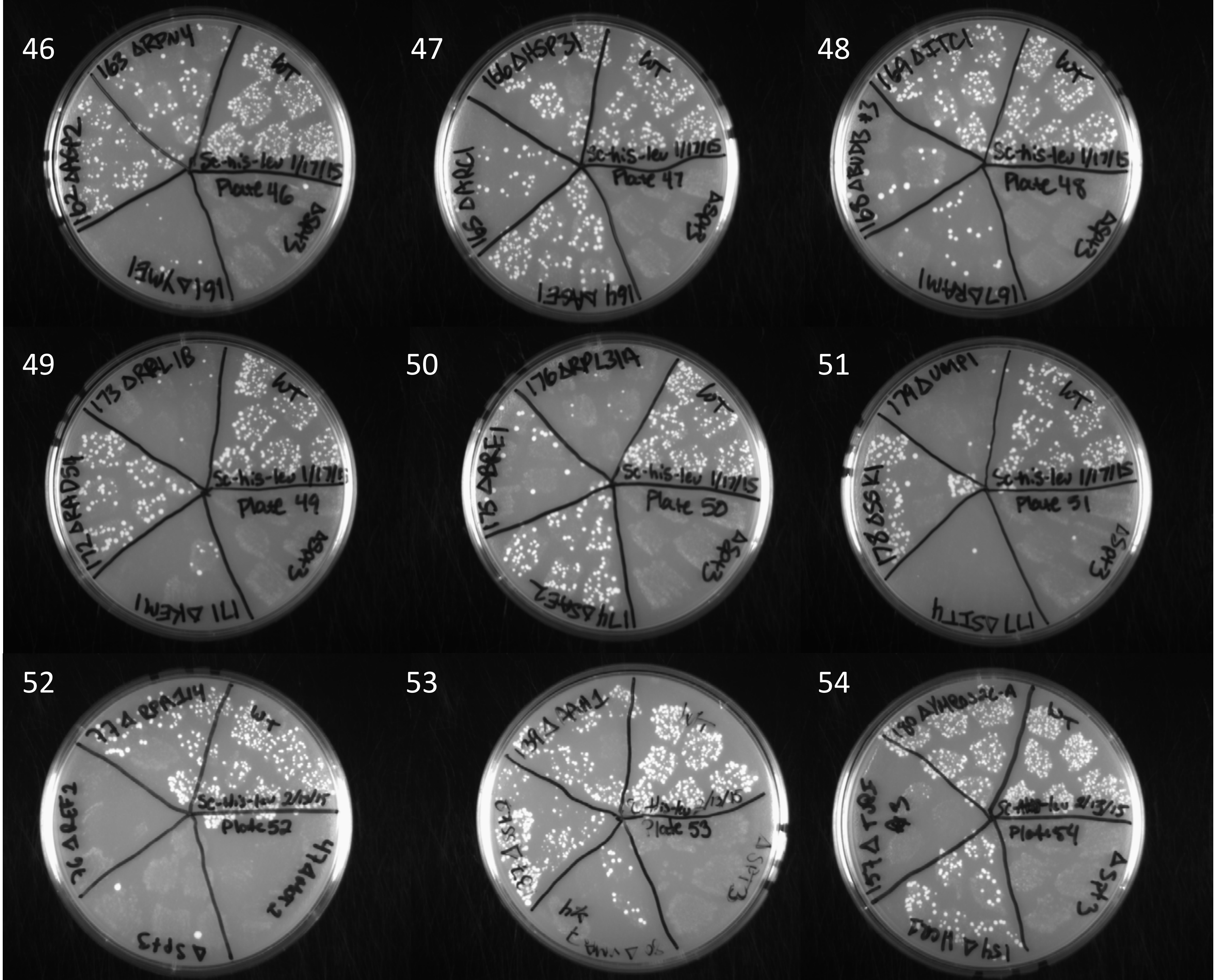

SC-LEU

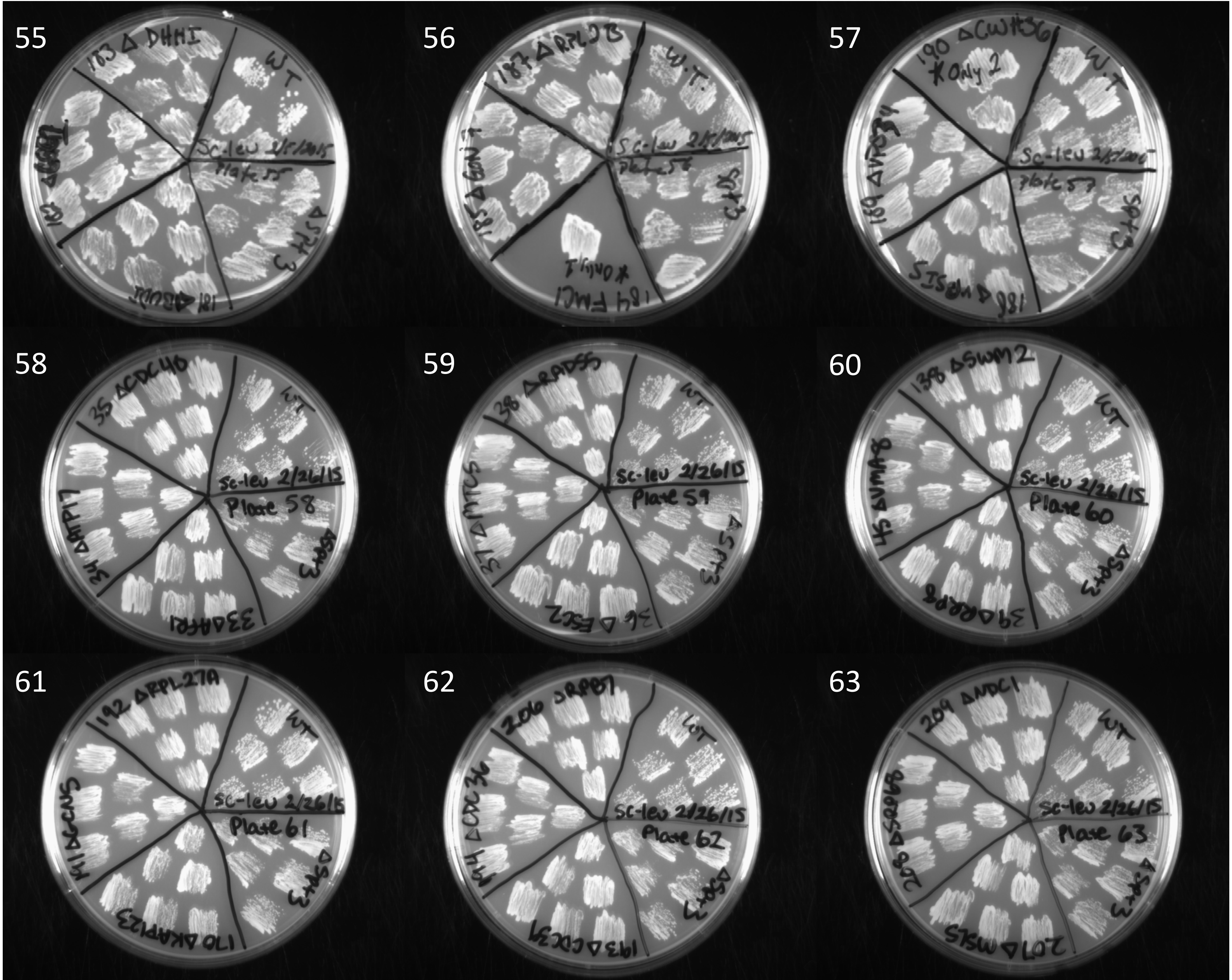

YPD

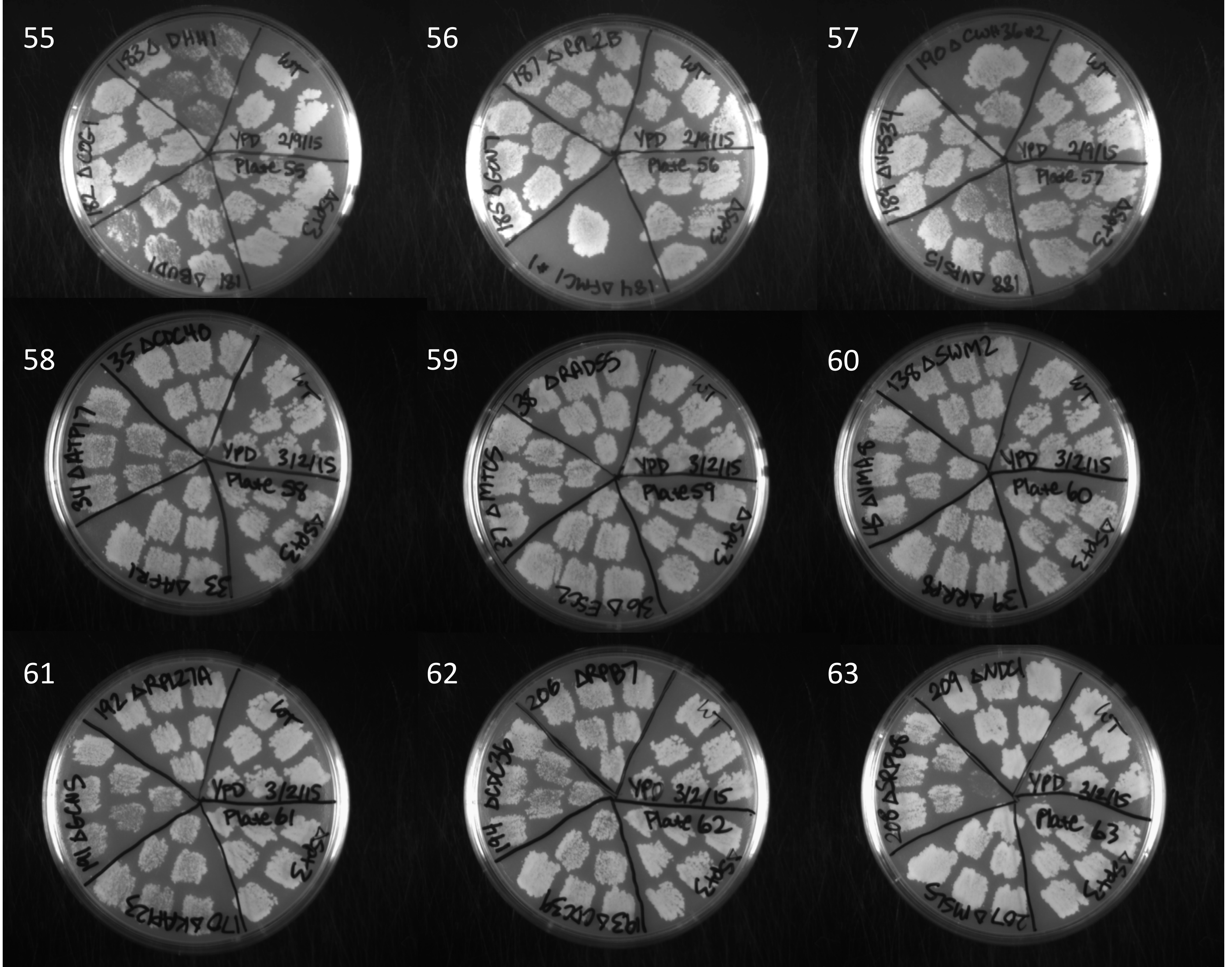
55
WT
YPD 2/19/15
Plate 55
56
WT
YPD 2/19/15
Plate 56
57
WT
YPD 2/19/15
Plate 57
58
WT
YPD 3/2/15
Plate 58
59
WT
YPD 3/2/15
Plate59
60
WT
YPD 3/2/15
Plate 60
61
WT
YPD 3/2/15
Plate 61
62
WT
YPD 3/2/15
Plate 62
63
WT
YPD 3/2/15
Plate 63

**SC-HIS-LEU**

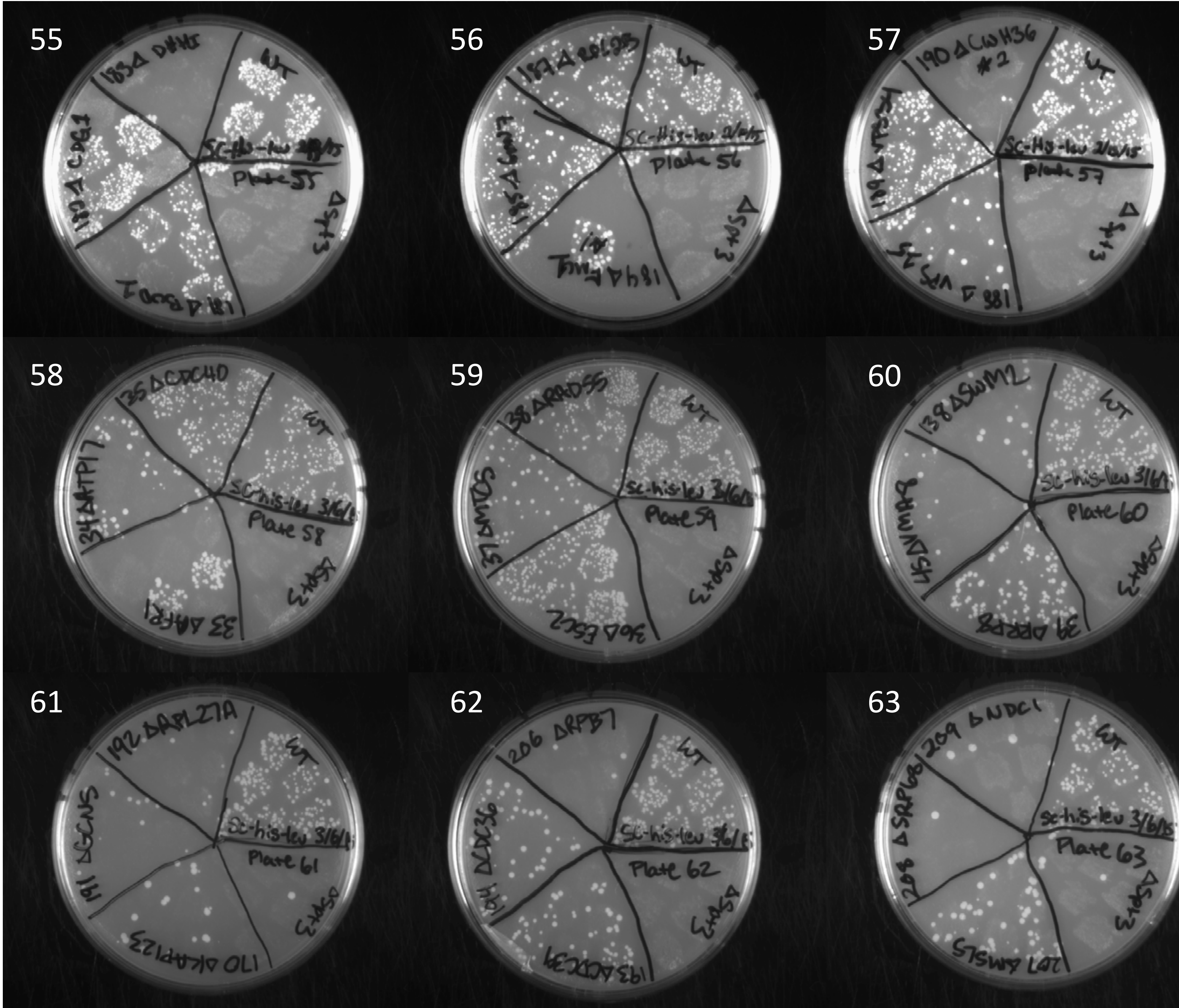

Supplement: Supplementary file 6 [file DataSheet1.PDF]
