# Supplementary material for: Reliance of Host-Encoded Regulators of Retromobility on Ty1 Promoter Activity or Architecture
Source: Front Mol Biosci. 2022 Jul 1;9:896215. doi: 10.3389/fmolb.2022.896215 (PMC9283973; doi:10.3389/fmolb.2022.896215)
Supplement: Supplementary file 8 [file Presentation2.PPTX]

## Slide 1
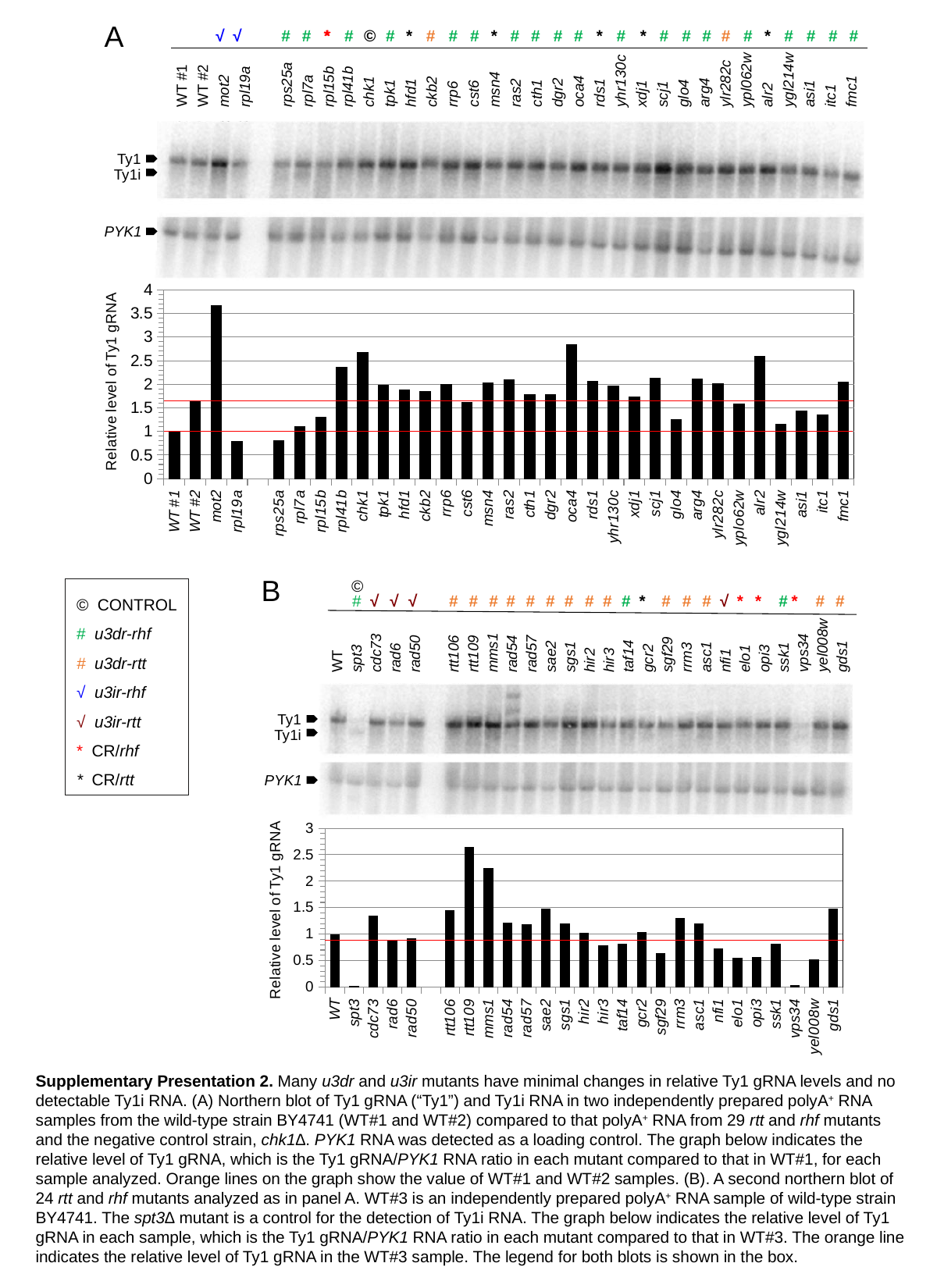

WT #1
WT #2
mot2
rpl19a
rps25a
rpl7a
rpl15b
rpl41b
chk1
tpk1
hfd1
ckb2
rrp6
cst6
msn4
ras2
cth1
dgr2
oca4
rds1
yhr130c
xdj1
scj1
glo4
arg4
ylr282c
ypl062w
alr2
ygl214w
asi1
itc1
fmc1
A
#
√
√
#
#
*
*
#
©
#
*
#
#
#
*
#
#
#
#
*
#
*
#
#
#
#
#
*
#
#
#
Ty1
Ty1i
PYK1
### Chart
| Category | Ty1 Corrected |
|---|---|
| WT #1 | 1.0 |
| WT #2 | 1.635562693538052 |
| mot2 | 3.673593983272684 |
| rpl19a | 0.784657799324557 |
| | None |
| rps25a | 0.799192357262969 |
| rpl7a | 1.096209559262676 |
| rpl15b | 1.305114505228721 |
| rpl41b | 2.361977323971043 |
| chk1 | 2.671521304266438 |
| tpk1 | 1.983717585041526 |
| hfd1 | 1.884279194128496 |
| ckb2 | 1.848348477105502 |
| rrp6 | 1.999306483195332 |
| cst6 | 1.611486202309669 |
| msn4 | 2.025903068668083 |
| ras2 | 2.094393793097204 |
| cth1 | 1.787000742937011 |
| dgr2 | 1.787888230990843 |
| oca4 | 2.845245460344824 |
| rds1 | 2.054325366339414 |
| yhr130c | 1.958241030875041 |
| xdj1 | 1.732592140463397 |
| scj1 | 2.134665089644173 |
| glo4 | 1.255506632883536 |
| arg4 | 2.119027402596531 |
| ylr282c | 2.004077336260302 |
| yplo62w | 1.576554223773147 |
| alr2 | 2.583272905744807 |
| ygl214w | 1.15001746151316 |
| asi1 | 1.426179513691052 |
| itc1 | 1.350255115808257 |
| fmc1 | 2.044260380876003 |B
WT
spt3
cdc73
rad6
rad50
rtt106
rtt109
mms1
rad54
rad57
sae2
sgs1
hir2
hir3
taf14
gcr2
sgf29
rrm3
asc1
nfi1
elo1
opi3
ssk1
vps34
yel008w
gds1
©
#
√
√
√
#
#
#
#
#
#
#
#
#
#
*
#
#
#
√
*
*
#
*
#
#
Ty1
Ty1i
PYK1
© CONTROL
# u3dr-rhf
# u3dr-rtt
√ u3ir-rhf
√ u3ir-rtt
* CR/rhf
* CR/rtt
### Chart
| Category | Ty1 Corrected |
|---|---|
| WT | 1.0 |
| spt3 | 0.028222272844847 |
| cdc73 | 1.34386657610972 |
| rad6 | 0.878199540471056 |
| rad50 | 0.923616345985161 |
| | None |
| rtt106 | 1.450069224399374 |
| rtt109 | 2.650481148852871 |
| mms1 | 2.247013938472384 |
| rad54 | 1.21763781550986 |
| rad57 | 1.19096272403532 |
| sae2 | 1.478376387535649 |
| sgs1 | 1.20687229413016 |
| hir2 | 1.029081762493357 |
| hir3 | 0.791064796648919 |
| taf14 | 0.819297180537785 |
| gcr2 | 1.034542953879526 |
| sgf29 | 0.639182603015723 |
| rrm3 | 1.297903173960061 |
| asc1 | 1.205184402736842 |
| nfi1 | 0.730163410283325 |
| elo1 | 0.550261474919082 |
| opi3 | 0.569819027174794 |
| ssk1 | 0.821444794450151 |
| vps34 | 0.0324320772960184 |
| yel008w | 0.525131534992753 |
| gds1 | 1.482675455752409 |Supplementary Presentation 2. Many u3dr and u3ir mutants have minimal changes in relative Ty1 gRNA levels and no detectable Ty1i RNA. (A) Northern blot of Ty1 gRNA (“Ty1”) and Ty1i RNA in two independently prepared polyA+ RNA samples from the wild-type strain BY4741 (WT#1 and WT#2) compared to that polyA+ RNA from 29 rtt and rhf mutants and the negative control strain, chk1∆. PYK1 RNA was detected as a loading control. The graph below indicates the relative level of Ty1 gRNA, which is the Ty1 gRNA/PYK1 RNA ratio in each mutant compared to that in WT#1, for each sample analyzed. Orange lines on the graph show the value of WT#1 and WT#2 samples. (B). A second northern blot of 24 rtt and rhf mutants analyzed as in panel A. WT#3 is an independently prepared polyA+ RNA sample of wild-type strain BY4741. The spt3∆ mutant is a control for the detection of Ty1i RNA. The graph below indicates the relative level of Ty1 gRNA in each sample, which is the Ty1 gRNA/PYK1 RNA ratio in each mutant compared to that in WT#3. The orange line indicates the relative level of Ty1 gRNA in the WT#3 sample. The legend for both blots is shown in the box.
